# Increased sleep duration and delayed sleep timing during the COVID-19 pandemic

**DOI:** 10.1038/s41598-022-14782-x

**Published:** 2022-06-29

**Authors:** Robin K. Yuan, Kirsi-Marja Zitting, Liyaan Maskati, Jeff Huang

**Affiliations:** 1 Division of Sleep and Circadian Disorders, Department of Medicine, Brigham and Women’s Hospital, and Division of Sleep Medicine, Harvard Medical School, 221 Longwood Avenue, Boston, MA 02115 USA; 2 Department of Computer Science, Brown University, 115 Waterman Street, Providence, RI 02912 USA

**Keywords:** Diseases, Physiology

## Abstract

Many studies have examined how the 2019 Coronavirus Disease (COVID-19) has impacted sleep health. Early evidence suggests that lockdown policies worldwide have led to changes in sleep timing, duration, and quality; however, few studies have attempted to look at the longer-term effects across multiple countries in a large data set. This study uses self-reported data from 64,858 users of the Sleep As Android smartphone application from around the world over a 24-month period in 2019 to 2020. We found a significant but modest increase in time in bed (TIB), as well as a significant delay in sleep timing that was especially prominent on weekdays. While this effect persisted throughout the year, differences in sleep timing were more widespread and pronounced in the earlier months of the pandemic. We observed a small overall increase in TIB when comparing 2020 to 2019, but these changes depended on location and time of year, suggesting that sleep duration may have more closely tracked the progression of the pandemic in each country. Our findings suggest that pandemic-induced changes in lifestyle, such as remote work and lockdown policies, may have facilitated later sleep timing but that these changes may diminish as restrictions are lifted.

## Introduction

The ongoing COVID-19 pandemic has had wide-ranging effects on sleep health around the world. These effects include changes in sleep timing, sleep duration, and sleep quality^1–4^. Several studies have found decreases in sleep quality, especially in healthcare and frontline essential workers who report experiencing increased insomnia and stress^5–11^. This effect was especially pronounced early in the pandemic among the general population as well^1,4,12–15^. Indeed, we found that in the U.S., Google searches for “insomnia” increased by 58% in March through May 2020 compared to the same time frame in the previous three years^16^, suggesting that sleep problems may have increased in the general population during the early months of the pandemic.

While studies have identified that some people experienced shortened sleep durations^4,8^, there is ample evidence that sleep duration has increased during the pandemic for a substantial proportion of the population, especially after more countries enacted lockdown policies^2,3,17^. In an online survey of 7,517 respondents in 40 countries between April 4th–May 6th, 2020, average sleep duration was found to increase by 26 min on work days^17^. Similarly, surveys of roughly 1000 participants in Argentina^3^ and Italy^18^ found that average sleep duration in non-healthcare workers increased during the pandemic.

Perhaps the most striking impact of the pandemic on sleep health has been the dramatic shift in sleep timing. Studies across multiple countries and in different subject populations have consistently found that people shifted their sleep timing later, perhaps because of the increased prevalence of remote work and school during the lockdown^2,3,17,19^. Studies in university students^1,2^ found that sleep times were delayed by 25–50 min on average. Studies of the general population report similar delays especially on week days^4,20–22^, suggesting that social work schedules may be better aligned with internal chronotype during the pandemic^17^. Social jetlag, the discrepancy between people’s sleep timing on work days and free days (typically weekdays and weekends, respectively), is a well-known phenomenon^23^. In general, people report going to bed and waking earlier on weekdays compared to weekends. During the pandemic however, multiple studies found that social jetlag decreased—in other words, differences in week day and weekend sleep timing diminished, predominantly because of a delay in week day wake times^1–3,17^.

Thus far, most published studies on the impact of the pandemic on sleep health have examined fairly acute, early effects of the pandemic, looking at periods ranging from a few days to months in spring of 2020^22,24,25^. Now that the pandemic has been ongoing for almost two years, it is increasingly important to examine some of the longer-term consequences and patterns in sleep health. Moreover, early studies largely focused on the impact within a single country with limited sample sizes. However, as the COVID-19 pandemic has spread around the world, we are now able to investigate the cumulative global impact of the pandemic on sleep habits. In this study, we examine self-reported data on bed and wake times from over 50,000 users of the Sleep As Android smartphone application in 2019 (prior to the pandemic) as well as throughout the full year of 2020.

## Results

### Global changes in sleep duration and timing between 2019 and 2020

We calculated time in bed (TIB) and midpoint of sleep (MOS) using the median bed and wake times for each day in 2019 and 2020 in 57 total countries that each had data from a minimum of 100 users per year. To examine global changes in sleep between 2019 and 2020, we compared median TIB, MOS, bedtime, and waketime for 2019 vs. 2020. We found significant differences between 2019 and 2020 in TIB, MOS, bedtime, and waketime (*p* < 0.0001 for all). TIB increased by approximately 5 min in 2020 compared to 2019, while MOS, bedtime, and waketime all shifted to later times in 2020 compared to 2019.

**Figure 1:**
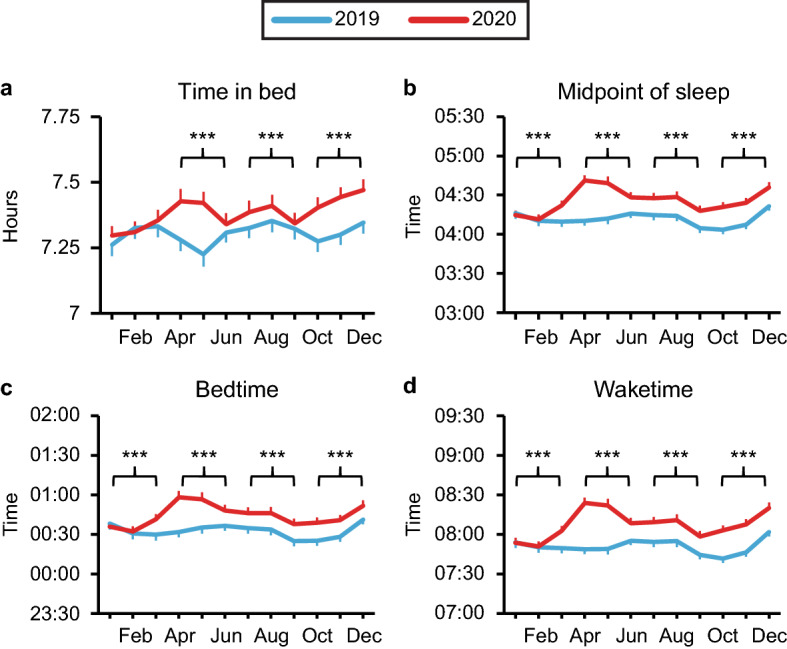
Global changes in sleep duration and timing between 2019 and 2020. Worldwide average time in bed (**a**) and midpoint of sleep (**b**) were calculated from self-reported bedtimes (**c**) and waketimes (**d**) for each quarter in 2019 (blue) and 2020 (red). Bonferroni-adjusted p values for comparisons between each quarter of 2019 to the corresponding quarter of 2020 are indicated as follows: *p < 0.05, **p < 0.01, ***p < 0.001. Means ± SEM are shown.

To investigate whether these changes occurred at different points in the year, we next broke each year down into quarters (Jan–Mar; Apr–Jun; Jul–Sept; Oct–Dec) and compared each quarter of 2019 to the corresponding quarter of 2020 (Fig. 1). There was no significant change in TIB during the 1st quarter of 2020, but there was a modest increase of 3–8 min in TIB during the 2nd, 3rd, and 4th quarters of the year (*p* < 0.0001 for all) compared to the same quarters in 2019. Furthermore, we found significant delays in all sleep timing outcomes (MOS, bedtime, and waketime) in all quarters of 2020 compared to 2019 (*p* < 0.0001 for all), with the largest delays occurring in the 2nd quarter of the pandemic and the smallest in the first quarter. The shift in MOS ranged from 4 to 23 min, while the shift in bedtime ranged from 4 to 20 min, and the shift in waketime ranged from 5 to 27 min.

### Changes in sleep duration and timing between 2019 and 2020 in 20 representative countries

**Figure 2:**
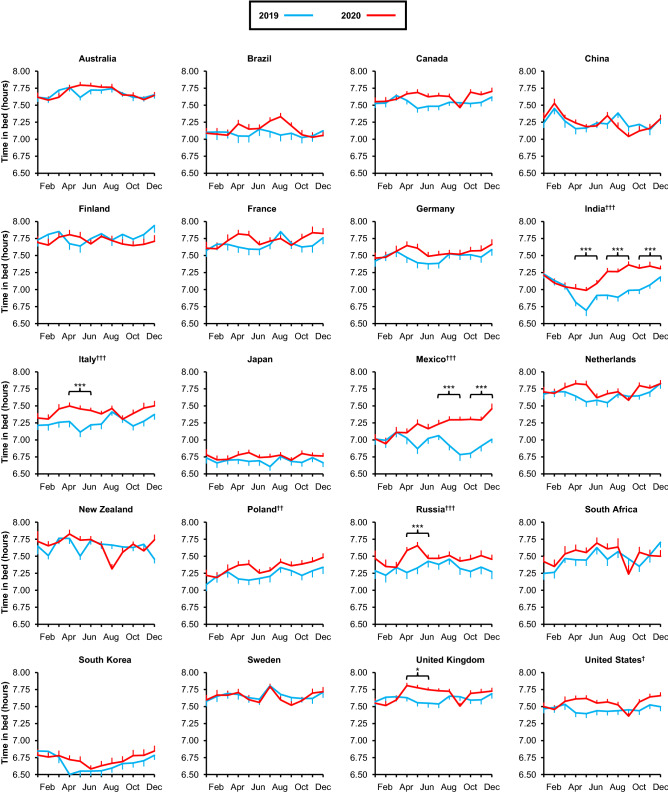
Self-reported time in bed from 20 countries in year 2019 and 2020. Average time in bed (TIB) was calculated from self-reported bed and wake times for each day in 2019 (blue) and 2020 (red) for each country. Bonferroni-adjusted p values comparing average TIB in each quarter of 2019 to the corresponding quarter of 2020 are indicated as follows: *p < 0.05, **p < 0.01, ***p < 0.001. Bonferroni-adjusted p values comparing overall TIB in 2019 to 2020 is indicated next to each country name as follows: ^†^*p* < 0.05, ^††^*p* < 0.01, ^†††^*p* < 0.001. Means ± SEM are shown.

We next examined TIB, MOS, bedtime, and waketime in 20 representative countries, selected to capture as much geographic diversity as possible within our dataset. Although 15 out of the 20 countries showed an increase in TIB, most were not significant after correction for multiple comparisons. Only India (*p* < 0.0001), Italy (*p* < 0.0001), Mexico (*p* < 0.0001), Poland (*p* = 0.002), Russia (*p* < 0.0001), and the U.S. (*p* = 0.02) showed a significant increase in TIB after Bonferroni adjustment (Fig. 2).

Changes in sleep timing were more widespread, with 17 countries out of 20 showing a significant delay in MOS (*p* < 0.05 for all), 19 countries showing a significant delay in bedtimes (*p* < 0.05 for all), and 16 countries showing a significant delay in waketimes (*p* < 0.05 for all) in 2020 compared to 2019. Only South Korea showed no change in any of the three sleep timing measures. Additionally, Japan showed no change in MOS or waketime, and Sweden and New Zealand showed no change in waketime.

To better examine how the changes in sleep corresponded with the time course of the pandemic in each country, we again compared each quarter of 2019 to the corresponding quarter of 2020 in each of the 20 countries. In the first quarter of 2020, we detected no significant differences in TIB in any countries, and no significant delays in sleep timing except for China and Finland, both of which had significant delays in MOS and bedtime (*p* < 0.05 for all).

Sleep changes were most prevalent in the 2nd quarter of 2020. TIB was significantly greater compared to the 2nd quarter of 2019 in India (*p* = 0.01), Italy (*p* < 0.0001), Russia (*p* < 0.0001), and the United Kingdom (*p* = 0.048). Strikingly, 15 countries showed significant delays in MOS (*p* < 0.0001 for all), 16 countries had significant bedtime delays (*p* < 0.0001 for all), and 15 countries had significant delays in waketimes (*p* < 0.05 for all).

In the 3rd quarter of 2020, TIB was significantly greater than during the 3rd quarter of 2019 in only two countries, India (*p* < 0.0001) and Mexico (*p* < 0.0001). India also had significant delays in all three sleep timing measures, as did South Africa (*p* < 0.0001 for all). Only five other countries (Australia, Finland, Mexico, UK, and US) had significant delays in MOS (*p* < 0.05 for all). We found significant bedtime delays in nine other countries (*p* < 0.05 for all), and waketime delays in only Mexico (*p* < 0.0001), in addition to India and South Africa.

Finally, during the 4th quarter of 2020, only India and Mexico had significantly greater TIB compared to the 4th quarter of 2019 (*p* < 0.0001 for both). However, five countries (Finland, Russia, South Africa, UK, and US) had significant delays in MOS (*p* < 0.05 for all), seven countries had delays in bedtime (*p* < 0.0001), and five countries (India, Mexico, Russia, South Africa, US) had delays in wake times.

### Changes in sleep duration and timing between 2019 and 2020 in the United States

**Figure 3:**
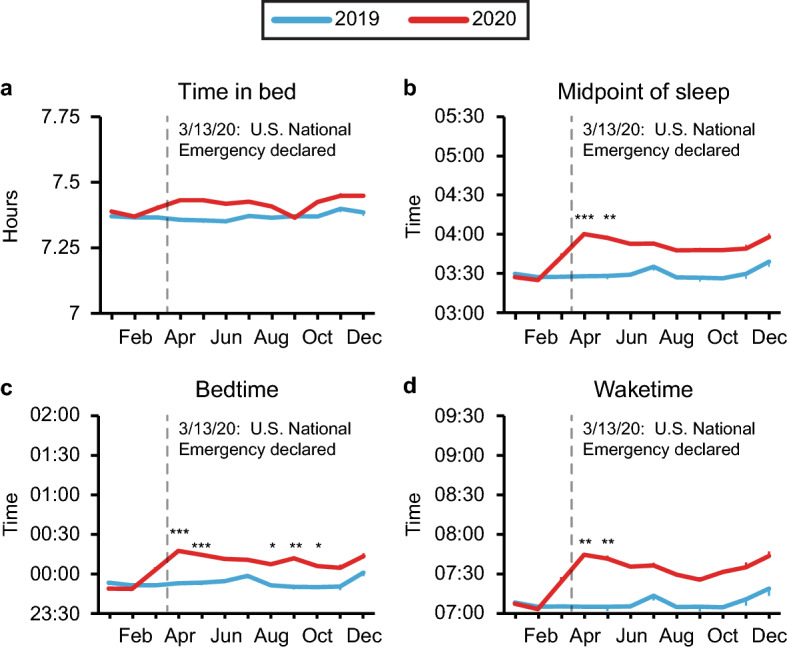
Self-reported sleep duration and timing in the United States during the years 2019 and 2020. Average time in bed (**a**) and midpoint of sleep (**b**) were calculated from self-reported bedtimes (**c**) and waketimes (**d**) for each month in 2019 (blue) and 2020 (red) in the United States. Bonferroni-adjusted p values for comparisons between each month of 2019 to the corresponding month of 2020 are indicated as follows: *p < 0.05, **p < 0.01, ***p < 0.001. Means ± SEM are shown.

To more closely examine how these changes in sleep timing and duration corresponded to the time course of the pandemic, we compared each month of 2019 to each month of 2020 specifically within the United States (Fig. 3). In the U.S., the first confirmed case of COVID-19 was January 2020, with an initial surge in COVID-related deaths occurring in April. COVID-19 was declared a national emergency on March 13, with the first state-wide stay-at-home order issued in California on March 19th. Similar lockdown measures followed in other states, contributing to the subsequent reduction in number of COVID-related deaths in the third quarter of the year. By the end of the year however, the U.S. had entered its second wave of COVID-19 infections.

Interestingly, we found no significant difference in TIB between 2020 to 2019 in any of the months. Instead, we found a significant delay of ~ 29 min in MOS in April (*p* < 0.0001) and May (*p* = 0.0012) of 2020 compared to 2019, shortly after the start of the pandemic in the U.S. Bedtimes were also significantly delayed in April and May (*p* < 0.0001 for both), as well as in August (*p* = 0.024), September (*p* = 0.006), and October (*p* = 0.0252). These bedtime delays ranged from 23 min (April) to 16 min (October). Waketime was significantly delayed in 2020 compared to 2019 only during the months of April and May (*p* = 0.006 for both), by approximately 35 min. Our results suggest that in the United States, the pandemic caused shifts primarily in the timing of sleep.

### Changes in sleep duration and timing by day of the week during the pandemic

**Figure 4:**
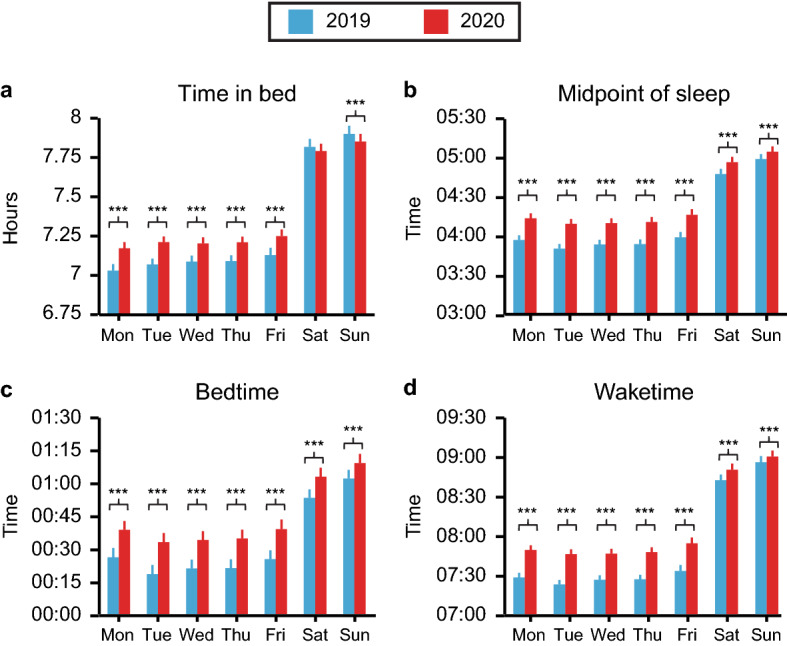
Self-reported time in bed and sleep timing by day of the week during the pandemic. Average time-in-bed (**a**) and midpoint of sleep (**b**) were calculated from self-reported bed (**c**) and wake (**d**) times for each day of the week in 2019 (blue) and 2020 (red). Means and SEMs are shown. Bonferroni-adjusted p values for comparisons between each day of 2019 and the corresponding day of 2020 are indicated as follows: *p < 0.05, **p < 0.01, ***p < 0.001.

We next examined global trends in TIB, bedtime, wake time, and MOS on each day of the week in 2020 compared to the corresponding day of the week in 2019 (Fig. 4). There was a significant increase of roughly 7–8 min in TIB in 2020 compared to 2019 on all week days (*p* < 0.0001 for Mon-Fri), and a modest but significant increase of roughly 3 min on Sunday (*p* < 0.0001). TIB did not significantly increase on Saturday. MOS shifted significantly later on all days (*p* < 0.0001 for all) by roughly 16–19 min on week days and about 6–8 min on Saturday and Sunday. Similarly, bedtimes were significantly delayed by approximately 13–15 min on week days (*p* < 0.0001 for Mon-Fri) and 7–10 min on Saturday and Sunday (*p* < 0.0001 for both). Finally, waketimes were also significantly delayed on all days (*p* < 0.0001 for all), ranging from a 20–23 min delay on Monday through Friday to just 4–8 min on Saturday and Sunday.

**Figure 5:**
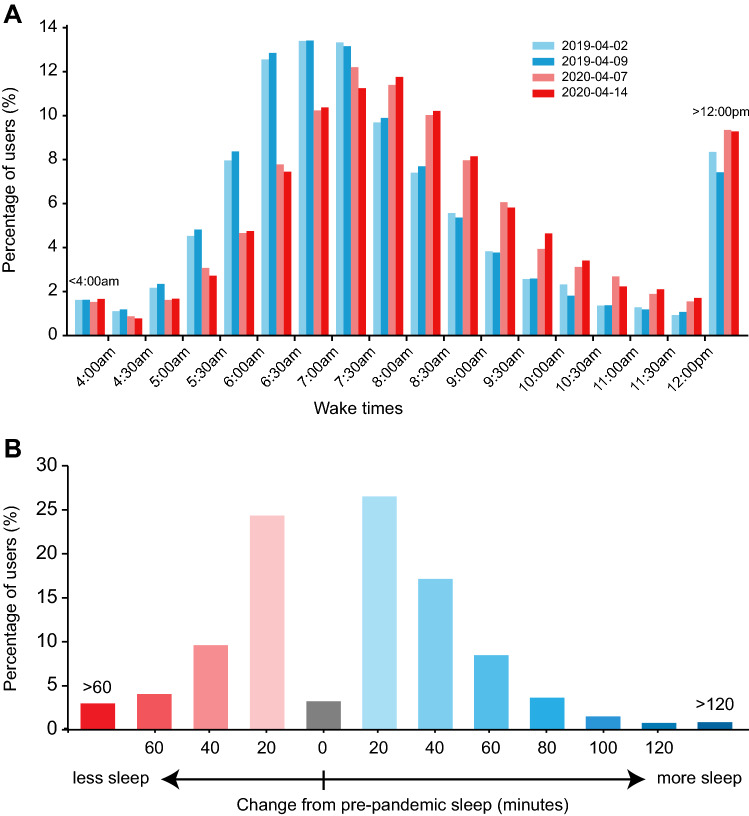
Overall effect of the pandemic on weekday waketimes and sleep duration. (**a**) Self-reported waketimes from the first two Tuesdays in April 2019 and 2020 are indicated in blue and red respectively, from users around the world. (**b**) Change in weekday hours slept during the pandemic in a subset of 12,551 individuals with data from both 2019 and 2020. No change (gray bar) was defined as < 1 min change from pre-pandemic sleep. Increases are indicated in blue and decreases are indicated in red.

To examine the acute phase of pandemic more closely, we selected the first two Tuesdays in April to use as representative weekdays from each year near the beginning of the pandemic. These days were selected because there were no major holidays, events, or weekday-weekend transitions that might impact sleep timing. We then looked at the distribution of waketimes on these days in 2020 compared to 2019 in the full dataset (64,858 users). As expected, there was a wide range of wake times both before and during the pandemic. However, the distribution of wake times shifted later during the pandemic. More users woke up at or after 7am in 2020 during the pandemic, compared to the percentage of users who woke up at a similar time in 2019. Prior to the pandemic, it was most common for users to wake up around 6:30-7am, whereas during the pandemic, the most common wake up time was 7–7:30am (Fig. 5A).

We also compared weekday sleep duration just prior to the pandemic (January 1 to March 15, 2020) to weekday sleep duration in the early phase of the pandemic (March 16-May 20, 2020) in a subset of users who reported data for both periods (12,551 individuals). Interestingly, during the acute phase of the pandemic, weekday sleep duration increased by more than 20 min for 32% of users overall during the pandemic, while 17% of users reported sleeping less than usual. The sleep duration of 51% of users remained within 20 min of their pre-pandemic sleep duration (Fig. 5B).

## Discussion

We examined average self-reported time in bed and sleep timing in 53,545 Sleep as Android users across 20 countries, as well as a larger cohort of 64,858 users all over the world, before and during the COVID-19 outbreak. Consistent with what has previously been reported in smaller cohorts, we found that self-reported sleep times shifted later during the COVID-19 outbreak, and that these changes were more prominent near the beginning of the year compared to later in the year. When we analyzed differences in sleep duration and sleep timing between 2020 and 2019 in individual countries, we found that these changes corresponded roughly with the time course of the pandemic. In Italy for example, we found increased TIB, delayed MOS, and delayed bedtimes and waketimes in April through June of 2020, following their first peak in COVID-19 cases. Similarly, we found changes in TIB as well as sleep timing in Russia in April through June of 2020 following their first peak in COVID-19 cases, and again in the last quarter of the year when they were undergoing their second wave. Moreover, almost all countries had significant sleep timing changes in the second quarter (April-June) of 2020, shortly after the WHO declared COVID-19 to be a pandemic and also coinciding with the first major COVID-19 wave in much of Europe. Sleep timing changes diminished during the second half of the year in many countries, but countries such as India, Mexico, and South Africa, which were not heavily impacted by the pandemic until late summer, continued to have significant changes in sleep timing during the fourth quarter of the year. Finally, while significant increases in time in bed and delays in sleep timing were present on almost all days of the week, the magnitude of these changes were greatest on week days and fairly modest on weekends, suggesting that people’s sleep schedules changed on work days but remained similar on free days.

While there have been several studies showing reduced sleep quality and duration in healthcare or other essential frontline workers^6,8,10,11^, most studies drawn from the general population have found increases in total sleep duration during the pandemic. Studies analyzing self-reported sleep from different smartphone applications found that the onset of the COVID-19 pandemic was associated with increases in sleep duration^22,24,25^. Similarly, when we looked at the acute effect of the pandemic (March -May) in a global cohort of Sleep As Android users, we found that 32% of people slept more than 20 min longer than their usual sleep duration, although 17% were sleeping more than 20 min less than usual per day on weekdays. This non-uniform change in sleep duration during the pandemic is not unexpected. Other studies have found that changes in sleep quality during the COVID-19 lockdown differed significantly depending on pre-pandemic sleep quality, such that pre-pandemic good sleepers were more likely to experience a decrease in sleep quality during the pandemic, while some with severe insomnia symptoms showed an improvement in sleep^4,26^. Moreover, in contrast to many other studies, Salfi et al.^27^ found that sleep quality and duration were negatively impacted in Italy during the first and second major COVID-19 outbreaks, suggesting that changes in sleep duration may depend on the severity of the pandemic in particular regions. It is also possible that undetected changes in sleep latency could be influencing the reported sleep durations –many studies, ours included, use time in bed as a proxy for sleep duration; however, time in bed will likely overestimate actual sleep duration.

The changes in sleep timing, particularly in the early part of the pandemic, could be partially due to the increased prevalence of remote work following the lockdown measures imposed in response to the pandemic. In the U.S., a recent Pew Research Center survey found that 71% of the workforce switched to remote work during the pandemic^28^. Studies looking specifically at employees who switched to remote work during the pandemic found that wake time was significantly delayed in these populations^21^. Because the average worker in the U.S. spends almost an hour commuting to work each day^29^, the switch to remote instead of onsite work likely supported the shift to later sleep timing by eliminating daily commutes. Another potential driver of the changes in sleep timing could be the imposition of strict curfews (in some regions) and the closing of restaurants, movie theaters, gyms, clubs, and other non-essential venues, thus forcing lifestyle changes that could impact sleep.

While both bed and wake times shifted later, the change was most pronounced in wake times near the beginning of the pandemic. We observed that whereas the most common weekday wake time prior to the pandemic was around 6:30-7am, the most common weekday wake time during the pandemic was 7–7:30am, suggesting that users were sleeping in later. Several studies have found that during the pandemic, this shift in sleep timing was observed primarily in people with evening chronotypes, who may have previously been waking earlier than their biological tendency because of pre-pandemic social or work obligations^1,3,30^. During the pandemic, the elimination of some of these obligations may have allowed people to adhere more closely to their biological sleep timing preference.

Prior to the pandemic, people tended to sleep earlier and less on work days and later and longer on free days (e.g. holidays, weekends), a phenomenon known as social jet lag^23^ which is associated with negative health and performance outcomes. Several studies found that social jetlag was reduced during the early phase of the pandemic. Korman et al. found that weekend sleep duration during the early phase of the pandemic actually decreased slightly compared to weekend sleep prior to the pandemic, while weekday sleep duration increased^17^. Similarly, several studies of smartphone users found that sleep onset and offset timings were delayed primarily on work days but not free days during the pandemic^22,24,31^. We also found that, from 2019 to 2020, larger changes in sleep timing and time in bed occurred on weekdays, suggesting a reduction in social jet lag. Future studies could explore this finding further with regard to geographic location and timing, to examine whether changes in social jetlag correspond with the time course of the pandemic. As lockdown policies have eased, we suspect that sleep duration and timing in 2021 may more closely resemble that prior to the pandemic as some studies have begun to report^20^.

### Limitations

We used primarily self-reported time in bed as a proxy for sleep duration, which may not necessarily reflect actual sleep duration, particularly as rates of insomnia have increased during the pandemic^16,32,33^. Additionally, the Sleep as Android application introduced a feature in 2019 that allowed users to manually confirm or reject probable sleep onset and offset times identified by the app. Since these probable times depend in part on user activity and light readings, they may not be as accurate as data derived from true self-report. However, unlike many other studies that asked participants to retroactively report sleep times from before the pandemic, our data was collected contemporaneously in 2019 and 2020. Finally, while we do not have demographic information on the users of the Sleep as Android app, this analysis includes data from users in 57 countries with a minimum of 100 users per country per year, suggesting that our findings are likely generalizable to the larger population.

## Methods

### Self-reported sleep measures

Self-reported sleep data was collected using the Sleep as Android smartphone application (https://sleep.urbandroid.org/), which was developed for the Android operating system as a smart alarm clock that awakens the user according to his/her sleep cycle. There were two ways to indicate sleep timings in the app: 1) users could indicate the start and end of the sleep episode by pressing the start/stop sleep tracking button in the app, or 2) beginning in early 2019, an additional feature was introduced where the app would identify probable sleep onset and offset times (based on past sleep habits, activity, and light readings) if the user failed to manually start and stop the sleep episode, and the user could then manually confirm or reject.

Only data from users who agreed to share information were retrieved. Variables available in the dataset including the date of the sleep records, sleep duration (calculated by the difference between self-reported sleep onset time and awake time), sleep onset time, awake time, and time zone obtained from the smartphone’s system.

### Analysis

The full dataset consisted of records retrieved from 64,858 individual users in 230 countries from January 2019 through December 2020. Because we used UTC time zone for inclusion criteria, we removed the first and last days of each year so that all dates included would be full days for all countries. We also excluded February 29th, 2020 because there was no comparable date in 2019. Similar to a previous report using data from Sleep as Android, the system time zone, instead of geographical coordinates, was used to classify user location (Anýž et al. 2019).

For analysis of global and geographic trends, data were first condensed by calculating the median bedtime and waketime for each day in each country. Countries with fewer than 100 users in either 2019 or 2020 were excluded from analysis (173 countries, 2,589 individuals in 2019 and 2,011 individuals in 2020 excluded) leaving a total of 57 countries in the final global dataset (71,405 users in 2019 and 56,146 users in 2020). Subsequent statistical analysis was performed on these median values. In addition to the global dataset, we selected twenty representative countries, each with a minimum of 200 users per year, for closer analysis of the effect of the pandemic on sleep across different geographic regions. These 20 countries represent a total of 15,873,653 sleep records collected from 53,545 users. Finally, we analyzed data from 12,551 users who had sleep data from both 2019 and 2020 to examine how changes in sleep duration were distributed.

Time in bed (TIB) and Midpoint of sleep (MOS) were calculated from the median bed and wake times. Statistical analyses for the data were performed using SAS version 9.4 (SAS Institute, Cary, NC). We tested two thresholds for inclusion in the global analysis: minimum 100 users and 150 users per country per year. With 150 users, all outcomes were approximately normally distributed. With 100 users, TIB, WT, and log-transformed MOS were approximately normally distributed but not BT. We ran all linear mixed models using both cutoffs and found no difference in the results so chose to report the results from the 100 users cutoff in order to include as much data as possible. In the subset of data from the 20 representative countries, all outcomes were approximately normally distributed so no transformations were performed for this analysis.

Linear mixed models followed by differences of least square means post-hoc tests were used to analyze the outcomes. We ran three models to analyze (1) global sleep changes, (2) sleep changes in the 20 representative countries, and (3) sleep changes in the United States. Model 1 was run on the full global dataset of 57 countries and included country as a random effect while year, quarter, and day of week were included as fixed effects. Model 2 was run on the subset of data from 20 representative countries and included country, year, and quarter as fixed effects. Finally, Model 3 was run on the subset of data from 20 representative countries and included year and month as fixed effects. For figures reporting global trends, we first averaged daily median values within each country and then averaged across countries. All *p* values reported in the text are Bonferroni-adjusted values; full statistical details are included in supplemental table S2.

### Study approval

This study used anonymous data collected through the Sleep as Android smartphone application. App users were informed of this anonymous data collection in the Sleep as Android Privacy Policy and were able to opt out of data collection in the application settings. Because data collection was completely anonymized and there is no way to link the data collected to any individual user, this study is exempt from IRB review under federal regulations (45 CFR 46.102(f) of the Health and Human Services Policy for Protection of Human Research Subjects).

## Supplementary Information

Supplementary Information.

## Data Availability

The datasets used in this study are available upon reasonable request.

## References

[1] Marelli S (2021). Impact of COVID-19 lockdown on sleep quality in university students and administration staff. J. Neurol..

[2] Wright KP (2020). Sleep in university students prior to and during COVID-19 Stay-at-Home orders. Curr. Biol..

[3] Leone MJ, Sigman M, Golombek DA (2020). Effects of lockdown on human sleep and chronotype during the COVID-19 pandemic. Curr. Biol..

[4] Robillard R (2021). Profiles of sleep changes during the COVID-19 pandemic: Demographic, behavioural and psychological factors. J. Sleep Res..

[5] Dey A (2021). COVID-19 pandemic lockdown-induced altered sleep/wake circadian rhythm, health complaints and stress among traffic police personnel in India. Chronobiol. Int..

[6] Herrero San Martin A (2020). Sleep characteristics in health workers exposed to the COVID-19 pandemic. Sleep Med..

[7] Jahrami H (2021). Sleep problems during the COVID-19 pandemic by population: a systematic review and meta-analysis. J. Clin. Sleep Med..

[8] Conroy DA (2021). The effects of COVID-19 stay-at-home order on sleep, health, and working patterns: A survey study of US health care workers. J. Clin. Sleep Med..

[9] Trakada A (2020). Sleep during "Lockdown" in the COVID-19 pandemic. Int. J. Environ. Res. Public Health.

[10] Pappa S (2020). Prevalence of depression, anxiety, and insomnia among healthcare workers during the COVID-19 pandemic: A systematic review and meta-analysis. Brain Behav. Immun..

[11] Zhang C (2020). Survey of insomnia and related social psychological factors among medical staff involved in the 2019 novel coronavirus disease outbreak. Front Psychiatry.

[12] Pinto J (2020). Sleep quality in times of Covid-19 pandemic. Sleep Med..

[13] Martinez-de-Quel O (2021). Physical activity, dietary habits and sleep quality before and during COVID-19 lockdown: A longitudinal study. Appetite.

[14] Hetkamp M (2020). Sleep disturbances, fear, and generalized anxiety during the COVID-19 shut down phase in Germany: Relation to infection rates, deaths, and German stock index DAX. Sleep Med..

[15] Gao C, Scullin MK (2020). Sleep health early in the coronavirus disease 2019 (COVID-19) outbreak in the United States: Integrating longitudinal, cross-sectional, and retrospective recall data. Sleep Med..

[16] Zitting KM (2021). Google Trends reveals increases in internet searches for insomnia during the 2019 coronavirus disease (COVID-19) global pandemic. J. Clin. Sleep Med..

[17] Korman M (2020). COVID-19-mandated social restrictions unveil the impact of social time pressure on sleep and body clock. Sci. Rep..

[18] Cellini N (2020). Changes in sleep pattern, sense of time and digital media use during COVID-19 lockdown in Italy. J. Sleep Res..

[19] Team AR (2021). Owls and larks do not exist: COVID-19 quarantine sleep habits. Sleep Med.

[20] Massar SAA (2021). Reopening after lockdown: The influence of working-from-home and digital device use on sleep, physical activity, and wellbeing following COVID-19 lockdown and reopening. Sleep.

[21] Barone Gibbs B (2021). Covid-19 shelter-at-home and work, lifestyle and well-being in desk workers. Occup. Med. (Lond.).

[22] Lee PH, Marek J, Nalevka P (2021). Sleep pattern in the US and 16 European countries during the COVID-19 outbreak using crowdsourced smartphone data. Eur J Public Health.

[23] Wittmann M (2006). Social jetlag: Misalignment of biological and social time. Chronobiol. Int..

[24] Lee PH, Marek J, Nalevka P (2020). Crowdsourced smartphone data reveal altered sleep/wake pattern in quarantined Chinese during the COVID-19 outbreak. Chronobiol. Int..

[25] Robbins R (2021). Correction: Estimated sleep duration before and during the COVID-19 pandemic in major metropolitan areas on different continents: Observational study of smartphone app data. J. Med. Internet Res..

[26] Kocevska D (2020). Sleep quality during the COVID-19 pandemic: Not one size fits all. Sleep Med..

[27] Salfi F (2021). Sleeping under the waves: A longitudinal study across the contagion peaks of the COVID-19 pandemic in Italy. J. Sleep Res..

[28] Parker, K., Horowitz, J. M. & Minkin, R. *How the Coronavirus Outbreak Has—and Hasn’t—Changed the Way Americans Work* (Pew Research Center, 2020).

[29] Burd, C., M. Burrows, R. & McKenzie, B. *Travel Time to Work in the United States: 2019*. 1–11 (.S. Census Bureau, 2021).

[30] Pepin JL (2021). Greatest changes in objective sleep architecture during COVID-19 lockdown in night owls with increased REM sleep. Sleep.

[31] Tahara Y (2021). Changes in sleep phase and body weight of mobile health App users during COVID-19 mild lockdown in Japan. Int. J. Obes. (Lond.).

[32] Li Y (2020). Insomnia and psychological reactions during the COVID-19 outbreak in China. J. Clin. Sleep Med..

[33] Lin YH, Chiang TW, Lin YL (2020). Increased internet searches for insomnia as an indicator of global mental health during the COVID-19 pandemic: Multinational longitudinal study. J Med Internet Res.

