# Supplementary material for: Increased sleep duration and delayed sleep timing during the COVID-19 pandemic
Source: Sci Rep. 2022 Jun 29;12:10937. doi: 10.1038/s41598-022-14782-x (PMC9243067; doi:10.1038/s41598-022-14782-x)
Supplement: Supplementary file 1 — Supplementary Information. [file 41598_2022_14782_MOESM1_ESM.pdf]

Increased sleep duration and delayed sleep timing during the COVID-19 pandemic

Authors: Robin K Yuan[1*], Kirsi-Marja Zitting[1], Liyaan Maskati[2], Jeff Huang[2]

Affiliations:

[1]Division of Sleep and Circadian Disorders, Department of Medicine, Brigham and Women's Hospital, and Division of Sleep Medicine, Harvard Medical School. 221 Longwood Avenue, Boston, MA 02115, USA

[2] Department of Computer Science, Brown University, 115 Waterman Street, Providence RI 02912, USA

Corresponding author:

Robin K. Yuan

221 Longwood Ave.

RF 386D

Boston, MA 02115

ryuan@bwh.harvard.edu

Supplemental Table S1. Monthly average waketimes (WT) and bedtimes (BT) for all 57 countries included in analysis. Monthly averages are derived from averaging the daily median waketimes and bedtimes in that month for each country.

			2019		2020	
Country	Month	Outcome	Mean Clock time	Standard Deviation	Mean Clock time	Standard Deviation
Argentina	1	WT	8:38	0:49	8:25	0:39
		BT	1:11	0:30	1:02	0:29
	2	WT	8:31	0:48	8:43	0:54
		BT	0:57	0:30	1:11	0:29
	3	WT	8:39	1:03	8:35	0:49
		BT	0:59	0:37	1:15	0:29
	4	WT	8:31	0:54	8:53	0:37
		BT	0:56	0:32	1:35	0:23
	5	WT	8:31	0:54	8:58	0:40
		BT	0:58	0:31	1:27	0:24
	6	WT	8:47	1:00	9:00	0:37
		BT	1:12	0:39	1:41	0:27
	7	WT	8:49	0:45	9:09	0:37
		BT	1:07	0:29	1:43	0:28
	8	WT	8:32	0:43	9:08	0:37
		BT	1:07	0:30	1:41	0:26
	9	WT	8:41	0:46	9:00	0:36
		BT	1:02	0:29	1:31	0:21
	10	WT	8:26	0:47	8:54	0:33
		BT	0:48	0:24	1:32	0:23
	11	WT	8:20	0:46	9:06	0:41
		BT	0:51	0:24	1:34	0:24
	12	WT	8:36	0:49	9:04	0:41
		BT	1:15	0:33	1:40	0:24
	Users		302		246	
Australia	1	WT	7:32	0:36	7:33	0:32
		BT	23:57	0:19	23:59	0:18
	2	WT	7:22	0:37	7:28	0:34
		BT	23:46	0:17	23:53	0:19
	3	WT	7:27	0:38	7:39	0:35
		BT	23:44	0:16	0:02	0:18
	4	WT	7:30	0:35	7:54	0:29
		BT	23:44	0:18	0:09	0:18
	5	WT	7:22	0:32	7:53	0:30
		BT	23:45	0:18	0:05	0:16
	6	WT	7:38	0:39	7:48	0:28
		BT	23:54	0:20	0:01	0:15
	7	WT	7:31	0:33	7:48	0:28
		BT	23:48	0:17	0:02	0:16
	8	WT	7:29	0:34	7:47	0:30
		BT	23:44	0:17	0:01	0:15
	9	WT	7:24	0:31	7:37	0:27
		BT	23:44	0:17	23:58	0:15
	10	WT	7:23	0:32	7:40	0:28
		BT	23:46	0:15	0:01	0:15
	11	WT	7:24	0:34	7:33	0:29
		BT	23:48	0:17	23:57	0:14
	12	WT	7:35	0:35	7:39	0:29
		BT	23:56	0:19	0:00	0:16
	Users		1730		1551	

Austria	1	WT	7:36	0:51	7:42	0:49
		BT	0:20	0:27	0:24	0:26
	2	WT	7:31	0:44	7:34	0:35
		BT	0:06	0:20	0:11	0:15
	3	WT	7:26	0:50	7:50	0:43
		BT	0:02	0:24	0:23	0:22
	4	WT	7:37	0:47	8:07	0:32
		BT	0:14	0:22	0:38	0:16
	5	WT	7:35	0:49	8:00	0:36
		BT	0:16	0:24	0:38	0:17
	6	WT	7:44	0:53	7:48	0:40
		BT	0:22	0:23	0:27	0:21
	7	WT	7:38	0:44	7:41	0:35
		BT	0:20	0:20	0:19	0:20
	8	WT	7:37	0:35	7:29	0:29
		BT	0:13	0:22	0:29	0:18
	9	WT	7:32	0:43	7:25	0:32
		BT	0:12	0:20	0:26	0:23
	10	WT	7:30	0:43	7:44	0:40
		BT	0:14	0:29	0:20	0:26
	11	WT	7:44	0:51	7:54	0:40
		BT	0:25	0:26	0:25	0:19
	12	WT	7:55	0:48	8:12	0:41
		BT	0:30	0:23	0:41	0:20
	Users		1339		986	
Belarus	1	WT	8:11	0:34	8:11	0:48
		BT	1:07	0:27	0:49	0:28
	2	WT	8:08	0:34	8:12	0:46
		BT	0:49	0:27	0:44	0:24
	3	WT	7:49	0:32	7:55	0:42
		BT	0:51	0:17	0:37	0:18
	4	WT	7:50	0:30	8:12	0:33
		BT	0:41	0:21	0:49	0:20
	5	WT	7:57	0:30	8:16	0:38
		BT	0:45	0:20	1:01	0:21
	6	WT	8:02	0:35	8:23	0:29
		BT	0:49	0:20	0:49	0:17
	7	WT	8:09	0:34	8:22	0:30
		BT	0:53	0:22	0:47	0:16
	8	WT	7:58	0:29	8:08	0:31
		BT	0:35	0:22	0:36	0:18
	9	WT	7:35	0:40	8:05	0:27
		BT	0:28	0:31	0:38	0:23
	10	WT	7:47	0:31	8:15	0:31
		BT	0:31	0:22	0:43	0:17
	11	WT	8:03	0:43	8:20	0:37
		BT	0:40	0:20	0:46	0:26
	12	WT	8:10	0:43	8:37	0:39
		BT	0:44	0:20	1:08	0:24
	Users		198		126	

Belgium	1	WT	7:54	0:50	7:47	0:44
		BT	0:14	0:23	0:17	0:23
	2	WT	7:49	0:50	7:48	0:46
		BT	0:08	0:26	0:17	0:21
	3	WT	7:44	0:44	7:56	0:43
		BT	0:12	0:23	0:33	0:21
	4	WT	7:47	0:43	8:18	0:28
		BT	0:16	0:17	0:34	0:13
	5	WT	7:51	0:42	8:22	0:33
		BT	0:17	0:20	0:37	0:16
	6	WT	7:59	0:45	8:05	0:33
		BT	0:30	0:20	0:33	0:16
	7	WT	7:59	0:41	8:10	0:29
		BT	0:24	0:17	0:40	0:17
	8	WT	8:00	0:39	8:14	0:30
		BT	0:22	0:19	0:36	0:16
	9	WT	7:45	0:46	7:53	0:34
		BT	0:07	0:21	0:24	0:17
	10	WT	7:46	0:43	8:01	0:33
		BT	0:12	0:23	0:26	0:20
	11	WT	7:55	0:47	8:16	0:31
		BT	0:18	0:23	0:35	0:16
	12	WT	8:08	0:51	8:24	0:34
		BT	0:28	0:26	0:36	0:17
	Users		1268		835	
Brazil	1	WT	7:49	0:46	7:40	0:43
		BT	0:43	0:18	0:34	0:20
	2	WT	7:36	0:51	7:39	0:46
		BT	0:29	0:20	0:33	0:18
	3	WT	7:47	0:49	7:49	0:49
		BT	0:40	0:23	0:45	0:23
	4	WT	7:33	0:50	8:16	0:38
		BT	0:31	0:18	1:02	0:20
	5	WT	7:34	0:48	8:11	0:36
		BT	0:31	0:20	1:02	0:18
	6	WT	7:47	0:53	8:02	0:31
		BT	0:38	0:21	0:53	0:17
	7	WT	7:44	0:46	7:55	0:32
		BT	0:37	0:18	0:39	0:17
	8	WT	7:31	0:48	7:57	0:34
		BT	0:28	0:18	0:37	0:19
	9	WT	7:32	0:49	7:48	0:32
		BT	0:27	0:18	0:36	0:18
	10	WT	7:24	0:45	7:45	0:35
		BT	0:23	0:18	0:40	0:14
	11	WT	7:29	0:46	7:47	0:36
		BT	0:26	0:20	0:44	0:17
	12	WT	7:46	0:47	7:50	0:33
		BT	0:39	0:24	0:45	0:18
	Users		1930		1720	

Bulgaria	1	WT	8:29	0:50	8:37	0:56
		BT	1:05	0:27	1:16	0:28
	2	WT	8:14	0:39	8:18	0:58
		BT	0:46	0:33	1:16	0:36
	3	WT	8:14	0:46	8:22	0:37
		BT	0:48	0:30	1:06	0:24
	4	WT	8:13	0:42	8:58	0:30
		BT	1:02	0:32	1:28	0:24
	5	WT	8:25	0:51	8:58	0:42
		BT	0:46	0:28	1:31	0:29
	6	WT	8:12	0:42	8:43	0:25
		BT	1:04	0:24	1:27	0:25
	7	WT	8:18	0:33	8:25	0:38
		BT	0:49	0:23	1:31	0:28
	8	WT	8:40	0:30	8:43	0:32
		BT	1:13	0:20	1:24	0:35
	9	WT	8:42	0:50	8:42	0:28
		BT	1:03	0:31	1:13	0:22
	10	WT	8:26	0:54	8:21	0:43
		BT	1:02	0:26	0:58	0:30
	11	WT	8:30	0:55	8:29	0:41
		BT	0:58	0:27	1:07	0:24
	12	WT	8:40	1:06	9:01	0:38
		BT	1:13	0:37	1:36	0:36
	Users		193		134	
Canada	1	WT	7:43	0:43	7:46	0:43
		BT	0:12	0:22	0:13	0:24
	2	WT	7:39	0:39	7:44	0:40
		BT	0:08	0:21	0:10	0:21
	3	WT	7:45	0:42	7:58	0:40
		BT	0:06	0:19	0:22	0:18
	4	WT	7:41	0:40	8:14	0:28
		BT	0:07	0:21	0:34	0:17
	5	WT	7:34	0:41	8:11	0:31
		BT	0:06	0:21	0:29	0:15
	6	WT	7:41	0:40	8:04	0:28
		BT	0:12	0:18	0:26	0:14
	7	WT	7:43	0:37	8:02	0:26
		BT	0:14	0:18	0:23	0:14
	8	WT	7:45	0:36	8:01	0:26
		BT	0:12	0:20	0:24	0:16
	9	WT	7:40	0:41	7:51	0:29
		BT	0:08	0:23	0:23	0:17
	10	WT	7:43	0:39	7:59	0:31
		BT	0:11	0:22	0:18	0:19
	11	WT	7:44	0:40	7:56	0:30
		BT	0:11	0:23	0:17	0:18
	12	WT	8:01	0:41	8:13	0:34
		BT	0:23	0:23	0:31	0:18
	Users		2683		2235	

Chile	1	WT	8:10	0:57	8:01	0:51
		BT	1:26	0:34	1:04	0:39
	2	WT	8:24	1:02	8:10	0:55
		BT	1:34	0:23	1:10	0:27
	3	WT	8:10	1:12	8:26	0:49
		BT	1:09	0:50	1:25	0:37
	4	WT	7:47	0:50	8:42	0:36
		BT	0:58	0:39	1:37	0:29
	5	WT	8:07	1:01	8:55	0:47
		BT	1:23	0:41	1:33	0:30
	6	WT	8:16	0:59	8:46	0:42
		BT	1:08	0:33	1:41	0:34
	7	WT	8:07	1:00	8:45	0:39
		BT	0:59	0:31	1:35	0:27
	8	WT	8:08	0:56	8:48	0:43
		BT	1:07	0:34	1:19	0:36
	9	WT	8:20	1:04	8:52	0:54
		BT	1:20	0:34	1:30	0:32
	10	WT	8:09	0:55	8:49	0:50
		BT	1:14	0:29	1:22	0:25
	11	WT	8:06	0:45	8:39	0:45
		BT	1:13	0:30	1:15	0:30
	12	WT	8:18	0:58	9:01	0:57
		BT	1:19	0:29	1:32	0:37
	Users		203		164	
China	1	WT	8:17	0:39	8:27	0:37
		BT	1:04	0:20	1:10	0:16
	2	WT	8:35	0:43	8:54	0:29
		BT	1:07	0:22	1:22	0:14
	3	WT	8:07	0:43	8:30	0:26
		BT	0:51	0:14	1:11	0:16
	4	WT	8:05	0:37	8:30	0:23
		BT	0:56	0:17	1:15	0:12
	5	WT	8:04	0:33	8:18	0:22
		BT	0:54	0:17	1:07	0:15
	6	WT	8:12	0:36	8:14	0:30
		BT	0:57	0:17	1:02	0:15
	7	WT	8:08	0:30	8:17	0:25
		BT	0:54	0:13	0:56	0:14
	8	WT	8:15	0:32	8:19	0:24
		BT	0:52	0:12	1:09	0:11
	9	WT	8:03	0:36	8:12	0:29
		BT	0:52	0:16	1:07	0:14
	10	WT	8:05	0:36	8:13	0:32
		BT	0:52	0:15	1:05	0:16
	11	WT	8:05	0:35	8:14	0:34
		BT	0:56	0:18	1:06	0:18
	12	WT	8:20	0:37	8:29	0:30
		BT	1:01	0:18	1:06	0:15
	Users		678		334	

Colombia	1	WT	6:58	0:58	6:59	0:56
		BT	23:50	0:24	0:03	0:22
	2	WT	6:58	0:54	6:55	0:53
		BT	23:47	0:28	0:17	0:37
	3	WT	7:08	1:01	7:12	0:57
		BT	0:17	0:34	0:16	0:37
	4	WT	7:10	0:55	7:56	0:36
		BT	0:14	0:28	0:52	0:30
	5	WT	7:10	0:55	7:56	0:49
		BT	0:04	0:32	0:51	0:28
	6	WT	7:14	0:56	7:32	0:35
		BT	23:59	0:34	0:26	0:24
	7	WT	7:07	0:58	7:25	0:33
		BT	23:53	0:31	0:16	0:28
	8	WT	7:03	0:54	7:22	0:29
		BT	23:54	0:24	0:19	0:27
	9	WT	7:05	0:58	7:11	0:38
		BT	23:53	0:32	0:19	0:27
	10	WT	6:51	0:57	7:13	0:25
		BT	23:47	0:32	0:01	0:24
	11	WT	6:55	0:47	7:13	0:32
		BT	23:46	0:30	0:01	0:28
	12	WT	7:00	0:59	7:23	0:24
		BT	0:00	0:36	0:15	0:33
	Users		190		149	
Croatia	1	WT	7:29	1:01	7:58	0:51
		BT	0:10	0:33	0:23	0:23
	2	WT	7:25	1:03	7:47	0:49
		BT	23:57	0:38	0:26	0:27
	3	WT	7:26	0:53	8:04	0:51
		BT	23:58	0:30	0:38	0:29
	4	WT	7:31	0:53	9:14	0:31
		BT	0:09	0:33	1:23	0:19
	5	WT	7:37	0:51	8:36	0:42
		BT	0:22	0:38	0:56	0:27
	6	WT	7:50	0:44	8:23	0:27
		BT	0:11	0:26	0:30	0:16
	7	WT	8:18	0:22	8:25	0:22
		BT	0:25	0:15	0:29	0:18
	8	WT	8:14	0:20	8:25	0:22
		BT	0:23	0:21	0:24	0:16
	9	WT	7:44	0:34	8:21	0:38
		BT	0:04	0:21	0:41	0:24
	10	WT	7:44	0:45	8:38	0:47
		BT	0:18	0:31	0:58	0:26
	11	WT	7:46	0:53	8:44	0:46
		BT	0:25	0:27	1:06	0:23
	12	WT	8:09	1:11	8:53	0:38
		BT	0:31	0:30	1:01	0:20
	Users		423		204	

Czechia	1	WT	7:30	0:50	7:27	0:47
		BT	0:07	0:22	0:03	0:21
	2	WT	7:26	0:44	7:26	0:42
		BT	23:59	0:21	23:57	0:17
	3	WT	7:20	0:46	7:42	0:38
		BT	23:53	0:20	0:05	0:15
	4	WT	7:21	0:43	7:55	0:25
		BT	0:00	0:16	0:14	0:10
	5	WT	7:25	0:42	7:47	0:30
		BT	0:04	0:15	0:10	0:13
	6	WT	7:26	0:44	7:28	0:33
		BT	0:02	0:19	0:05	0:13
	7	WT	7:31	0:36	7:35	0:29
		BT	0:02	0:15	0:04	0:13
	8	WT	7:28	0:36	7:35	0:30
		BT	23:58	0:15	0:00	0:15
	9	WT	7:19	0:43	7:21	0:37
		BT	23:50	0:19	23:57	0:16
	10	WT	7:16	0:40	7:35	0:35
		BT	23:52	0:18	0:07	0:14
	11	WT	7:20	0:45	7:44	0:33
		BT	23:59	0:18	0:08	0:14
	12	WT	7:50	0:51	7:54	0:41
		BT	0:19	0:23	0:15	0:19
	Users		1660		1298	
Denmark	1	WT	8:25	4:24	7:37	0:52
		BT	0:11	0:37	0:11	0:26
	2	WT	7:38	0:54	7:35	0:53
		BT	0:06	0:30	0:05	0:27
	3	WT	7:31	0:48	7:47	0:43
		BT	23:55	0:31	0:11	0:23
	4	WT	7:39	0:46	7:57	0:34
		BT	0:06	0:26	0:24	0:19
	5	WT	7:32	0:45	7:53	0:41
		BT	0:15	0:24	0:24	0:24
	6	WT	7:37	0:47	7:41	0:44
		BT	0:22	0:25	0:23	0:21
	7	WT	7:44	0:33	7:58	0:30
		BT	0:21	0:19	0:27	0:16
	8	WT	7:25	0:44	7:38	0:41
		BT	0:02	0:24	0:22	0:25
	9	WT	7:23	0:47	7:29	0:40
		BT	23:55	0:26	0:06	0:23
	10	WT	7:22	0:46	7:45	0:38
		BT	23:58	0:30	0:14	0:22
	11	WT	7:28	0:51	7:51	0:42
		BT	23:59	0:28	0:14	0:24
	12	WT	7:56	0:56	8:16	0:41
		BT	0:26	0:37	0:31	0:26
	Users		744		481	

Egypt	1	WT	10:14	1:10	8:31	0:51
		BT	2:57	1:11	1:13	1:00
	2	WT	9:48	1:13	8:25	0:39
		BT	1:48	1:29	1:18	0:55
	3	WT	9:48	1:01	8:53	1:00
		BT	2:20	0:52	1:46	1:09
	4	WT	9:25	0:45	9:32	1:05
		BT	2:03	1:02	2:51	0:59
	5	WT	10:01	1:11	11:20	1:04
		BT	3:14	1:06	3:58	0:47
	6	WT	8:51	1:07	10:02	0:47
		BT	1:55	1:17	2:58	0:52
	7	WT	9:38	0:47	9:43	1:03
		BT	2:26	0:43	2:59	0:44
	8	WT	10:14	1:01	10:39	0:58
		BT	2:32	1:15	3:15	1:00
	9	WT	8:24	0:58	9:30	0:56
		BT	1:42	0:53	2:11	0:36
	10	WT	8:28	1:01	8:59	0:44
		BT	1:50	0:53	1:41	0:45
	11	WT	8:12	1:00	9:00	0:48
		BT	1:06	0:37	1:29	0:35
	12	WT	8:24	0:49	9:22	0:55
		BT	1:18	0:44	2:14	0:42
	Users		182		104	
Estonia	1	WT	8:10	0:49	8:05	0:54
		BT	0:30	0:40	0:40	0:32
	2	WT	8:22	0:46	8:05	0:54
		BT	0:34	0:32	0:37	0:30
	3	WT	8:13	0:44	8:05	0:40
		BT	0:17	0:31	0:31	0:22
	4	WT	8:02	0:50	8:20	0:42
		BT	0:20	0:34	0:35	0:24
	5	WT	8:03	0:47	8:18	0:43
		BT	0:24	0:27	0:43	0:27
	6	WT	8:07	0:46	8:13	0:52
		BT	0:53	0:28	0:43	0:30
	7	WT	8:19	0:42	8:30	0:38
		BT	0:49	0:24	0:54	0:31
	8	WT	8:14	0:40	8:25	0:41
		BT	0:46	0:25	0:37	0:29
	9	WT	7:51	0:48	7:54	0:48
		BT	0:28	0:22	0:01	0:27
	10	WT	8:00	0:47	8:00	0:51
		BT	0:36	0:28	0:14	0:28
	11	WT	8:06	0:56	7:59	1:00
		BT	0:44	0:32	0:27	0:28
	12	WT	8:30	1:05	8:50	1:03
		BT	1:07	0:36	0:54	0:40
	Users		216		134	

Finland	1	WT	7:49	0:57	8:03	0:58
		BT	0:06	0:30	0:22	0:29
	2	WT	7:46	0:55	8:02	0:54
		BT	23:57	0:25	0:21	0:27
	3	WT	7:44	0:54	8:08	0:48
		BT	23:53	0:28	0:22	0:22
	4	WT	7:50	0:52	8:26	0:42
		BT	0:10	0:27	0:37	0:22
	5	WT	7:53	0:56	8:28	0:42
		BT	0:14	0:29	0:41	0:20
	6	WT	8:10	0:52	8:31	0:42
		BT	0:25	0:28	0:51	0:25
	7	WT	8:19	0:37	8:38	0:31
		BT	0:30	0:21	0:51	0:17
	8	WT	7:55	0:52	8:20	0:42
		BT	0:11	0:26	0:37	0:23
	9	WT	7:51	0:53	8:06	0:41
		BT	0:03	0:30	0:25	0:22
	10	WT	7:51	0:52	8:09	0:43
		BT	0:07	0:30	0:28	0:26
	11	WT	8:00	0:57	8:17	0:45
		BT	0:11	0:35	0:35	0:22
	12	WT	8:27	1:05	8:29	0:50
		BT	0:31	0:34	0:45	0:25
	Users		823		655	
France	1	WT	7:56	0:53	7:53	0:52
		BT	0:22	0:24	0:16	0:21
	2	WT	7:53	0:47	7:53	0:46
		BT	0:13	0:20	0:15	0:18
	3	WT	7:50	0:49	8:09	0:46
		BT	0:10	0:20	0:25	0:18
	4	WT	7:56	0:46	8:39	0:31
		BT	0:19	0:20	0:50	0:13
	5	WT	8:02	0:50	8:33	0:36
		BT	0:26	0:22	0:45	0:17
	6	WT	8:02	0:47	8:14	0:34
		BT	0:27	0:20	0:34	0:16
	7	WT	8:04	0:38	8:16	0:31
		BT	0:25	0:16	0:33	0:14
	8	WT	8:18	0:32	8:19	0:26
		BT	0:27	0:14	0:34	0:14
	9	WT	7:52	0:46	7:55	0:38
		BT	0:11	0:20	0:15	0:18
	10	WT	7:50	0:44	8:04	0:39
		BT	0:13	0:19	0:19	0:19
	11	WT	7:57	0:52	8:12	0:44
		BT	0:18	0:20	0:23	0:16
	12	WT	8:10	0:52	8:20	0:40
		BT	0:24	0:24	0:32	0:21
	Users		3112		2269	

Germany	1	WT	7:42	0:59	7:41	0:57
		BT	0:17	0:32	0:13	0:31
	2	WT	7:35	0:51	7:38	0:52
		BT	0:06	0:28	0:08	0:27
	3	WT	7:38	0:54	7:46	0:50
		BT	0:04	0:27	0:12	0:24
	4	WT	7:39	0:50	8:08	0:39
		BT	0:10	0:25	0:29	0:20
	5	WT	7:36	0:53	8:05	0:43
		BT	0:12	0:27	0:29	0:22
	6	WT	7:47	0:53	7:56	0:42
		BT	0:24	0:26	0:27	0:21
	7	WT	7:38	0:46	7:51	0:38
		BT	0:15	0:22	0:20	0:19
	8	WT	7:42	0:46	7:50	0:39
		BT	0:10	0:24	0:19	0:22
	9	WT	7:34	0:50	7:38	0:42
		BT	0:04	0:27	0:06	0:21
	10	WT	7:39	0:50	7:50	0:43
		BT	0:08	0:27	0:15	0:24
	11	WT	7:36	0:53	7:50	0:47
		BT	0:08	0:27	0:15	0:23
	12	WT	8:01	1:01	8:15	0:48
		BT	0:26	0:32	0:33	0:26
	Users		6253		5141	
Greece	1	WT	9:05	0:41	8:44	0:37
		BT	1:35	0:21	1:17	0:23
	2	WT	9:04	0:45	8:38	0:43
		BT	1:33	0:29	1:03	0:25
	3	WT	9:25	0:42	9:09	0:53
		BT	1:42	0:26	1:42	0:36
	4	WT	9:05	0:37	9:34	0:32
		BT	1:19	0:22	2:11	0:23
	5	WT	8:49	0:32	9:12	0:34
		BT	1:17	0:21	1:51	0:27
	6	WT	8:46	0:29	8:47	0:34
		BT	1:10	0:18	1:27	0:17
	7	WT	8:51	0:23	8:57	0:33
		BT	1:14	0:19	1:37	0:26
	8	WT	8:45	0:21	8:51	0:26
		BT	1:03	0:16	1:16	0:28
	9	WT	8:15	0:31	8:47	0:28
		BT	0:45	0:19	1:27	0:24
	10	WT	8:05	0:31	9:04	0:35
		BT	0:56	0:22	1:31	0:24
	11	WT	8:27	0:41	9:25	0:35
		BT	0:56	0:18	1:48	0:26
	12	WT	8:41	0:47	9:43	0:27
		BT	1:08	0:24	2:16	0:27
	Users		644		294	

Hong Kong	1	WT	8:24	0:53	8:25	0:53
		BT	1:25	0:25	1:20	0:26
	2	WT	8:44	0:57	9:07	0:40
		BT	1:31	0:25	1:42	0:22
	3	WT	8:18	0:49	8:44	0:36
		BT	1:22	0:23	1:32	0:26
	4	WT	8:22	0:50	9:02	0:34
		BT	1:31	0:22	1:43	0:18
	5	WT	8:12	0:47	8:40	0:31
		BT	1:21	0:21	1:25	0:20
	6	WT	8:17	0:55	8:29	0:46
		BT	1:18	0:22	1:20	0:24
	7	WT	8:16	0:50	8:41	0:39
		BT	1:24	0:21	1:28	0:21
	8	WT	8:25	0:49	8:47	0:42
		BT	1:27	0:20	1:44	0:15
	9	WT	8:12	0:52	8:24	0:40
		BT	1:19	0:26	1:38	0:20
	10	WT	8:16	0:58	8:29	0:46
		BT	1:23	0:23	1:34	0:19
	11	WT	8:12	0:50	8:29	0:45
		BT	1:18	0:22	1:31	0:19
	12	WT	8:23	0:52	8:50	0:38
		BT	1:20	0:26	1:38	0:17
	Users		409		248	
Hungary	1	WT	7:23	0:44	7:28	0:47
		BT	0:00	0:16	0:01	0:19
	2	WT	7:20	0:42	7:20	0:41
		BT	0:01	0:16	23:53	0:15
	3	WT	7:20	0:44	7:35	0:41
		BT	23:58	0:15	0:03	0:20
	4	WT	7:21	0:40	8:00	0:32
		BT	0:05	0:21	0:23	0:14
	5	WT	7:23	0:43	7:59	0:31
		BT	0:04	0:18	0:25	0:15
	6	WT	7:32	0:46	7:47	0:31
		BT	0:12	0:17	0:22	0:16
	7	WT	7:28	0:38	7:44	0:33
		BT	0:05	0:16	0:14	0:14
	8	WT	7:36	0:38	7:50	0:29
		BT	0:04	0:15	0:17	0:16
	9	WT	7:20	0:40	7:38	0:35
		BT	23:49	0:17	0:13	0:17
	10	WT	7:25	0:39	7:48	0:35
		BT	23:52	0:16	0:12	0:18
	11	WT	7:27	0:46	7:50	0:38
		BT	23:58	0:19	0:17	0:16
	12	WT	7:47	0:50	8:07	0:38
		BT	0:15	0:23	0:27	0:18
	Users		813		592	

India	1	WT	7:52	0:17	7:51	0:19
		BT	0:37	0:12	0:38	0:09
	2	WT	7:43	0:21	7:47	0:23
		BT	0:35	0:13	0:39	0:15
	3	WT	7:47	0:17	8:05	0:29
		BT	0:43	0:13	1:03	0:23
	4	WT	7:41	0:19	8:30	0:17
		BT	0:52	0:13	1:29	0:13
	5	WT	7:38	0:22	8:26	0:16
		BT	0:56	0:14	1:26	0:14
	6	WT	7:41	0:18	8:19	0:14
		BT	0:46	0:13	1:14	0:12
	7	WT	7:37	0:20	8:28	0:14
		BT	0:42	0:11	1:12	0:13
	8	WT	7:39	0:19	8:24	0:18
		BT	0:46	0:12	1:09	0:15
	9	WT	7:42	0:21	8:22	0:18
		BT	0:42	0:13	0:57	0:11
	10	WT	7:42	0:20	8:14	0:17
		BT	0:42	0:15	0:52	0:16
	11	WT	7:44	0:21	8:14	0:20
		BT	0:40	0:18	0:51	0:09
	12	WT	7:53	0:22	8:04	0:13
		BT	0:42	0:17	0:45	0:10
	Users		896		732	
Indonesia	1	WT	6:56	0:42	6:41	0:28
		BT	0:24	0:28	0:12	0:21
	2	WT	6:46	0:30	6:39	0:29
		BT	0:25	0:31	0:29	0:26
	3	WT	6:55	0:39	6:55	0:27
		BT	0:07	0:34	0:45	0:24
	4	WT	6:47	0:25	7:23	0:35
		BT	0:13	0:28	1:12	0:32
	5	WT	6:31	0:41	7:19	0:25
		BT	0:32	0:33	1:07	0:26
	6	WT	7:14	0:36	7:06	0:29
		BT	0:23	0:28	0:49	0:26
	7	WT	7:11	0:40	6:49	0:24
		BT	0:14	0:25	0:32	0:26
	8	WT	6:50	0:31	7:04	0:34
		BT	0:12	0:28	0:43	0:33
	9	WT	6:47	0:39	7:02	0:25
		BT	0:04	0:31	0:46	0:23
	10	WT	6:36	0:26	6:52	0:31
		BT	0:09	0:25	0:42	0:32
	11	WT	6:46	0:31	7:08	0:25
		BT	0:02	0:25	0:33	0:26
	12	WT	6:44	0:24	7:13	0:28
		BT	0:06	0:24	0:45	0:28
	Users		348		205	

Ireland	1	WT	8:03	0:59	8:03	0:54
		BT	0:37	0:29	0:32	0:27
	2	WT	8:04	0:47	8:10	0:49
		BT	0:29	0:26	0:35	0:26
	3	WT	8:02	0:50	8:20	0:41
		BT	0:37	0:32	0:44	0:25
	4	WT	8:03	0:48	8:36	0:36
		BT	0:41	0:26	0:55	0:22
	5	WT	7:58	0:44	8:41	0:37
		BT	0:39	0:29	0:59	0:22
	6	WT	8:04	0:52	8:35	0:32
		BT	0:35	0:27	1:00	0:26
	7	WT	7:52	0:48	8:30	0:33
		BT	0:31	0:24	0:51	0:22
	8	WT	7:59	0:46	8:28	0:28
		BT	0:32	0:20	0:59	0:21
	9	WT	7:47	0:53	8:10	0:30
		BT	0:14	0:22	0:52	0:26
	10	WT	7:47	0:49	8:18	0:37
		BT	0:14	0:21	0:49	0:21
	11	WT	7:54	0:49	8:24	0:39
		BT	0:20	0:28	0:47	0:26
	12	WT	8:15	0:53	8:41	0:34
		BT	0:39	0:26	1:03	0:24
	Users		490		285	
Israel	1	WT	7:55	0:43	7:56	0:45
		BT	0:38	0:19	0:39	0:18
	2	WT	7:56	0:45	7:47	0:52
		BT	0:35	0:14	0:37	0:14
	3	WT	7:55	0:45	8:19	0:53
		BT	0:37	0:23	0:59	0:25
	4	WT	8:04	0:44	9:02	0:29
		BT	0:50	0:18	1:32	0:20
	5	WT	7:58	0:51	8:33	0:35
		BT	0:51	0:19	1:10	0:16
	6	WT	7:59	0:53	8:07	0:35
		BT	0:42	0:16	1:01	0:16
	7	WT	7:57	0:40	8:28	0:33
		BT	0:34	0:15	1:06	0:13
	8	WT	8:08	0:47	8:28	0:38
		BT	0:43	0:14	1:06	0:15
	9	WT	7:55	0:44	8:32	0:38
		BT	0:36	0:11	1:06	0:18
	10	WT	8:14	0:53	8:25	0:42
		BT	0:48	0:20	1:07	0:17
	11	WT	7:53	0:44	8:10	0:40
		BT	0:33	0:13	0:46	0:13
	12	WT	7:54	0:41	8:12	0:39
		BT	0:36	0:14	0:48	0:15
	Users		383		278	

Italy	1	WT	8:01	0:42	8:02	0:42
		BT	0:48	0:23	0:43	0:21
	2	WT	7:56	0:42	7:58	0:38
		BT	0:42	0:19	0:37	0:18
	3	WT	7:54	0:43	8:22	0:40
		BT	0:38	0:18	0:55	0:19
	4	WT	7:59	0:43	8:36	0:29
		BT	0:43	0:17	1:06	0:13
	5	WT	7:55	0:44	8:26	0:32
		BT	0:48	0:20	0:59	0:16
	6	WT	8:00	0:40	8:17	0:27
		BT	0:47	0:17	0:51	0:14
	7	WT	7:59	0:34	8:11	0:24
		BT	0:45	0:13	0:48	0:11
	8	WT	8:15	0:28	8:17	0:23
		BT	0:50	0:12	0:49	0:13
	9	WT	7:57	0:36	8:04	0:29
		BT	0:38	0:17	0:45	0:13
	10	WT	7:47	0:37	8:09	0:33
		BT	0:34	0:18	0:45	0:16
	11	WT	7:57	0:43	8:14	0:35
		BT	0:41	0:19	0:47	0:12
	12	WT	8:10	0:45	8:21	0:37
		BT	0:47	0:23	0:51	0:14
	Users		2312		1418	
Japan	1	WT	7:27	0:34	7:23	0:33
		BT	0:45	0:12	0:39	0:12
	2	WT	7:21	0:34	7:19	0:30
		BT	0:41	0:13	0:35	0:11
	3	WT	7:21	0:30	7:18	0:29
		BT	0:39	0:11	0:36	0:11
	4	WT	7:17	0:29	7:19	0:28
		BT	0:34	0:09	0:32	0:10
	5	WT	7:19	0:32	7:26	0:25
		BT	0:38	0:12	0:37	0:13
	6	WT	7:15	0:35	7:13	0:25
		BT	0:34	0:12	0:29	0:12
	7	WT	7:13	0:29	7:13	0:26
		BT	0:36	0:11	0:28	0:11
	8	WT	7:20	0:26	7:18	0:23
		BT	0:34	0:10	0:31	0:11
	9	WT	7:17	0:31	7:14	0:24
		BT	0:36	0:12	0:31	0:11
	10	WT	7:16	0:30	7:13	0:23
		BT	0:36	0:11	0:27	0:09
	11	WT	7:18	0:31	7:17	0:25
		BT	0:34	0:11	0:31	0:10
	12	WT	7:19	0:31	7:17	0:25
		BT	0:39	0:12	0:33	0:10
	Users		2307		2499	

Latvia	1	WT	7:44	1:08	8:14	1:02
		BT	0:35	0:44	0:47	0:31
	2	WT	7:37	1:02	8:11	1:01
		BT	0:29	0:25	0:32	0:21
	3	WT	8:04	1:06	8:16	0:50
		BT	0:51	0:23	0:40	0:24
	4	WT	8:03	1:07	8:48	0:36
		BT	0:48	0:26	1:16	0:22
	5	WT	8:13	1:06	8:51	0:35
		BT	0:51	0:22	1:30	0:24
	6	WT	8:38	0:57	8:35	0:42
		BT	1:10	0:23	1:17	0:30
	7	WT	8:44	0:52	8:42	0:41
		BT	1:14	0:32	1:03	0:30
	8	WT	8:27	0:39	8:35	0:41
		BT	0:54	0:24	1:07	0:28
	9	WT	7:59	0:54	8:04	0:41
		BT	0:42	0:26	0:31	0:28
	10	WT	7:54	1:03	8:12	0:44
		BT	0:32	0:29	0:45	0:34
	11	WT	8:05	1:01	8:32	0:43
		BT	0:43	0:31	0:43	0:26
	12	WT	8:28	1:09	8:46	0:54
		BT	0:43	0:31	0:54	0:41
	Users		181		105	
Lithuania	1	WT	7:56	0:43	8:12	0:56
		BT	0:21	0:32	0:39	0:27
	2	WT	7:58	0:54	8:05	0:54
		BT	0:17	0:23	0:22	0:37
	3	WT	7:42	0:46	8:17	0:50
		BT	0:09	0:23	0:38	0:33
	4	WT	7:45	0:42	8:24	0:30
		BT	0:04	0:24	0:37	0:25
	5	WT	7:55	0:50	8:31	0:34
		BT	0:15	0:25	0:48	0:23
	6	WT	8:01	0:52	8:14	0:35
		BT	0:22	0:30	0:43	0:23
	7	WT	8:03	0:43	8:07	0:35
		BT	0:32	0:24	0:38	0:20
	8	WT	8:05	0:47	8:24	0:43
		BT	0:30	0:32	0:42	0:27
	9	WT	7:51	0:54	8:07	0:50
		BT	23:57	0:29	0:33	0:25
	10	WT	7:49	0:55	8:17	0:49
		BT	0:12	0:38	0:49	0:30
	11	WT	8:08	1:07	8:47	0:59
		BT	0:28	0:38	1:02	0:24
	12	WT	8:25	1:08	9:05	1:00
		BT	0:48	0:43	1:18	0:30
	Users		177		127	

Malaysia	1	WT	7:27	0:40	8:08	0:37
		BT	1:16	0:39	0:57	0:22
	2	WT	7:58	0:50	7:46	0:37
		BT	1:14	0:36	0:49	0:22
	3	WT	7:58	0:42	8:05	0:34
		BT	1:22	0:22	0:56	0:22
	4	WT	7:49	0:29	8:28	0:23
		BT	1:01	0:24	1:23	0:26
	5	WT	7:45	0:40	8:18	0:32
		BT	1:06	0:26	1:25	0:20
	6	WT	7:57	0:46	7:52	0:26
		BT	0:58	0:34	1:12	0:15
	7	WT	7:42	0:32	7:36	0:34
		BT	0:48	0:21	1:07	0:21
	8	WT	7:43	0:39	7:49	0:35
		BT	0:52	0:22	1:20	0:35
	9	WT	7:47	0:39	7:55	0:36
		BT	0:44	0:18	1:05	0:32
	10	WT	7:35	0:43	8:05	0:32
		BT	0:42	0:19	1:10	0:23
	11	WT	7:46	0:34	8:12	0:35
		BT	0:52	0:20	1:16	0:17
	12	WT	8:14	0:32	8:08	0:26
		BT	1:16	0:23	1:16	0:23
	Users		341		198	
Mexico	1	WT	7:41	0:45	7:28	0:40
		BT	0:40	0:24	0:26	0:23
	2	WT	7:31	0:46	7:22	0:45
		BT	0:31	0:24	0:24	0:22
	3	WT	7:31	0:42	7:30	0:41
		BT	0:24	0:22	0:23	0:19
	4	WT	7:41	0:43	8:11	0:34
		BT	0:40	0:19	1:05	0:22
	5	WT	7:32	0:40	8:16	0:33
		BT	0:40	0:20	1:02	0:19
	6	WT	7:36	0:43	8:08	0:35
		BT	0:35	0:20	0:58	0:18
	7	WT	7:42	0:38	8:04	0:29
		BT	0:38	0:22	0:50	0:16
	8	WT	7:32	0:40	8:11	0:34
		BT	0:37	0:20	0:53	0:21
	9	WT	7:27	0:49	8:00	0:30
		BT	0:40	0:25	0:43	0:24
	10	WT	7:17	0:43	7:53	0:23
		BT	0:29	0:27	0:36	0:17
	11	WT	7:26	0:42	7:50	0:26
		BT	0:32	0:22	0:33	0:20
	12	WT	7:42	0:41	8:01	0:32
		BT	0:41	0:30	0:32	0:15
	Users		857		720	

Netherlands	1	WT	7:55	0:50	7:53	0:49
		BT	0:15	0:24	0:11	0:25
	2	WT	7:47	0:47	7:50	0:46
		BT	0:05	0:24	0:08	0:25
	3	WT	7:47	0:46	7:58	0:42
		BT	0:04	0:22	0:11	0:21
	4	WT	7:47	0:43	8:11	0:33
		BT	0:09	0:21	0:22	0:17
	5	WT	7:49	0:45	8:13	0:36
		BT	0:15	0:23	0:24	0:18
	6	WT	7:58	0:48	8:01	0:39
		BT	0:23	0:22	0:23	0:19
	7	WT	7:54	0:40	8:05	0:33
		BT	0:21	0:19	0:24	0:16
	8	WT	8:00	0:40	8:06	0:34
		BT	0:19	0:21	0:24	0:19
	9	WT	7:46	0:46	7:51	0:35
		BT	0:07	0:24	0:17	0:19
	10	WT	7:46	0:42	8:03	0:33
		BT	0:07	0:25	0:16	0:20
	11	WT	7:50	0:47	7:59	0:36
		BT	0:08	0:25	0:14	0:22
	12	WT	8:10	0:53	8:16	0:39
		BT	0:20	0:28	0:28	0:22
	Users		2891		2241	
New Zealand	1	WT	7:36	0:29	7:33	0:32
		BT	0:04	0:24	23:58	0:17
	2	WT	7:34	0:32	7:28	0:30
		BT	0:03	0:22	23:48	0:16
	3	WT	7:38	0:41	7:39	0:36
		BT	23:52	0:20	23:56	0:21
	4	WT	7:36	0:39	8:05	0:34
		BT	23:50	0:23	0:15	0:21
	5	WT	7:23	0:37	7:48	0:28
		BT	23:53	0:25	0:03	0:15
	6	WT	7:31	0:39	7:41	0:32
		BT	23:47	0:24	23:56	0:24
	7	WT	7:30	0:37	7:33	0:30
		BT	23:50	0:19	23:53	0:17
	8	WT	7:27	0:35	7:31	0:28
		BT	23:47	0:23	0:12	0:19
	9	WT	7:19	0:35	7:27	0:30
		BT	23:41	0:21	23:54	0:19
	10	WT	7:26	0:37	7:31	0:28
		BT	23:49	0:27	23:52	0:24
	11	WT	7:16	0:32	7:30	0:32
		BT	23:35	0:21	23:53	0:21
	12	WT	7:29	0:32	7:48	0:33
		BT	0:02	0:22	0:00	0:20
	Users		381		289	

Norway	1	WT	8:04	1:00	8:02	1:07
		BT	0:34	0:33	0:35	0:38
	2	WT	8:02	1:02	7:57	1:01
		BT	0:29	0:30	0:28	0:35
	3	WT	7:58	1:04	8:12	0:59
		BT	0:21	0:35	0:36	0:27
	4	WT	8:13	0:59	8:30	0:54
		BT	0:36	0:31	0:53	0:30
	5	WT	8:00	0:57	8:21	0:46
		BT	0:30	0:32	0:45	0:26
	6	WT	8:15	1:04	8:15	0:50
		BT	0:51	0:33	0:51	0:26
	7	WT	8:36	0:40	8:46	0:37
		BT	1:00	0:23	1:01	0:21
	8	WT	8:05	0:54	8:20	0:48
		BT	0:38	0:31	0:44	0:26
	9	WT	7:56	1:00	8:10	0:54
		BT	0:28	0:36	0:28	0:26
	10	WT	7:59	0:59	8:17	0:54
		BT	0:33	0:33	0:39	0:31
	11	WT	8:05	1:03	8:28	0:54
		BT	0:38	0:29	0:51	0:30
	12	WT	8:31	1:08	8:49	0:57
		BT	0:59	0:38	1:12	0:29
	Users		695		527	
Philippines	1	WT	7:24	0:37	7:23	0:38
		BT	0:38	0:29	0:36	0:20
	2	WT	7:25	0:42	7:31	0:34
		BT	0:38	0:24	0:40	0:20
	3	WT	7:22	0:39	8:13	0:39
		BT	0:28	0:21	1:00	0:27
	4	WT	7:26	0:40	8:30	0:36
		BT	0:43	0:28	1:19	0:32
	5	WT	7:21	0:39	8:22	0:30
		BT	0:37	0:32	1:11	0:28
	6	WT	7:24	0:46	7:59	0:23
		BT	0:30	0:30	0:52	0:23
	7	WT	7:20	0:40	8:01	0:26
		BT	0:23	0:26	0:50	0:22
	8	WT	7:06	0:49	8:28	0:31
		BT	0:29	0:23	1:04	0:19
	9	WT	7:18	0:49	8:15	0:26
		BT	0:26	0:32	0:55	0:22
	10	WT	7:29	0:48	8:16	0:33
		BT	0:25	0:26	1:11	0:23
	11	WT	7:18	0:38	8:11	0:36
		BT	0:35	0:27	0:43	0:33
	12	WT	7:44	0:28	7:53	0:29
		BT	0:54	0:31	0:47	0:32
	Users		256		204	

Poland	1	WT	7:32	0:51	7:44	0:49
		BT	0:27	0:21	0:31	0:23
	2	WT	7:33	0:47	7:34	0:44
		BT	0:21	0:20	0:22	0:21
	3	WT	7:28	0:45	7:44	0:41
		BT	0:12	0:19	0:26	0:16
	4	WT	7:29	0:40	8:03	0:33
		BT	0:19	0:19	0:41	0:15
	5	WT	7:33	0:46	8:00	0:35
		BT	0:24	0:22	0:37	0:14
	6	WT	7:40	0:46	7:51	0:32
		BT	0:29	0:19	0:35	0:15
	7	WT	7:35	0:43	7:48	0:30
		BT	0:23	0:14	0:31	0:14
	8	WT	7:41	0:41	7:52	0:30
		BT	0:21	0:18	0:27	0:16
	9	WT	7:27	0:44	7:37	0:36
		BT	0:10	0:20	0:15	0:17
	10	WT	7:24	0:40	7:45	0:37
		BT	0:11	0:19	0:22	0:19
	11	WT	7:39	0:47	7:59	0:39
		BT	0:21	0:18	0:35	0:19
	12	WT	7:56	0:56	8:09	0:39
		BT	0:36	0:25	0:41	0:20
	Users		1857		1474	
Portugal	1	WT	8:34	0:49	8:28	0:51
		BT	1:15	0:21	1:10	0:29
	2	WT	8:23	0:53	8:32	0:52
		BT	1:12	0:29	1:11	0:30
	3	WT	8:20	0:49	8:53	0:59
		BT	1:14	0:31	1:26	0:34
	4	WT	8:32	0:47	9:07	0:45
		BT	1:16	0:27	1:42	0:27
	5	WT	8:23	0:48	9:05	0:43
		BT	1:08	0:26	1:41	0:26
	6	WT	8:36	0:50	8:52	0:35
		BT	1:15	0:27	1:29	0:28
	7	WT	8:28	0:47	8:39	0:33
		BT	1:11	0:29	1:15	0:18
	8	WT	8:39	0:36	8:46	0:27
		BT	1:16	0:18	1:12	0:14
	9	WT	8:23	0:46	8:33	0:32
		BT	1:10	0:26	1:03	0:24
	10	WT	8:17	0:50	8:31	0:35
		BT	1:03	0:20	0:59	0:21
	11	WT	8:30	0:55	8:33	0:34
		BT	1:13	0:27	1:03	0:20
	12	WT	8:49	1:00	8:52	0:37
		BT	1:30	0:34	1:16	0:23
	Users		770		500	

Puerto Rico	1	WT	7:47	0:49	7:45	1:00
		BT	0:37	0:46	0:46	0:52
	2	WT	7:50	0:49	7:45	0:47
		BT	0:22	0:45	0:35	0:33
	3	WT	8:06	0:45	8:02	0:54
		BT	0:45	0:46	0:54	0:55
	4	WT	7:53	0:58	8:16	0:45
		BT	0:29	0:47	0:55	0:45
	5	WT	7:46	0:46	7:54	0:45
		BT	0:18	0:40	0:25	0:37
	6	WT	7:44	0:39	7:52	0:31
		BT	0:09	0:34	0:03	0:39
	7	WT	7:32	0:42	7:49	0:37
		BT	0:18	0:34	0:14	0:33
	8	WT	7:31	0:49	7:55	0:32
		BT	0:15	0:34	0:13	0:39
	9	WT	7:47	0:53	8:03	0:31
		BT	0:44	0:26	0:08	0:31
	10	WT	7:27	0:39	7:36	0:27
		BT	0:16	0:43	0:02	0:32
	11	WT	7:27	0:42	7:42	0:34
		BT	23:54	0:34	0:13	0:45
	12	WT	7:45	0:43	8:02	0:30
		BT	0:31	0:43	0:20	0:37
	Users		159		121	
Romania	1	WT	8:11	0:52	8:28	1:00
		BT	0:53	0:27	1:10	0:31
	2	WT	7:54	0:47	8:02	0:47
		BT	0:37	0:22	0:49	0:24
	3	WT	8:02	0:51	8:30	0:52
		BT	0:34	0:21	1:00	0:23
	4	WT	8:18	0:53	8:59	0:36
		BT	0:50	0:19	1:25	0:21
	5	WT	8:09	0:51	8:45	0:39
		BT	0:45	0:22	1:13	0:25
	6	WT	8:16	0:49	8:41	0:34
		BT	0:45	0:19	1:17	0:21
	7	WT	8:08	0:40	8:23	0:31
		BT	0:46	0:17	1:01	0:15
	8	WT	8:16	0:40	8:29	0:35
		BT	0:50	0:17	0:59	0:17
	9	WT	8:09	0:53	8:20	0:33
		BT	0:36	0:15	0:48	0:16
	10	WT	7:56	0:50	8:15	0:37
		BT	0:36	0:24	0:53	0:18
	11	WT	8:04	0:49	8:20	0:37
		BT	0:42	0:17	0:52	0:21
	12	WT	8:33	1:01	8:44	0:40
		BT	1:10	0:33	1:09	0:18
	Users		425		267	

Russia	1	WT	8:31	1:07	8:37	1:05
		BT	1:14	0:30	1:10	0:27
	2	WT	8:06	0:50	8:13	0:47
		BT	0:53	0:19	0:51	0:17
	3	WT	8:08	0:48	8:19	0:42
		BT	0:48	0:17	0:59	0:17
	4	WT	7:57	0:42	8:52	0:25
		BT	0:41	0:14	1:17	0:11
	5	WT	8:12	0:44	8:55	0:28
		BT	0:52	0:16	1:16	0:11
	6	WT	8:19	0:43	8:41	0:25
		BT	0:53	0:15	1:13	0:10
	7	WT	8:15	0:38	8:32	0:27
		BT	0:52	0:13	1:04	0:12
	8	WT	8:12	0:38	8:32	0:27
		BT	0:45	0:12	1:01	0:10
	9	WT	7:59	0:45	8:16	0:30
		BT	0:40	0:16	0:49	0:12
	10	WT	8:02	0:45	8:26	0:33
		BT	0:45	0:16	0:58	0:14
	11	WT	8:14	0:50	8:45	0:36
		BT	0:53	0:17	1:13	0:15
	12	WT	8:17	0:52	8:42	0:36
		BT	1:01	0:19	1:14	0:16
	Users		2192		1832	
Singapore	1	WT	7:54	0:45	8:13	0:44
		BT	0:59	0:26	1:05	0:22
	2	WT	8:15	0:53	8:16	0:46
		BT	0:58	0:32	1:06	0:21
	3	WT	8:14	0:49	8:18	0:46
		BT	0:56	0:29	1:11	0:20
	4	WT	7:55	0:41	8:46	0:33
		BT	0:45	0:29	1:18	0:22
	5	WT	7:59	0:40	9:04	0:31
		BT	0:52	0:27	1:19	0:19
	6	WT	8:09	0:38	8:37	0:28
		BT	1:03	0:22	0:57	0:18
	7	WT	7:43	0:42	8:35	0:31
		BT	0:47	0:18	0:57	0:21
	8	WT	7:52	0:43	8:28	0:27
		BT	0:50	0:18	0:57	0:20
	9	WT	8:02	0:45	8:27	0:27
		BT	0:56	0:25	1:01	0:18
	10	WT	8:01	0:43	8:38	0:34
		BT	0:56	0:21	1:04	0:18
	11	WT	8:10	0:44	8:44	0:35
		BT	1:11	0:22	1:14	0:22
	12	WT	8:18	0:45	8:52	0:29
		BT	1:06	0:18	1:26	0:18
	Users		447		274	

Slovakia	1	WT	7:20	0:48	7:34	0:53
		BT	23:57	0:28	0:09	0:23
	2	WT	7:13	0:53	7:19	0:48
		BT	23:46	0:20	23:49	0:21
	3	WT	7:07	0:50	7:40	0:47
		BT	23:43	0:30	0:04	0:23
	4	WT	7:12	0:44	7:56	0:29
		BT	23:45	0:19	0:11	0:14
	5	WT	7:15	0:44	7:49	0:27
		BT	23:51	0:21	0:08	0:15
	6	WT	7:20	0:43	7:41	0:26
		BT	23:47	0:17	0:04	0:12
	7	WT	7:25	0:39	7:35	0:27
		BT	23:51	0:19	23:58	0:16
	8	WT	7:25	0:40	7:32	0:28
		BT	23:51	0:14	23:56	0:24
	9	WT	7:13	0:46	7:22	0:35
		BT	23:36	0:19	23:43	0:18
	10	WT	7:02	0:40	7:32	0:37
		BT	23:35	0:19	23:49	0:18
	11	WT	7:11	0:47	7:39	0:33
		BT	23:51	0:19	23:51	0:16
	12	WT	7:38	0:57	8:03	0:40
		BT	0:13	0:30	0:21	0:26
	Users		390		290	
Slovenia	1	WT	7:36	0:56	7:42	0:37
		BT	0:16	0:38	0:33	0:40
	2	WT	7:31	0:45	7:37	0:43
		BT	0:29	0:33	0:15	0:31
	3	WT	7:31	0:39	7:50	0:46
		BT	0:05	0:39	0:35	0:32
	4	WT	7:21	0:44	8:13	0:34
		BT	23:55	0:30	0:40	0:20
	5	WT	7:39	0:53	8:05	0:38
		BT	0:07	0:37	0:41	0:18
	6	WT	7:42	0:41	7:43	0:41
		BT	0:28	0:32	0:38	0:16
	7	WT	7:53	0:43	8:02	0:40
		BT	0:32	0:35	0:41	0:19
	8	WT	7:53	0:41	8:01	0:37
		BT	0:20	0:43	0:37	0:20
	9	WT	7:38	0:43	7:40	0:45
		BT	0:07	0:26	0:28	0:22
	10	WT	7:18	0:41	7:37	0:41
		BT	0:08	0:33	0:21	0:22
	11	WT	7:29	0:55	7:50	0:40
		BT	0:20	0:25	0:25	0:23
	12	WT	7:46	0:57	8:02	0:42
		BT	0:27	0:34	0:35	0:18
	Users		186		123	

South Africa	1	WT	6:44	0:44	6:52	0:37
		BT	23:29	0:25	23:27	0:20
	2	WT	6:36	0:39	6:39	0:40
		BT	23:20	0:19	23:18	0:16
	3	WT	6:48	0:40	6:57	0:48
		BT	23:20	0:23	23:25	0:27
	4	WT	6:47	0:46	7:34	0:31
		BT	23:20	0:19	23:59	0:14
	5	WT	6:49	0:43	7:30	0:33
		BT	23:22	0:23	23:56	0:17
	6	WT	6:57	0:42	7:34	0:37
		BT	23:20	0:18	23:52	0:20
	7	WT	6:46	0:43	7:25	0:37
		BT	23:18	0:15	23:48	0:17
	8	WT	6:52	0:44	7:26	0:40
		BT	23:18	0:21	23:47	0:26
	9	WT	6:50	0:42	7:02	0:33
		BT	23:23	0:18	23:46	0:25
	10	WT	6:34	0:37	7:10	0:32
		BT	23:13	0:18	23:37	0:18
	11	WT	6:45	0:44	7:07	0:35
		BT	23:14	0:20	23:36	0:16
	12	WT	7:12	0:39	7:21	0:34
		BT	23:30	0:25	23:48	0:19
	Users		265		213	
South Korea	1	WT	8:05	0:42	8:04	0:35
		BT	1:17	0:19	1:20	0:18
	2	WT	8:11	0:40	8:03	0:37
		BT	1:20	0:18	1:16	0:20
	3	WT	7:58	0:46	8:07	0:40
		BT	1:13	0:21	1:21	0:19
	4	WT	7:50	0:42	8:05	0:35
		BT	1:20	0:20	1:22	0:18
	5	WT	7:50	0:39	8:05	0:38
		BT	1:17	0:19	1:23	0:18
	6	WT	7:53	0:42	7:47	0:34
		BT	1:20	0:24	1:12	0:19
	7	WT	7:48	0:38	7:49	0:39
		BT	1:14	0:17	1:12	0:18
	8	WT	7:55	0:38	8:02	0:43
		BT	1:20	0:21	1:22	0:19
	9	WT	7:57	0:43	7:54	0:35
		BT	1:17	0:19	1:10	0:14
	10	WT	7:50	0:36	8:01	0:43
		BT	1:10	0:18	1:12	0:19
	11	WT	7:58	0:40	7:55	0:43
		BT	1:16	0:17	1:05	0:17
	12	WT	8:09	0:41	8:06	0:41
		BT	1:22	0:22	1:17	0:19
	Users		896		857	

Spain	1	WT	8:13	0:53	8:12	0:53
		BT	0:52	0:24	0:43	0:25
	2	WT	8:03	0:49	8:03	0:54
		BT	0:46	0:23	0:33	0:23
	3	WT	8:06	0:51	8:22	0:51
		BT	0:43	0:18	0:50	0:25
	4	WT	8:15	0:48	8:45	0:40
		BT	0:49	0:20	1:13	0:21
	5	WT	8:09	0:46	8:32	0:39
		BT	0:48	0:21	1:04	0:21
	6	WT	8:18	0:49	8:27	0:40
		BT	0:50	0:24	1:02	0:18
	7	WT	8:15	0:43	8:22	0:34
		BT	0:48	0:22	0:54	0:18
	8	WT	8:32	0:37	8:41	0:30
		BT	0:57	0:19	1:03	0:17
	9	WT	8:10	0:46	8:21	0:37
		BT	0:43	0:24	0:51	0:23
	10	WT	7:56	0:43	8:26	0:40
		BT	0:34	0:22	0:53	0:22
	11	WT	8:04	0:48	8:27	0:40
		BT	0:38	0:22	0:50	0:20
	12	WT	8:27	0:52	8:38	0:41
		BT	0:53	0:24	0:59	0:21
	Users		2162		1076	
Sweden	1	WT	7:38	0:55	7:44	0:58
		BT	0:04	0:32	0:08	0:34
	2	WT	7:31	0:53	7:33	0:51
		BT	23:52	0:29	23:52	0:28
	3	WT	7:31	0:51	7:36	0:44
		BT	23:49	0:28	23:56	0:24
	4	WT	7:33	0:50	7:46	0:40
		BT	23:52	0:28	0:04	0:24
	5	WT	7:33	0:52	7:48	0:45
		BT	23:56	0:30	0:12	0:24
	6	WT	7:50	0:53	7:49	0:40
		BT	0:14	0:27	0:15	0:24
	7	WT	8:08	0:32	8:19	0:23
		BT	0:19	0:18	0:31	0:12
	8	WT	7:46	0:45	7:49	0:38
		BT	0:05	0:25	0:13	0:24
	9	WT	7:29	0:51	7:32	0:38
		BT	23:51	0:29	0:02	0:23
	10	WT	7:29	0:48	7:42	0:40
		BT	23:51	0:29	0:07	0:26
	11	WT	7:35	0:54	7:51	0:42
		BT	23:58	0:31	0:09	0:23
	12	WT	8:01	1:01	8:13	0:47
		BT	0:18	0:36	0:29	0:27
	Users		1166		886	

Switzerland	1	WT	7:33	0:52	7:40	0:47
		BT	0:09	0:24	0:05	0:25
	2	WT	7:28	0:47	7:39	0:43
		BT	23:58	0:21	0:07	0:22
	3	WT	7:26	0:47	7:41	0:43
		BT	23:55	0:20	0:08	0:19
	4	WT	7:30	0:45	7:55	0:37
		BT	0:02	0:23	0:19	0:19
	5	WT	7:26	0:45	7:57	0:40
		BT	0:01	0:23	0:16	0:17
	6	WT	7:37	0:48	7:45	0:39
		BT	0:10	0:21	0:13	0:21
	7	WT	7:34	0:48	7:48	0:34
		BT	0:11	0:25	0:09	0:17
	8	WT	7:45	0:47	7:44	0:37
		BT	0:09	0:23	0:10	0:22
	9	WT	7:35	0:49	7:36	0:42
		BT	0:04	0:22	0:03	0:20
	10	WT	7:32	0:46	7:46	0:41
		BT	0:02	0:21	0:07	0:24
	11	WT	7:37	0:51	7:50	0:42
		BT	0:09	0:24	0:09	0:22
	12	WT	7:56	0:54	8:00	0:45
		BT	0:16	0:26	0:16	0:23
	Users		1016		779	
Taiwan	1	WT	8:06	0:41	8:12	0:33
		BT	1:01	0:16	1:07	0:15
	2	WT	8:18	0:41	8:17	0:32
		BT	1:03	0:17	1:05	0:14
	3	WT	8:10	0:36	8:04	0:35
		BT	1:01	0:18	1:03	0:15
	4	WT	8:06	0:33	8:17	0:37
		BT	1:04	0:13	1:11	0:16
	5	WT	8:05	0:31	8:11	0:36
		BT	1:04	0:17	1:06	0:14
	6	WT	8:14	0:39	8:03	0:28
		BT	1:06	0:14	1:02	0:13
	7	WT	8:05	0:25	8:03	0:31
		BT	1:01	0:13	1:03	0:11
	8	WT	8:07	0:31	8:10	0:30
		BT	1:12	0:17	1:05	0:14
	9	WT	8:04	0:33	7:57	0:32
		BT	1:02	0:17	0:55	0:16
	10	WT	8:02	0:30	8:15	0:39
		BT	1:05	0:16	1:08	0:15
	11	WT	8:06	0:31	8:10	0:36
		BT	1:09	0:12	1:11	0:14
	12	WT	8:02	0:32	8:11	0:34
		BT	1:04	0:14	1:10	0:15
	Users		511		411	

Thailand	1	WT	7:33	0:31	7:49	0:34
		BT	0:38	0:23	0:57	0:19
	2	WT	7:34	0:36	7:43	0:36
		BT	0:49	0:25	0:53	0:20
	3	WT	7:32	0:37	8:11	0:35
		BT	0:36	0:20	1:03	0:21
	4	WT	7:33	0:25	8:12	0:25
		BT	0:33	0:17	1:01	0:16
	5	WT	7:21	0:32	7:57	0:25
		BT	0:33	0:21	0:57	0:13
	6	WT	7:33	0:33	7:39	0:22
		BT	0:40	0:21	0:35	0:13
	7	WT	7:36	0:32	7:37	0:30
		BT	0:43	0:18	0:30	0:17
	8	WT	7:39	0:34	7:41	0:30
		BT	0:47	0:21	0:34	0:18
	9	WT	7:37	0:31	7:29	0:32
		BT	0:42	0:15	0:32	0:18
	10	WT	7:35	0:34	7:32	0:33
		BT	0:41	0:12	0:41	0:16
	11	WT	7:30	0:31	7:28	0:29
		BT	0:41	0:13	0:39	0:18
	12	WT	7:50	0:31	7:34	0:24
		BT	0:46	0:19	0:42	0:17
	Users		569		327	
Turkey	1	WT	8:04	0:40	8:23	0:43
		BT	1:10	0:25	1:20	0:23
	2	WT	8:05	0:53	8:17	0:51
		BT	1:04	0:25	1:20	0:21
	3	WT	8:05	0:44	8:37	0:50
		BT	1:07	0:21	1:47	0:29
	4	WT	7:58	0:48	9:09	0:40
		BT	1:01	0:21	2:24	0:28
	5	WT	8:04	0:47	9:21	0:40
		BT	1:34	0:32	2:35	0:29
	6	WT	8:22	0:40	8:39	0:35
		BT	1:27	0:26	1:39	0:20
	7	WT	8:03	0:35	8:45	0:27
		BT	1:22	0:19	1:28	0:29
	8	WT	8:20	0:39	8:45	0:36
		BT	1:24	0:19	1:32	0:21
	9	WT	7:54	0:39	8:21	0:36
		BT	0:54	0:21	1:25	0:27
	10	WT	7:43	0:39	8:35	0:35
		BT	0:47	0:14	1:24	0:17
	11	WT	8:01	0:45	8:41	0:37
		BT	1:09	0:23	1:22	0:22
	12	WT	8:05	0:40	8:57	0:45
		BT	1:27	0:24	1:45	0:30
	Users		543		348	

Ukraine	1	WT	8:25	0:46	8:23	0:39
		BT	1:07	0:22	0:55	0:22
	2	WT	8:16	0:42	8:09	0:36
		BT	0:52	0:19	0:43	0:16
	3	WT	8:19	0:38	8:29	0:35
		BT	0:49	0:15	0:51	0:18
	4	WT	8:15	0:34	8:47	0:19
		BT	0:57	0:14	1:02	0:11
	5	WT	8:15	0:33	8:39	0:22
		BT	1:08	0:17	1:07	0:16
	6	WT	8:12	0:38	8:22	0:24
		BT	0:53	0:14	0:54	0:10
	7	WT	8:14	0:31	8:24	0:23
		BT	0:49	0:10	0:54	0:13
	8	WT	8:17	0:37	8:27	0:27
		BT	0:48	0:14	0:53	0:14
	9	WT	8:06	0:39	8:22	0:32
		BT	0:38	0:13	0:50	0:16
	10	WT	8:05	0:38	8:26	0:27
		BT	0:39	0:17	0:55	0:18
	11	WT	8:07	0:39	8:33	0:36
		BT	0:38	0:13	0:52	0:18
	12	WT	8:24	0:39	8:41	0:33
		BT	0:56	0:19	1:05	0:19
	Users		530		430	
United Arab Emirates	1	WT	7:57	0:54	7:43	0:50
		BT	0:47	0:53	0:26	0:49
	2	WT	7:19	0:35	7:46	0:49
		BT	0:18	0:29	0:24	0:31
	3	WT	7:38	0:53	8:16	0:53
		BT	0:46	0:33	0:44	0:38
	4	WT	7:55	0:51	8:37	0:37
		BT	0:56	0:33	1:13	0:39
	5	WT	8:01	0:42	8:58	0:56
		BT	0:42	0:32	1:16	0:33
	6	WT	8:01	0:39	8:13	0:55
		BT	0:48	0:39	1:06	0:33
	7	WT	7:51	0:36	8:24	0:56
		BT	0:23	0:48	1:09	0:40
	8	WT	7:57	1:00	8:17	0:51
		BT	0:48	0:49	1:09	0:31
	9	WT	7:38	0:51	7:51	0:43
		BT	0:19	0:33	0:38	0:33
	10	WT	7:51	0:50	8:22	1:11
		BT	0:11	0:30	0:38	0:36
	11	WT	7:41	0:55	8:13	0:43
		BT	0:06	0:37	1:00	0:40
	12	WT	7:48	0:46	8:29	0:43
		BT	0:34	0:31	1:19	0:39
	Users		245		132	

United Kingdom	1	WT	7:47	0:41	7:44	0:42
		BT	0:13	0:21	0:11	0:19
	2	WT	7:44	0:40	7:42	0:38
		BT	0:06	0:18	0:11	0:19
	3	WT	7:41	0:40	7:49	0:37
		BT	0:02	0:18	0:13	0:16
	4	WT	7:45	0:35	8:16	0:26
		BT	0:07	0:17	0:28	0:14
	5	WT	7:42	0:36	8:17	0:26
		BT	0:09	0:18	0:31	0:13
	6	WT	7:44	0:40	8:11	0:26
		BT	0:11	0:18	0:26	0:15
	7	WT	7:38	0:35	8:08	0:25
		BT	0:06	0:16	0:24	0:14
	8	WT	7:47	0:33	8:05	0:24
		BT	0:08	0:17	0:22	0:15
	9	WT	7:38	0:37	7:46	0:25
		BT	0:00	0:17	0:16	0:15
	10	WT	7:40	0:36	7:58	0:29
		BT	0:04	0:19	0:16	0:16
	11	WT	7:44	0:39	8:05	0:32
		BT	0:08	0:18	0:22	0:16
	12	WT	8:01	0:42	8:15	0:31
		BT	0:20	0:20	0:31	0:16
	Users		4692		3622	
United States	1	WT	7:33	0:42	7:31	0:39
		BT	0:05	0:21	0:01	0:20
	2	WT	7:32	0:43	7:29	0:39
		BT	0:03	0:19	0:01	0:18
	3	WT	7:36	0:44	7:47	0:40
		BT	0:04	0:18	0:13	0:18
	4	WT	7:28	0:37	8:03	0:31
		BT	0:03	0:17	0:26	0:15
	5	WT	7:27	0:39	8:01	0:30
		BT	0:03	0:17	0:24	0:15
	6	WT	7:34	0:40	7:53	0:28
		BT	0:07	0:18	0:19	0:14
	7	WT	7:33	0:37	7:53	0:28
		BT	0:08	0:17	0:18	0:14
	8	WT	7:29	0:37	7:50	0:29
		BT	0:03	0:17	0:18	0:16
	9	WT	7:30	0:40	7:43	0:29
		BT	0:03	0:21	0:20	0:16
	10	WT	7:27	0:38	7:50	0:29
		BT	0:01	0:18	0:16	0:15
	11	WT	7:34	0:39	7:53	0:30
		BT	0:02	0:20	0:14	0:18
	12	WT	7:41	0:40	8:00	0:30
		BT	0:12	0:19	0:21	0:15
	Users		13582		12261	

Vietnam	1	WT	7:17	0:23	6:57	0:18
		BT	0:16	0:26	23:57	0:16
	2	WT	7:24	0:35	7:02	0:22
		BT	23:53	0:26	23:56	0:21
	3	WT	7:17	0:23	7:09	0:21
		BT	0:02	0:19	23:56	0:18
	4	WT	7:11	0:19	7:16	0:17
		BT	0:09	0:25	23:48	0:24
	5	WT	7:19	0:24	7:07	0:23
		BT	23:55	0:25	23:35	0:18
	6	WT	7:22	0:26	6:52	0:14
		BT	23:58	0:20	23:29	0:22
	7	WT	6:57	0:19	7:07	0:19
		BT	23:28	0:19	23:40	0:23
	8	WT	6:52	0:21	7:14	0:23
		BT	23:33	0:24	23:47	0:27
	9	WT	6:41	0:22	6:53	0:21
		BT	23:19	0:23	23:49	0:26
	10	WT	6:51	0:22	7:05	0:17
		BT	23:35	0:21	23:49	0:27
	11	WT	6:52	0:25	7:08	0:21
		BT	23:49	0:22	23:52	0:23
	12	WT	6:53	0:23	7:09	0:16
		BT	23:54	0:29	23:45	0:22
	Users		294		195	

Supplemental Table S2. Statistical details for all outcomes reported in text. Time in Bed (TIB), Midpoint of Sleep (MOS), Bedtime (BT), and Waketime (WT) are included.

Least Square Means									
Outcome	Effect	2019	2020	Estimate	Standard Error	Degrees of Freedom	T-value	P-value	Adjusted p-value
TIB	Year	-	-	-0.0804	0.004357	41000	-18.45	**<.0001**	-
logMOS	Year	-	-	-0.0581	0.0008	41000	-73.35	**<.0001**	-
BT	Year	-	-	-0.2001	0.003697	41000	-54.11	**<.0001**	-
WT	Year	-	-	-0.2805	0.004585	41000	-61.17	**<.0001**	-
TIB	Year*quarter	1	1	-0.01847	0.008783	41000	-2.1	0.0354	0.1416
TIB	Year*quarter	2	2	-0.1263	0.008705	41000	-14.51	<.0001	**<.0001**
TIB	Year*quarter	3	3	-0.04482	0.008661	41000	-5.18	<.0001	**<.0001**
TIB	Year*quarter	4	4	-0.132	0.008705	41000	-15.16	<.0001	**<.0001**
logMOS	Year*quarter	1	1	-0.02	0.001597	41000	-12.52	<.0001	**<.0001**
logMOS	Year*quarter	2	2	-0.09151	0.001583	41000	-57.81	<.0001	**<.0001**
logMOS	Year*quarter	3	3	-0.0551	0.001575	41000	-34.98	<.0001	**<.0001**
logMOS	Year*quarter	4	4	-0.06585	0.001583	41000	-41.59	<.0001	**<.0001**
BT	Year*quarter	1	1	-0.0635	0.0075	41000	-8.51	<.0001	**<.0001**
BT	Year*quarter	2	2	-0.3284	0.0074	41000	-44.45	<.0001	**<.0001**
BT	Year*quarter	3	3	-0.2034	0.0074	41000	-27.67	<.0001	**<.0001**
BT	Year*quarter	4	4	-0.2050	0.0074	41000	-27.75	<.0001	**<.0001**
WT	Year*quarter	1	1	-0.08192	0.009243	41000	-8.86	<.0001	**<.0001**
WT	Year*quarter	2	2	-0.4547	0.009162	41000	-49.63	<.0001	**<.0001**
WT	Year*quarter	3	3	-0.2482	0.009115	41000	-27.23	<.0001	**<.0001**
WT	Year*quarter	4	4	-0.337	0.009162	41000	-36.79	<.0001	**<.0001**
TIB	Year*day	Sun	Sun	0.0482	0.0115	41000	4.19	<.0001	**<.0001**
TIB	Year*day	Mon	Mon	-0.1434	0.0115	41000	-12.44	<.0001	**<.0001**
TIB	Year*day	Tue	Tue	-0.1418	0.0116	41000	-12.25	<.0001	**<.0001**
TIB	Year*day	Wed	Wed	-0.1131	0.0115	41000	-9.81	<.0001	**<.0001**
TIB	Year*day	Thu	Thu	-0.1191	0.0115	41000	-10.34	<.0001	**<.0001**
TIB	Year*day	Fri	Fri	-0.1219	0.0115	41000	-10.59	<.0001	**<.0001**
TIB	Year*day	Sat	Sat	0.0283	0.0115	41000	2.45	0.0141	0.0987
logMOS	Year*day	Sun	Sun	-0.01891	0.002094	41000	-9.03	<.0001	**<.0001**
logMOS	Year*day	Mon	Mon	-0.07026	0.002096	41000	-33.52	<.0001	**<.0001**
logMOS	Year*day	Tue	Tue	-0.07915	0.002105	41000	-37.6	<.0001	**<.0001**
logMOS	Year*day	Wed	Wed	-0.06788	0.002096	41000	-32.39	<.0001	**<.0001**
logMOS	Year*day	Thu	Thu	-0.07097	0.002094	41000	-33.89	<.0001	**<.0001**
logMOS	Year*day	Fri	Fri	-0.07004	0.002094	41000	-33.44	<.0001	**<.0001**
logMOS	Year*day	Sat	Sat	-0.02958	0.002094	41000	-14.13	<.0001	**<.0001**
BT	Year*day	Sun	Sun	-0.1193	0.009773	41000	-12.2	<.0001	**<.0001**
BT	Year*day	Mon	Mon	-0.2085	0.00978	41000	-21.32	<.0001	**<.0001**
BT	Year*day	Tue	Tue	-0.2443	0.009824	41000	-24.87	<.0001	**<.0001**
BT	Year*day	Wed	Wed	-0.2126	0.00978	41000	-21.74	<.0001	**<.0001**
BT	Year*day	Thu	Thu	-0.2257	0.009773	41000	-23.1	<.0001	**<.0001**
BT	Year*day	Fri	Fri	-0.2286	0.009773	41000	-23.39	<.0001	**<.0001**
BT	Year*day	Sat	Sat	-0.1615	0.009773	41000	-16.53	<.0001	**<.0001**
WT	Year*day	Sun	Sun	-0.07103	0.01212	41000	-5.86	<.0001	**<.0001**
WT	Year*day	Mon	Mon	-0.3519	0.01213	41000	-29.01	<.0001	**<.0001**
WT	Year*day	Tue	Tue	-0.3861	0.01218	41000	-31.69	<.0001	**<.0001**
WT	Year*day	Wed	Wed	-0.3257	0.01213	41000	-26.85	<.0001	**<.0001**
WT	Year*day	Thu	Thu	-0.3448	0.01212	41000	-28.45	<.0001	**<.0001**
WT	Year*day	Fri	Fri	-0.3506	0.01212	41000	-28.92	<.0001	**<.0001**
WT	Year*day	Sat	Sat	-0.1333	0.01212	41000	-11	<.0001	**<.0001**

Bonferroni adjustements were made for 4 comparisons in the Year*quarter analysis (each quarter of 2019 vs. the same quarter in 2020), and 7 comparisons in the Year*day analysis (each day of the week of 2019 vs. the same day of the week in 2020)

	Least Square Means									
Country	Outcome	Effect	2019	2020	Estimate	Standard Error	Degrees of Freedom	T-value	P-value	Adjusted p-value
Australia	TIB	Year*location	-	-	-0.01137	0.02898	14000	-0.39	0.6949	1.00
	MOS	Year*location	-	-	-0.2103	0.0362	14000	-5.80	<.0001	**<.0001**
	BT	Year*location	-	-	-0.2046	0.02547	14000	-8.03	<.0001	**<.0001**
	WT	Year*location	-	-	-0.216	0.04896	14000	-4.41	<.0001	**<.0001**
	TIB	Year*quarter*location	1	1	0.04558	0.05841	14000	0.78	0.4351	1.00
	TIB	Year*quarter*location	2	2	-0.07958	0.05792	14000	-1.37	0.1695	1.000
	TIB	Year*quarter*location	3	3	-0.01486	0.05761	14000	-0.26	0.7965	1.00
	TIB	Year*quarter*location	4	4	0.003388	0.05792	14000	0.06	0.9534	1.00
	MOS	Year*quarter*location	1	1	-0.1277	0.07302	14000	-1.75	0.0804	1.00
	MOS	Year*quarter*location	2	2	-0.3255	0.07242	14000	-4.49	<.0001	**<.0001**
	MOS	Year*quarter*location	3	3	-0.2512	0.07202	14000	-3.49	0.0005	**0.04**
	MOS	Year*quarter*location	4	4	-0.1368	0.07242	14000	-1.89	0.059	1.00
	BT	Year*quarter*location	1	1	-0.1505	0.0513	14000	-2.93	0.0034	0.27
	BT	Year*quarter*location	2	2	-0.2857	0.0509	14000	-5.61	<.0001	**<.0001**
	BT	Year*quarter*location	3	3	-0.2437	0.0506	14000	-4.81	<.0001	**<.0001**
	BT	Year*quarter*location	4	4	-0.1385	0.0509	14000	-2.72	0.0065	0.52
	WT	Year*quarter*location	1	1	-0.1049	0.09866	14000	-1.06	0.2878	1.00
	WT	Year*quarter*location	2	2	-0.3653	0.09785	14000	-3.73	0.0002	**0.02**
	WT	Year*quarter*location	3	3	-0.2586	0.09731	14000	-2.66	0.0079	0.63
	WT	Year*quarter*location	4	4	-0.1351	0.09785	14000	-1.38	0.1675	1.00
Brazil	TIB	Year*location	-	-	-0.06009	0.02898	14000	-2.07	0.0382	0.76
	MOS	Year*location	-	-	-0.2253	0.0362	14000	-6.22	<.0001	**<.0001**
	BT	Year*location	-	-	-0.1953	0.02547	14000	-7.67	<.0001	**<.0001**
	WT	Year*location	-	-	-0.2554	0.04896	14000	-5.22	<.0001	**<.0001**
	TIB	Year*quarter*location	1	1	0.02103	0.05841	14000	0.36	0.7187	1.00
	TIB	Year*quarter*location	2	2	-0.09707	0.05792	14000	-1.68	0.0938	1.000
	TIB	Year*quarter*location	3	3	-0.1791	0.05761	14000	-3.11	0.0019	0.15
	TIB	Year*quarter*location	4	4	0.01474	0.05792	14000	0.25	0.7991	1.00
	MOS	Year*quarter*location	1	1	0.01335	0.07302	14000	0.18	0.8549	1.00
	MOS	Year*quarter*location	2	2	-0.4851	0.07242	14000	-6.7	<.0001	**<.0001**
	MOS	Year*quarter*location	3	3	-0.2038	0.07202	14000	-2.83	0.0047	0.38
	MOS	Year*quarter*location	4	4	-0.2258	0.07242	14000	-3.12	0.0018	0.14
	BT	Year*quarter*location	1	1	0.0028	0.0513	14000	0.06	0.9559	1.00
	BT	Year*quarter*location	2	2	-0.4365	0.0509	14000	-8.58	<.0001	**<.0001**
	BT	Year*quarter*location	3	3	-0.1143	0.0506	14000	-2.26	0.0240	1.00
	BT	Year*quarter*location	4	4	-0.2332	0.0509	14000	-4.58	<.0001	**<.0001**
	WT	Year*quarter*location	1	1	0.02387	0.09866	14000	0.24	0.8088	1.00
	WT	Year*quarter*location	2	2	-0.5336	0.09785	14000	-5.45	<.0001	**<.0001**
	WT	Year*quarter*location	3	3	-0.2934	0.09731	14000	-3.01	0.0026	0.21
	WT	Year*quarter*location	4	4	-0.2184	0.09785	14000	-2.23	0.0256	1.00
Canada	TIB	Year*location	-	-	-0.0848	0.02898	14000	-2.93	0.0034	0.07
	MOS	Year*location	-	-	-0.2352	0.0362	14000	-6.49	<.0001	**<.0001**
	BT	Year*location	-	-	-0.1928	0.02547	14000	-7.57	<.0001	**<.0001**
	WT	Year*location	-	-	-0.2776	0.04896	14000	-5.67	<.0001	**<.0001**
	TIB	Year*quarter*location	1	1	-0.00148	0.05841	14000	-0.03	0.9798	1.00
	TIB	Year*quarter*location	2	2	-0.1573	0.05792	14000	-2.72	0.0066	0.528
	TIB	Year*quarter*location	3	3	-0.05815	0.05761	14000	-1.01	0.3128	1.00
	TIB	Year*quarter*location	4	4	-0.1223	0.05792	14000	-2.11	0.0348	1.00
	MOS	Year*quarter*location	1	1	-0.1164	0.07302	14000	-1.59	0.111	1.00
	MOS	Year*quarter*location	2	2	-0.4397	0.07242	14000	-6.07	<.0001	**<.0001**
	MOS	Year*quarter*location	3	3	-0.2295	0.07202	14000	-3.19	0.0014	0.11
	MOS	Year*quarter*location	4	4	-0.155	0.07242	14000	-2.14	0.0323	1.00
	BT	Year*quarter*location	1	1	-0.1157	0.0513	14000	-2.25	0.0243	1.00
	BT	Year*quarter*location	2	2	-0.3611	0.0509	14000	-7.09	<.0001	**<.0001**
	BT	Year*quarter*location	3	3	-0.2005	0.0506	14000	-3.96	<.0001	**<.0001**
	BT	Year*quarter*location	4	4	-0.0939	0.0509	14000	-1.84	0.0652	1.00
	WT	Year*quarter*location	1	1	-0.1171	0.09866	14000	-1.19	0.2352	1.00
	WT	Year*quarter*location	2	2	-0.5184	0.09785	14000	-5.3	<.0001	**<.0001**
	WT	Year*quarter*location	3	3	-0.2586	0.09731	14000	-2.66	0.0079	0.63
	WT	Year*quarter*location	4	4	-0.2161	0.09785	14000	-2.21	0.0272	1.00

China	TIB	Year*location	-	-	0.004547	0.02898	14000	0.16	0.8753	1.00
	MOS	Year*location	-	-	-0.1584	0.0362	14000	-4.37	<.0001	**<.0001**
	BT	Year*location	-	-	-0.1607	0.02547	14000	-6.31	<.0001	**<.0001**
	WT	Year*location	-	-	-0.1561	0.04896	14000	-3.19	0.0014	**0.03**
	TIB	Year*quarter*location	1	1	-0.06758	0.05841	14000	-1.16	0.2472	1.00
	TIB	Year*quarter*location	2	2	-0.02024	0.05792	14000	-0.35	0.7268	1.000
	TIB	Year*quarter*location	3	3	0.07808	0.05761	14000	1.36	0.1753	1.00
	TIB	Year*quarter*location	4	4	0.02793	0.05792	14000	0.48	0.6297	1.00
	MOS	Year*quarter*location	1	1	-0.2592	0.07302	14000	-3.55	0.0004	**0.03**
	MOS	Year*quarter*location	2	2	-0.2148	0.07242	14000	-2.97	0.003	0.24
	MOS	Year*quarter*location	3	3	-0.1149	0.07202	14000	-1.59	0.1108	1.00
	MOS	Year*quarter*location	4	4	-0.04473	0.07242	14000	-0.62	0.5368	1.00
	BT	Year*quarter*location	1	1	-0.2254	0.0513	14000	-4.39	<.0001	**<.0001**
	BT	Year*quarter*location	2	2	-0.2047	0.0509	14000	-4.02	<.0001	**<.0001**
	BT	Year*quarter*location	3	3	-0.1539	0.0506	14000	-3.04	0.0024	0.19
	BT	Year*quarter*location	4	4	-0.0587	0.0509	14000	-1.15	0.2489	1.00
	WT	Year*quarter*location	1	1	-0.293	0.09866	14000	-2.97	0.0030	0.24
	WT	Year*quarter*location	2	2	-0.2249	0.09785	14000	-2.3	0.0215	1.00
	WT	Year*quarter*location	3	3	-0.07582	0.09731	14000	-0.78	0.4359	1.00
	WT	Year*quarter*location	4	4	-0.03077	0.09785	14000	-0.31	0.7532	1.00
Finland	TIB	Year*location	-	-	0.05838	0.02898	14000	2.01	0.044	0.88
	MOS	Year*location	-	-	-0.3616	0.03623	14000	-9.98	<.0001	**<.0001**
	BT	Year*location	-	-	-0.3907	0.02547	14000	-15.34	<.0001	**<.0001**
	WT	Year*location	-	-	-0.3324	0.04896	14000	-6.79	<.0001	**<.0001**
	TIB	Year*quarter*location	1	1	0.07749	0.05841	14000	1.33	0.1846	1.00
	TIB	Year*quarter*location	2	2	-0.06209	0.05792	14000	-1.07	0.2838	1.000
	TIB	Year*quarter*location	3	3	0.06141	0.05761	14000	1.07	0.2864	1.00
	TIB	Year*quarter*location	4	4	0.1567	0.05792	14000	2.71	0.0068	0.54
	MOS	Year*quarter*location	1	1	-0.3441	0.07302	14000	-4.71	<.0001	**<.0001**
	MOS	Year*quarter*location	2	2	-0.4843	0.07242	14000	-6.69	<.0001	**<.0001**
	MOS	Year*quarter*location	3	3	-0.3589	0.07202	14000	-4.98	<.0001	**<.0001**
	MOS	Year*quarter*location	4	4	-0.2589	0.07242	14000	-3.58	0.0004	**0.03**
	BT	Year*quarter*location	1	1	-0.3828	0.0513	14000	-7.46	<.0001	**<.0001**
	BT	Year*quarter*location	2	2	-0.4533	0.0509	14000	-8.90	<.0001	**<.0001**
	BT	Year*quarter*location	3	3	-0.3896	0.0506	14000	-7.69	<.0001	**<.0001**
	BT	Year*quarter*location	4	4	-0.3373	0.0509	14000	-6.63	<.0001	**<.0001**
	WT	Year*quarter*location	1	1	-0.3053	0.09866	14000	-3.09	0.0020	0.16
	WT	Year*quarter*location	2	2	-0.5154	0.09785	14000	-5.27	<.0001	**<.0001**
	WT	Year*quarter*location	3	3	-0.3282	0.09731	14000	-3.37	0.0007	0.06
	WT	Year*quarter*location	4	4	-0.1806	0.09785	14000	-1.85	0.0650	1.00
France	TIB	Year*location	-	-	-0.07035	0.02898	14000	-2.43	0.0152	0.30
	MOS	Year*location	-	-	-0.1879	0.0362	14000	-5.18	<.0001	**<.0001**
	BT	Year*location	-	-	-0.1527	0.02547	14000	-5.99	<.0001	**<.0001**
	WT	Year*location	-	-	-0.223	0.04896	14000	-4.56	<.0001	**<.0001**
	TIB	Year*quarter*location	1	1	-0.02149	0.05841	14000	-0.37	0.7129	1.00
	TIB	Year*quarter*location	2	2	-0.1578	0.05792	14000	-2.72	0.0065	0.520
	TIB	Year*quarter*location	3	3	0.02627	0.05761	14000	0.46	0.6484	1.00
	TIB	Year*quarter*location	4	4	-0.1284	0.05792	14000	-2.22	0.0267	1.00
	MOS	Year*quarter*location	1	1	-0.08208	0.07302	14000	-1.12	0.261	1.00
	MOS	Year*quarter*location	2	2	-0.3967	0.07242	14000	-5.48	<.0001	**<.0001**
	MOS	Year*quarter*location	3	3	-0.09764	0.07202	14000	-1.36	0.1752	1.00
	MOS	Year*quarter*location	4	4	-0.1751	0.07242	14000	-2.42	0.0156	1.00
	BT	Year*quarter*location	1	1	-0.0713	0.0513	14000	-1.39	0.1647	1.00
	BT	Year*quarter*location	2	2	-0.3178	0.0509	14000	-6.24	<.0001	**<.0001**
	BT	Year*quarter*location	3	3	-0.1108	0.0506	14000	-2.19	0.0287	1.00
	BT	Year*quarter*location	4	4	-0.1109	0.0509	14000	-2.18	0.0294	1.00
	WT	Year*quarter*location	1	1	-0.09283	0.09866	14000	-0.94	0.3468	1.00
	WT	Year*quarter*location	2	2	-0.4755	0.09785	14000	-4.86	<.0001	**<.0001**
	WT	Year*quarter*location	3	3	-0.08451	0.09731	14000	-0.87	0.3852	1.00
	WT	Year*quarter*location	4	4	-0.2393	0.09785	14000	-2.45	0.0145	1.00

Germany	TIB	Year*location	-	-	-0.07574	0.02898	14000	-2.61	0.009	0.18
	MOS	Year*location	-	-	-0.1576	0.0362	14000	-4.35	<.0001	**<.0001**
	BT	Year*location	-	-	-0.1197	0.02547	14000	-4.7	<.0001	**<.0001**
	WT	Year*location	-	-	-0.1955	0.04896	14000	-3.99	<.0001	**<.0001**
	TIB	Year*quarter*location	1	1	-0.0121	0.05841	14000	-0.21	0.8359	1.00
	TIB	Year*quarter*location	2	2	-0.1669	0.05792	14000	-2.88	0.004	0.320
	TIB	Year*quarter*location	3	3	-0.04746	0.05761	14000	-0.82	0.41	1.00
	TIB	Year*quarter*location	4	4	-0.07647	0.05792	14000	-1.32	0.1868	1.00
	MOS	Year*quarter*location	1	1	-0.0433	0.07302	14000	-0.59	0.5532	1.00
	MOS	Year*quarter*location	2	2	-0.2939	0.07242	14000	-4.06	<.0001	**<.0001**
	MOS	Year*quarter*location	3	3	-0.1195	0.07202	14000	-1.66	0.0972	1.00
	MOS	Year*quarter*location	4	4	-0.1738	0.07242	14000	-2.4	0.0164	1.00
	BT	Year*quarter*location	1	1	-0.0373	0.0513	14000	-0.73	0.4680	1.00
	BT	Year*quarter*location	2	2	-0.2104	0.0509	14000	-4.13	<.0001	**<.0001**
	BT	Year*quarter*location	3	3	-0.0957	0.0506	14000	-1.89	0.0586	1.00
	BT	Year*quarter*location	4	4	-0.1355	0.0509	14000	-2.66	0.0078	0.62
	WT	Year*quarter*location	1	1	-0.04935	0.09866	14000	-0.5	0.6169	1.00
	WT	Year*quarter*location	2	2	-0.3774	0.09785	14000	-3.86	0.0001	**0.01**
	WT	Year*quarter*location	3	3	-0.1432	0.09731	14000	-1.47	0.1411	1.00
	WT	Year*quarter*location	4	4	-0.212	0.09785	14000	-2.17	0.0303	1.00
India	TIB	Year*location	-	-	-0.2034	0.02898	14000	-7.02	<.0001	**<.0001**
	MOS	Year*location	-	-	-0.3900	0.0362	14000	-10.76	<.0001	**<.0001**
	BT	Year*location	-	-	-0.2883	0.02547	14000	-11.32	<.0001	**<.0001**
	WT	Year*location	-	-	-0.4917	0.04896	14000	-10.04	<.0001	**<.0001**
	TIB	Year*quarter*location	1	1	0.01838	0.05841	14000	0.31	0.753	1.00
	TIB	Year*quarter*location	2	2	-0.2243	0.05792	14000	-3.87	0.0001	**0.008**
	TIB	Year*quarter*location	3	3	-0.3663	0.05761	14000	-6.36	<.0001	**<.0001**
	TIB	Year*quarter*location	4	4	-0.2414	0.05792	14000	-4.17	<.0001	**<.0001**
	MOS	Year*quarter*location	1	1	-0.1277	0.07302	14000	-1.75	0.0803	1.00
	MOS	Year*quarter*location	2	2	-0.6435	0.07242	14000	-8.89	<.0001	**<.0001**
	MOS	Year*quarter*location	3	3	-0.5577	0.07202	14000	-7.74	<.0001	**<.0001**
	MOS	Year*quarter*location	4	4	-0.2312	0.07242	14000	-3.19	0.0014	0.11
	BT	Year*quarter*location	1	1	-0.1369	0.0513	14000	-2.67	0.0077	0.62
	BT	Year*quarter*location	2	2	-0.5313	0.0509	14000	-10.44	<.0001	**<.0001**
	BT	Year*quarter*location	3	3	-0.3745	0.0506	14000	-7.40	<.0001	**<.0001**
	BT	Year*quarter*location	4	4	-0.1105	0.0509	14000	-2.17	0.0299	1.00
	WT	Year*quarter*location	1	1	-0.1185	0.09866	14000	-1.2	0.2296	1.00
	WT	Year*quarter*location	2	2	-0.7556	0.09785	14000	-7.72	<.0001	**<.0001**
	WT	Year*quarter*location	3	3	-0.7409	0.09731	14000	-7.61	<.0001	**<.0001**
	WT	Year*quarter*location	4	4	-0.3519	0.09785	14000	-3.6	0.0003	**0.02**
Italy	TIB	Year*location	-	-	-0.1575	0.02898	14000	-5.44	<.0001	**<.0001**
	MOS	Year*location	-	-	-0.1793	0.0362	14000	-4.95	<.0001	**<.0001**
	BT	Year*location	-	-	-0.1006	0.02547	14000	-3.95	<.0001	**<.0001**
	WT	Year*location	-	-	-0.2581	0.04896	14000	-5.27	<.0001	**<.0001**
	TIB	Year*quarter*location	1	1	-0.1372	0.05841	14000	-2.35	0.0188	1.00
	TIB	Year*quarter*location	2	2	-0.2601	0.05792	14000	-4.49	<.0001	**<.0001**
	TIB	Year*quarter*location	3	3	-0.06558	0.05761	14000	-1.14	0.255	1.00
	TIB	Year*quarter*location	4	4	-0.1672	0.05792	14000	-2.89	0.0039	0.31
	MOS	Year*quarter*location	1	1	-0.1104	0.07302	14000	-1.51	0.1305	1.00
	MOS	Year*quarter*location	2	2	-0.3442	0.07242	14000	-4.75	<.0001	**<.0001**
	MOS	Year*quarter*location	3	3	-0.07754	0.07202	14000	-1.08	0.2817	1.00
	MOS	Year*quarter*location	4	4	-0.1851	0.07242	14000	-2.56	0.0106	0.85
	BT	Year*quarter*location	1	1	-0.0418	0.0513	14000	-0.81	0.4153	1.00
	BT	Year*quarter*location	2	2	-0.2142	0.0509	14000	-4.21	<.0001	**<.0001**
	BT	Year*quarter*location	3	3	-0.0448	0.0506	14000	-0.88	0.3768	1.00
	BT	Year*quarter*location	4	4	-0.1015	0.0509	14000	-1.99	0.0463	1.00
	WT	Year*quarter*location	1	1	-0.179	0.09866	14000	-1.81	0.0696	1.00
	WT	Year*quarter*location	2	2	-0.4743	0.09785	14000	-4.85	<.0001	**<.0001**
	WT	Year*quarter*location	3	3	-0.1103	0.09731	14000	-1.13	0.2569	1.00
	WT	Year*quarter*location	4	4	-0.2687	0.09785	14000	-2.75	0.0060	0.48

Japan	TIB	Year*location	-	-	-0.06731	0.02898	14000	-2.32	0.0202	0.40
	MOS	Year*location	-	-	0.0561	0.0362	14000	1.55	0.1215	1.00
	BT	Year*location	-	-	0.08977	0.02547	14000	3.52	0.0004	**0.01**
	WT	Year*location	-	-	0.02246	0.04896	14000	0.46	0.6465	1.00
	TIB	Year*quarter*location	1	1	-0.03859	0.05841	14000	-0.66	0.5088	1.00
	TIB	Year*quarter*location	2	2	-0.08214	0.05792	14000	-1.42	0.1562	1.000
	TIB	Year*quarter*location	3	3	-0.06024	0.05761	14000	-1.05	0.2957	1.00
	TIB	Year*quarter*location	4	4	-0.08828	0.05792	14000	-1.52	0.1275	1.00
	MOS	Year*quarter*location	1	1	0.07115	0.07302	14000	0.97	0.3299	1.00
	MOS	Year*quarter*location	2	2	-0.00124	0.07242	14000	-0.02	0.9864	1.00
	MOS	Year*quarter*location	3	3	0.0659	0.07202	14000	0.91	0.3602	1.00
	MOS	Year*quarter*location	4	4	0.08864	0.07242	14000	1.22	0.2209	1.00
	BT	Year*quarter*location	1	1	0.0904	0.0513	14000	1.76	0.0781	1.00
	BT	Year*quarter*location	2	2	0.0398	0.0509	14000	0.78	0.4339	1.00
	BT	Year*quarter*location	3	3	0.0960	0.0506	14000	1.90	0.0579	1.00
	BT	Year*quarter*location	4	4	0.1328	0.0509	14000	2.61	0.0091	0.73
	WT	Year*quarter*location	1	1	0.05185	0.09866	14000	0.53	0.5992	1.00
	WT	Year*quarter*location	2	2	-0.04231	0.09785	14000	-0.43	0.6655	1.00
	WT	Year*quarter*location	3	3	0.03578	0.09731	14000	0.37	0.7131	1.00
	WT	Year*quarter*location	4	4	0.04451	0.09785	14000	0.45	0.6492	1.00
Mexico	TIB	Year*location	-	-	-0.247	0.02898	14000	-8.52	<.0001	**<.0001**
	MOS	Year*location	-	-	-0.2298	0.0362	14000	-6.34	<.0001	**<.0001**
	BT	Year*location	-	-	-0.1063	0.02547	14000	-4.17	<.0001	**<.0001**
	WT	Year*location	-	-	-0.3533	0.04896	14000	-7.22	<.0001	**<.0001**
	TIB	Year*quarter*location	1	1	0.005767	0.05841	14000	0.1	0.9213	1.00
	TIB	Year*quarter*location	2	2	-0.197	0.05792	14000	-3.4	0.0007	0.056
	TIB	Year*quarter*location	3	3	-0.3516	0.05761	14000	-6.1	<.0001	**<.0001**
	TIB	Year*quarter*location	4	4	-0.4451	0.05792	14000	-7.69	<.0001	**<.0001**
	MOS	Year*quarter*location	1	1	0.1269	0.07302	14000	1.74	0.0822	1.00
	MOS	Year*quarter*location	2	2	-0.4841	0.07242	14000	-6.69	<.0001	**<.0001**
	MOS	Year*quarter*location	3	3	-0.3388	0.07202	14000	-4.7	<.0001	**<.0001**
	MOS	Year*quarter*location	4	4	-0.2234	0.07242	14000	-3.08	0.002	0.16
	BT	Year*quarter*location	1	1	0.1240	0.0513	14000	2.42	0.0157	1.00
	BT	Year*quarter*location	2	2	-0.3856	0.0509	14000	-7.58	<.0001	**<.0001**
	BT	Year*quarter*location	3	3	-0.1630	0.0506	14000	-3.22	0.0013	0.10
	BT	Year*quarter*location	4	4	-0.0008	0.0509	14000	-0.02	0.9871	1.00
	WT	Year*quarter*location	1	1	0.1298	0.09866	14000	1.32	0.1884	1.00
	WT	Year*quarter*location	2	2	-0.5826	0.09785	14000	-5.95	<.0001	**<.0001**
	WT	Year*quarter*location	3	3	-0.5146	0.09731	14000	-5.29	<.0001	**<.0001**
	WT	Year*quarter*location	4	4	-0.446	0.09785	14000	-4.56	<.0001	**<.0001**
Netherlands	TIB	Year*location	-	-	-0.07393	0.02898	14000	-2.55	0.0108	0.22
	MOS	Year*location	-	-	-0.1297	0.0362	14000	-3.58	0.0003	**0.01**
	BT	Year*location	-	-	-0.09271	0.02547	14000	-3.64	0.0003	**0.01**
	WT	Year*location	-	-	-0.1666	0.04896	14000	-3.4	0.0007	**0.01**
	TIB	Year*quarter*location	1	1	-0.02966	0.05841	14000	-0.51	0.6116	1.00
	TIB	Year*quarter*location	2	2	-0.1592	0.05792	14000	-2.75	0.006	0.480
	TIB	Year*quarter*location	3	3	-0.03668	0.05761	14000	-0.64	0.5242	1.00
	TIB	Year*quarter*location	4	4	-0.07024	0.05792	14000	-1.21	0.2253	1.00
	MOS	Year*quarter*location	1	1	-0.04946	0.07302	14000	-0.68	0.4982	1.00
	MOS	Year*quarter*location	2	2	-0.2036	0.07242	14000	-2.81	0.0049	0.39
	MOS	Year*quarter*location	3	3	-0.1075	0.07202	14000	-1.49	0.1357	1.00
	MOS	Year*quarter*location	4	4	-0.1582	0.07242	14000	-2.18	0.0289	1.00
	BT	Year*quarter*location	1	1	-0.0346	0.0513	14000	-0.67	0.4999	1.00
	BT	Year*quarter*location	2	2	-0.1240	0.0509	14000	-2.44	0.0149	1.00
	BT	Year*quarter*location	3	3	-0.0891	0.0506	14000	-1.76	0.0784	1.00
	BT	Year*quarter*location	4	4	-0.1231	0.0509	14000	-2.42	0.0156	1.00
	WT	Year*quarter*location	1	1	-0.06429	0.09866	14000	-0.65	0.5147	1.00
	WT	Year*quarter*location	2	2	-0.2832	0.09785	14000	-2.89	0.0038	0.30
	WT	Year*quarter*location	3	3	-0.1258	0.09731	14000	-1.29	0.1961	1.00
	WT	Year*quarter*location	4	4	-0.1933	0.09785	14000	-1.98	0.0482	1.00

New Zealand	TIB	Year*location	-	-	-0.01884	0.02898	14000	-0.65	0.5158	1.00
	MOS	Year*location	-	-	-0.1192	0.0362	14000	-3.29	0.0010	**0.02**
	BT	Year*location	-	-	-0.1098	0.02547	14000	-4.31	<.0001	**<.0001**
	WT	Year*location	-	-	-0.1286	0.04896	14000	-2.63	0.0086	0.17
	TIB	Year*quarter*location	1	1	-0.04101	0.05841	14000	-0.7	0.4826	1.00
	TIB	Year*quarter*location	2	2	-0.1044	0.05792	14000	-1.8	0.0715	1.000
	TIB	Year*quarter*location	3	3	0.1489	0.05761	14000	2.59	0.0097	0.78
	TIB	Year*quarter*location	4	4	-0.07885	0.05792	14000	-1.36	0.1735	1.00
	MOS	Year*quarter*location	1	1	0.06347	0.07302	14000	0.87	0.3848	1.00
	MOS	Year*quarter*location	2	2	-0.3021	0.07242	14000	-4.17	<.0001	**<.0001**
	MOS	Year*quarter*location	3	3	-0.1451	0.07202	14000	-2.01	0.0439	1.00
	MOS	Year*quarter*location	4	4	-0.09318	0.07242	14000	-1.29	0.1982	1.00
	BT	Year*quarter*location	1	1	0.0840	0.0513	14000	1.64	0.1019	1.00
	BT	Year*quarter*location	2	2	-0.2499	0.0509	14000	-4.91	<.0001	**<.0001**
	BT	Year*quarter*location	3	3	-0.2196	0.0506	14000	-4.34	<.0001	**<.0001**
	BT	Year*quarter*location	4	4	-0.0538	0.0509	14000	-1.06	0.2910	1.00
	WT	Year*quarter*location	1	1	0.04296	0.09866	14000	0.44	0.6633	1.00
	WT	Year*quarter*location	2	2	-0.3543	0.09785	14000	-3.62	0.0003	**0.02**
	WT	Year*quarter*location	3	3	-0.07065	0.09731	14000	-0.73	0.4678	1.00
	WT	Year*quarter*location	4	4	-0.1326	0.09785	14000	-1.36	0.1754	1.00
Poland	TIB	Year*location	-	-	-0.1122	0.02898	14000	-3.87	0.0001	**0.002**
	MOS	Year*location	-	-	-0.2004	0.03623	14000	-5.53	<.0001	**<.0001**
	BT	Year*location	-	-	-0.1443	0.02547	14000	-5.67	<.0001	**<.0001**
	WT	Year*location	-	-	-0.2565	0.04896	14000	-5.24	<.0001	**<.0001**
	TIB	Year*quarter*location	1	1	-0.04977	0.05841	14000	-0.85	0.3941	1.00
	TIB	Year*quarter*location	2	2	-0.1712	0.05792	14000	-2.96	0.0031	0.248
	TIB	Year*quarter*location	3	3	-0.07908	0.05761	14000	-1.37	0.1699	1.00
	TIB	Year*quarter*location	4	4	-0.1485	0.05792	14000	-2.56	0.0103	0.82
	MOS	Year*quarter*location	1	1	-0.1322	0.07302	14000	-1.81	0.0702	1.00
	MOS	Year*quarter*location	2	2	-0.318	0.07242	14000	-4.39	<.0001	**<.0001**
	MOS	Year*quarter*location	3	3	-0.1399	0.07202	14000	-1.94	0.0521	1.00
	MOS	Year*quarter*location	4	4	-0.2115	0.07242	14000	-2.92	0.0035	0.28
	BT	Year*quarter*location	1	1	-0.1073	0.0513	14000	-2.09	0.0365	1.00
	BT	Year*quarter*location	2	2	-0.2324	0.0509	14000	-4.57	<.0001	**<.0001**
	BT	Year*quarter*location	3	3	-0.1004	0.0506	14000	-1.98	0.0475	1.00
	BT	Year*quarter*location	4	4	-0.1373	0.0509	14000	-2.70	0.0070	0.56
	WT	Year*quarter*location	1	1	-0.1571	0.09866	14000	-1.59	0.1113	1.00
	WT	Year*quarter*location	2	2	-0.4037	0.09785	14000	-4.13	<.0001	**<.0001**
	WT	Year*quarter*location	3	3	-0.1794	0.09731	14000	-1.84	0.0652	1.00
	WT	Year*quarter*location	4	4	-0.2858	0.09785	14000	-2.92	0.0035	0.28
Russia	TIB	Year*location	-	-	-0.1492	0.02898	14000	-5.15	<.0001	**<.0001**
	MOS	Year*location	-	-	-0.3028	0.0362	14000	-8.36	<.0001	**<.0001**
	BT	Year*location	-	-	-0.2282	0.02547	14000	-8.96	<.0001	**<.0001**
	WT	Year*location	-	-	-0.3775	0.04896	14000	-7.71	<.0001	**<.0001**
	TIB	Year*quarter*location	1	1	-0.1054	0.05841	14000	-1.8	0.0711	1.00
	TIB	Year*quarter*location	2	2	-0.2316	0.05792	14000	-4	<.0001	**<.0001**
	TIB	Year*quarter*location	3	3	-0.08315	0.05761	14000	-1.44	0.1489	1.00
	TIB	Year*quarter*location	4	4	-0.1768	0.05792	14000	-3.05	0.0023	0.18
	MOS	Year*quarter*location	1	1	-0.07691	0.07302	14000	-1.05	0.2923	1.00
	MOS	Year*quarter*location	2	2	-0.5526	0.07242	14000	-7.63	<.0001	**<.0001**
	MOS	Year*quarter*location	3	3	-0.2421	0.07202	14000	-3.36	0.0008	0.06
	MOS	Year*quarter*location	4	4	-0.3397	0.07242	14000	-4.69	<.0001	**<.0001**
	BT	Year*quarter*location	1	1	-0.0242	0.0513	14000	-0.47	0.6374	1.00
	BT	Year*quarter*location	2	2	-0.4368	0.0509	14000	-8.58	<.0001	**<.0001**
	BT	Year*quarter*location	3	3	-0.2005	0.0506	14000	-3.96	<.0001	**<.0001**
	BT	Year*quarter*location	4	4	-0.2513	0.0509	14000	-4.94	<.0001	**<.0001**
	WT	Year*quarter*location	1	1	-0.1296	0.09866	14000	-1.31	0.1890	1.00
	WT	Year*quarter*location	2	2	-0.6684	0.09785	14000	-6.83	<.0001	**<.0001**
	WT	Year*quarter*location	3	3	-0.2837	0.09731	14000	-2.92	0.0036	0.29
	WT	Year*quarter*location	4	4	-0.4281	0.09785	14000	-4.38	<.0001	**<.0001**

South Africa	TIB	Year*location	-	-	-0.05481	0.02898	14000	-1.89	0.0586	1.00
	MOS	Year*location	-	-	-0.3867	0.0362	14000	-10.67	<.0001	**<.0001**
	BT	Year*location	-	-	-0.3593	0.02547	14000	-14.1	<.0001	**<.0001**
	WT	Year*location	-	-	-0.4141	0.04896	14000	-8.46	<.0001	**<.0001**
	TIB	Year*quarter*location	1	1	-0.1112	0.05841	14000	-1.9	0.057	1.00
	TIB	Year*quarter*location	2	2	-0.106	0.05792	14000	-1.83	0.0674	1.000
	TIB	Year*quarter*location	3	3	-0.00172	0.05761	14000	-0.03	0.9762	1.00
	TIB	Year*quarter*location	4	4	-0.00037	0.05792	14000	-0.01	0.995	1.00
	MOS	Year*quarter*location	1	1	-0.0604	0.07302	14000	-0.83	0.4081	1.00
	MOS	Year*quarter*location	2	2	-0.6368	0.07242	14000	-8.79	<.0001	**<.0001**
	MOS	Year*quarter*location	3	3	-0.4741	0.07202	14000	-6.58	<.0001	**<.0001**
	MOS	Year*quarter*location	4	4	-0.3754	0.07242	14000	-5.18	<.0001	**<.0001**
	BT	Year*quarter*location	1	1	-0.0048	0.0513	14000	-0.09	0.9254	1.00
	BT	Year*quarter*location	2	2	-0.5838	0.0509	14000	-11.47	<.0001	**<.0001**
	BT	Year*quarter*location	3	3	-0.4733	0.0506	14000	-9.35	<.0001	**<.0001**
	BT	Year*quarter*location	4	4	-0.3752	0.0509	14000	-7.37	<.0001	**<.0001**
	WT	Year*quarter*location	1	1	-0.116	0.09866	14000	-1.18	0.2397	1.00
	WT	Year*quarter*location	2	2	-0.6897	0.09785	14000	-7.05	<.0001	**<.0001**
	WT	Year*quarter*location	3	3	-0.475	0.09731	14000	-4.88	<.0001	**<.0001**
	WT	Year*quarter*location	4	4	-0.3755	0.09785	14000	-3.84	0.0001	**0.01**
South Korea	TIB	Year*location	-	-	-0.05922	0.02898	14000	-2.04	0.041	0.82
	MOS	Year*location	-	-	-0.01921	0.03623	14000	-0.53	0.5959	1.00
	BT	Year*location	-	-	0.0104	0.02547	14000	0.41	0.6832	1.00
	WT	Year*location	-	-	-0.04882	0.04896	14000	-1	0.3187	1.00
	TIB	Year*quarter*location	1	1	0.03602	0.05841	14000	0.62	0.5374	1.00
	TIB	Year*quarter*location	2	2	-0.1312	0.05792	14000	-2.27	0.0235	1.000
	TIB	Year*quarter*location	3	3	-0.0577	0.05761	14000	-1	0.3165	1.00
	TIB	Year*quarter*location	4	4	-0.08397	0.05792	14000	-1.45	0.1471	1.00
	MOS	Year*quarter*location	1	1	-0.01929	0.07302	14000	-0.26	0.7917	1.00
	MOS	Year*quarter*location	2	2	-0.06836	0.07242	14000	-0.94	0.3452	1.00
	MOS	Year*quarter*location	3	3	0.001132	0.07202	14000	0.02	0.9875	1.00
	MOS	Year*quarter*location	4	4	0.009661	0.07242	14000	0.13	0.8939	1.00
	BT	Year*quarter*location	1	1	-0.0373	0.0513	14000	-0.73	0.4675	1.00
	BT	Year*quarter*location	2	2	-0.0028	0.0509	14000	-0.05	0.9570	1.00
	BT	Year*quarter*location	3	3	0.0300	0.0506	14000	0.59	0.5537	1.00
	BT	Year*quarter*location	4	4	0.0517	0.0509	14000	1.01	0.3103	1.00
	WT	Year*quarter*location	1	1	-0.00128	0.09866	14000	-0.01	0.9897	1.00
	WT	Year*quarter*location	2	2	-0.134	0.09785	14000	-1.37	0.1709	1.00
	WT	Year*quarter*location	3	3	-0.02772	0.09731	14000	-0.28	0.7758	1.00
	WT	Year*quarter*location	4	4	-0.03233	0.09785	14000	-0.33	0.7411	1.00
Sweden	TIB	Year*location	-	-	0.01424	0.02898	14000	0.49	0.6232	1.00
	MOS	Year*location	-	-	-0.1432	0.03623	14000	-3.95	<.0001	**<.0001**
	BT	Year*location	-	-	-0.1503	0.02547	14000	-5.9	<.0001	**<.0001**
	WT	Year*location	-	-	-0.1361	0.04896	14000	-2.78	0.0055	0.11
	TIB	Year*quarter*location	1	1	-0.01023	0.05841	14000	-0.18	0.861	1.00
	TIB	Year*quarter*location	2	2	0.01603	0.05792	14000	0.28	0.782	1.000
	TIB	Year*quarter*location	3	3	0.06857	0.05761	14000	1.19	0.2339	1.00
	TIB	Year*quarter*location	4	4	-0.0174	0.05792	14000	-0.3	0.7639	1.00
	MOS	Year*quarter*location	1	1	-0.06412	0.07302	14000	-0.88	0.3799	1.00
	MOS	Year*quarter*location	2	2	-0.1576	0.07242	14000	-2.18	0.0295	1.00
	MOS	Year*quarter*location	3	3	-0.1346	0.07202	14000	-1.87	0.0617	1.00
	MOS	Year*quarter*location	4	4	-0.2164	0.07242	14000	-2.99	0.0028	0.22
	BT	Year*quarter*location	1	1	-0.0590	0.0513	14000	-1.15	0.2504	1.00
	BT	Year*quarter*location	2	2	-0.1657	0.0509	14000	-3.25	0.0011	0.09
	BT	Year*quarter*location	3	3	-0.1688	0.0506	14000	-3.33	0.0009	0.07
	BT	Year*quarter*location	4	4	-0.2077	0.0509	14000	-4.08	<.0001	**<.0001**
	WT	Year*quarter*location	1	1	-0.06923	0.09866	14000	-0.7	0.4829	1.00
	WT	Year*quarter*location	2	2	-0.1496	0.09785	14000	-1.53	0.1262	1.00
	WT	Year*quarter*location	3	3	-0.1003	0.09731	14000	-1.03	0.3028	1.00
	WT	Year*quarter*location	4	4	-0.2251	0.09785	14000	-2.3	0.0214	1.00

United Kingdom	TIB	Year*location	-	-	-0.06719	0.02898	14000	-2.32	0.0204	0.41
	MOS	Year*location	-	-	-0.2539	0.03623	14000	-7.01	<.0001	**<.0001**
	BT	Year*location	-	-	-0.2203	0.02547	14000	-8.65	<.0001	**<.0001**
	WT	Year*location	-	-	-0.2875	0.04896	14000	-5.87	<.0001	**<.0001**
	TIB	Year*quarter*location	1	1	0.05749	0.05841	14000	0.98	0.325	1.00
	TIB	Year*quarter*location	2	2	-0.1982	0.05792	14000	-3.42	0.0006	**0.048**
	TIB	Year*quarter*location	3	3	-0.04457	0.05761	14000	-0.77	0.4392	1.00
	TIB	Year*quarter*location	4	4	-0.08352	0.05792	14000	-1.44	0.1494	1.00
	MOS	Year*quarter*location	1	1	-0.04921	0.07302	14000	-0.67	0.5004	1.00
	MOS	Year*quarter*location	2	2	-0.4206	0.07242	14000	-5.81	<.0001	**<.0001**
	MOS	Year*quarter*location	3	3	-0.2867	0.07202	14000	-3.98	<.0001	**<.0001**
	MOS	Year*quarter*location	4	4	-0.259	0.07242	14000	-3.58	0.0003	**0.02**
	BT	Year*quarter*location	1	1	-0.0780	0.0513	14000	-1.52	0.1289	1.00
	BT	Year*quarter*location	2	2	-0.3215	0.0509	14000	-6.32	<.0001	**<.0001**
	BT	Year*quarter*location	3	3	-0.2644	0.0506	14000	-5.22	<.0001	**<.0001**
	BT	Year*quarter*location	4	4	-0.2172	0.0509	14000	-4.27	<.0001	**<.0001**
	WT	Year*quarter*location	1	1	-0.02047	0.09866	14000	-0.21	0.8357	1.00
	WT	Year*quarter*location	2	2	-0.5197	0.09785	14000	-5.31	<.0001	**<.0001**
	WT	Year*quarter*location	3	3	-0.309	0.09731	14000	-3.17	0.0015	0.12
	WT	Year*quarter*location	4	4	-0.3007	0.09785	14000	-3.07	0.0021	0.17
United States	TIB	Year*location	-	-	-0.09615	0.02898	14000	-3.32	0.0009	**0.02**
	MOS	Year*location	-	-	-0.2445	0.03623	14000	-6.75	<.0001	**<.0001**
	BT	Year*location	-	-	-0.1965	0.02547	14000	-7.71	<.0001	**<.0001**
	WT	Year*location	-	-	-0.2926	0.04896	14000	-5.98	<.0001	**<.0001**
	TIB	Year*quarter*location	1	1	-0.02311	0.05841	14000	-0.4	0.6924	1.00
	TIB	Year*quarter*location	2	2	-0.1809	0.05792	14000	-3.12	0.0018	0.144
	TIB	Year*quarter*location	3	3	-0.04574	0.05761	14000	-0.79	0.4272	1.00
	TIB	Year*quarter*location	4	4	-0.1349	0.05792	14000	-2.33	0.0199	1.00
	MOS	Year*quarter*location	1	1	-0.02648	0.07302	14000	-0.36	0.7169	1.00
	MOS	Year*quarter*location	2	2	-0.4057	0.07242	14000	-5.6	<.0001	**<.0001**
	MOS	Year*quarter*location	3	3	-0.2691	0.07202	14000	-3.74	0.0002	**0.02**
	MOS	Year*quarter*location	4	4	-0.2769	0.07242	14000	-3.82	0.0001	**0.01**
	BT	Year*quarter*location	1	1	-0.01493	0.05133	14000	-0.29	0.7712	1.00
	BT	Year*quarter*location	2	2	-0.3153	0.05091	14000	-6.19	<.0001	**<.0001**
	BT	Year*quarter*location	3	3	-0.2462	0.05063	14000	-4.86	<.0001	**<.0001**
	BT	Year*quarter*location	4	4	-0.2094	0.05091	14000	-4.11	<.0001	**<.0001**
	WT	Year*quarter*location	1	1	-0.03803	0.09866	14000	-0.39	0.6999	1.00
	WT	Year*quarter*location	2	2	-0.4962	0.09785	14000	-5.07	<.0001	**<.0001**
	WT	Year*quarter*location	3	3	-0.2919	0.09731	14000	-3	0.0027	0.22
	WT	Year*quarter*location	4	4	-0.3443	0.09785	14000	-3.52	0.0004	**0.03**

Bonferroni adjustments were made for 20 comparisons in the year*location analysis (20 countries in 2019 vs. the same country in 2020), and 80 comparisons in the year*quarter*location analysis (each quarter of 2019 vs. the same quarter in 2020, for each of 20 countries)

Least Square Means										
Outcome	Effect	Country	Month 2019	Month 2020	Estimate	Standard Error	Degrees of Freedom	T-value	P-value	Adjusted p-value
TIB	Year*Month*Location	United States	1	1	-0.0364	0.1008	14000	-0.36	0.7182	8.6184
TIB	Year*Month*Location	United States	2	2	0.0125	0.1035	14000	0.12	0.9042	10.8504
TIB	Year*Month*Location	United States	3	3	-0.0438	0.0992	14000	-0.44	0.6587	7.9044
TIB	Year*Month*Location	United States	4	4	-0.2050	0.1008	14000	-2.03	0.0421	0.5052
TIB	Year*Month*Location	United States	5	5	-0.2234	0.0992	14000	-2.25	0.0243	0.2916
TIB	Year*Month*Location	United States	6	6	-0.1128	0.1008	14000	-1.12	0.2634	3.1608
TIB	Year*Month*Location	United States	7	7	-0.1392	0.0992	14000	-1.4	0.1604	1.9248
TIB	Year*Month*Location	United States	8	8	-0.0847	0.0992	14000	-0.85	0.3933	4.7196
TIB	Year*Month*Location	United States	9	9	0.0911	0.1008	14000	0.9	0.3662	4.3944
TIB	Year*Month*Location	United States	10	10	-0.1269	0.0992	14000	-1.28	0.2008	2.4096
TIB	Year*Month*Location	United States	11	11	-0.1158	0.1008	14000	-1.15	0.2506	3.0072
TIB	Year*Month*Location	United States	12	12	-0.1622	0.1008	14000	-1.61	0.1077	1.2924
MOS	Year*Month*Location	United States	1	1	0.0613	0.1249	14000	0.49	0.6238	7.4856
MOS	Year*Month*Location	United States	2	2	0.0415	0.1282	14000	0.32	0.7464	8.9568
MOS	Year*Month*Location	United States	3	3	-0.1757	0.1229	14000	-1.43	0.1528	1.8336
MOS	Year*Month*Location	United States	4	4	-0.4894	0.1249	14000	-3.92	<.0001	**<.0001**
MOS	Year*Month*Location	United States	5	5	-0.4663	0.1229	14000	-3.79	0.0001	**0.0012**
MOS	Year*Month*Location	United States	6	6	-0.2594	0.1249	14000	-2.08	0.0378	0.4536
MOS	Year*Month*Location	United States	7	7	-0.2478	0.1229	14000	-2.02	0.0437	0.5244
MOS	Year*Month*Location	United States	8	8	-0.3055	0.1229	14000	-2.49	0.0129	0.1548
MOS	Year*Month*Location	United States	9	9	-0.2533	0.1249	14000	-2.03	0.0425	0.5100
MOS	Year*Month*Location	United States	10	10	-0.3253	0.1229	14000	-2.65	0.0081	0.0972
MOS	Year*Month*Location	United States	11	11	-0.2649	0.1249	14000	-2.12	0.0340	0.4080
MOS	Year*Month*Location	United States	12	12	-0.2389	0.1249	14000	-1.91	0.0558	0.6696
BT	Year*Month*Location	United States	1	1	0.0794	0.0865	14000	0.92	0.3583	4.2996
BT	Year*Month*Location	United States	2	2	0.0352	0.0887	14000	0.40	0.6914	8.2968
BT	Year*Month*Location	United States	3	3	-0.1538	0.0851	14000	-1.81	0.0707	0.8484
BT	Year*Month*Location	United States	4	4	-0.3869	0.0865	14000	-4.47	<.0001	**<.0001**
BT	Year*Month*Location	United States	5	5	-0.3546	0.0851	14000	-4.17	<.0001	**<.0001**
BT	Year*Month*Location	United States	6	6	-0.2031	0.0865	14000	-2.35	0.0189	0.2268
BT	Year*Month*Location	United States	7	7	-0.1782	0.0851	14000	-2.09	0.0362	0.4344
BT	Year*Month*Location	United States	8	8	-0.2632	0.0851	14000	-3.09	0.0020	**0.0240**
BT	Year*Month*Location	United States	9	9	-0.2989	0.0865	14000	-3.46	0.0005	**0.0060**
BT	Year*Month*Location	United States	10	10	-0.2618	0.0851	14000	-3.08	0.0021	**0.0252**
BT	Year*Month*Location	United States	11	11	-0.2069	0.0865	14000	-2.39	0.0167	0.2004
BT	Year*Month*Location	United States	12	12	-0.1578	0.0865	14000	-1.82	0.0681	0.8172
WT	Year*Month*Location	United States	1	1	0.0431	0.1697	14000	0.25	0.7997	9.5964
WT	Year*Month*Location	United States	2	2	0.0477	0.1741	14000	0.27	0.7842	9.4104
WT	Year*Month*Location	United States	3	3	-0.1976	0.1670	14000	-1.18	0.2366	2.8392
WT	Year*Month*Location	United States	4	4	-0.5919	0.1697	14000	-3.49	0.0005	**0.0060**
WT	Year*Month*Location	United States	5	5	-0.5780	0.1670	14000	-3.46	0.0005	**0.0060**
WT	Year*Month*Location	United States	6	6	-0.3158	0.1697	14000	-1.86	0.0628	0.7536
WT	Year*Month*Location	United States	7	7	-0.3175	0.1670	14000	-1.90	0.0572	0.6864
WT	Year*Month*Location	United States	8	8	-0.3478	0.1670	14000	-2.08	0.0372	0.4464
WT	Year*Month*Location	United States	9	9	-0.2078	0.1697	14000	-1.22	0.2209	2.6508
WT	Year*Month*Location	United States	10	10	-0.3887	0.1670	14000	-2.33	0.0199	0.2388
WT	Year*Month*Location	United States	11	11	-0.3228	0.1697	14000	-1.90	0.0572	0.6864
WT	Year*Month*Location	United States	12	12	-0.3200	0.1697	14000	-1.89	0.0594	0.7128

Bonferroni adjustments were made for 12 comparisons (each month of 2019 to the corresponding month in 2020, for the U.S. only)

The below data is not included in our analysis, but we have provided it as supplemental for others who may be interested

Least Square Means									
Outcome	Effect	Country	Month 2019	Month 2020	Estimate	Standard Error	Degrees of Freedom	T-value	P-value
WT	Year*Month*Location	Australia	1	1	-0.0225	0.1697	14000	-0.13	0.8945
WT	Year*Month*Location	Australia	2	2	-0.0989	0.1741	14000	-0.57	0.5701
WT	Year*Month*Location	Australia	3	3	-0.1930	0.1670	14000	-1.16	0.2477
WT	Year*Month*Location	Australia	4	4	-0.3958	0.1697	14000	-2.33	0.0197
WT	Year*Month*Location	Australia	5	5	-0.5177	0.1670	14000	-3.10	0.0019
WT	Year*Month*Location	Australia	6	6	-0.1772	0.1697	14000	-1.04	0.2964
WT	Year*Month*Location	Australia	7	7	-0.2833	0.1670	14000	-1.70	0.0897
WT	Year*Month*Location	Australia	8	8	-0.2968	0.1670	14000	-1.78	0.0755
WT	Year*Month*Location	Australia	9	9	-0.1936	0.1697	14000	-1.14	0.2540
WT	Year*Month*Location	Australia	10	10	-0.2704	0.1670	14000	-1.62	0.1053
WT	Year*Month*Location	Australia	11	11	-0.1017	0.1697	14000	-0.60	0.5491
WT	Year*Month*Location	Australia	12	12	-0.0286	0.1697	14000	-0.17	0.8661
WT	Year*Month*Location	Brazil	1	1	0.1544	0.1697	14000	0.91	0.3628
WT	Year*Month*Location	Brazil	2	2	-0.0548	0.1741	14000	-0.31	0.7528
WT	Year*Month*Location	Brazil	3	3	-0.0333	0.1670	14000	-0.20	0.8417
WT	Year*Month*Location	Brazil	4	4	-0.7078	0.1697	14000	-4.17	<.0001
WT	Year*Month*Location	Brazil	5	5	-0.6282	0.1670	14000	-3.76	0.0002
WT	Year*Month*Location	Brazil	6	6	-0.2617	0.1697	14000	-1.54	0.1231
WT	Year*Month*Location	Brazil	7	7	-0.1906	0.1670	14000	-1.14	0.2536
WT	Year*Month*Location	Brazil	8	8	-0.4285	0.1670	14000	-2.57	0.0103
WT	Year*Month*Location	Brazil	9	9	-0.2600	0.1697	14000	-1.53	0.1255
WT	Year*Month*Location	Brazil	10	10	-0.3083	0.1670	14000	-1.85	0.0648
WT	Year*Month*Location	Brazil	11	11	-0.3003	0.1697	14000	-1.77	0.0769
WT	Year*Month*Location	Brazil	12	12	-0.0436	0.1697	14000	-0.26	0.7972
WT	Year*Month*Location	Canada	1	1	-0.0517	0.1697	14000	-0.30	0.7608
WT	Year*Month*Location	Canada	2	2	-0.0817	0.1741	14000	-0.47	0.6392
WT	Year*Month*Location	Canada	3	3	-0.2153	0.1670	14000	-1.29	0.1972
WT	Year*Month*Location	Canada	4	4	-0.5547	0.1697	14000	-3.27	0.0011
WT	Year*Month*Location	Canada	5	5	-0.6159	0.1670	14000	-3.69	0.0002
WT	Year*Month*Location	Canada	6	6	-0.3814	0.1697	14000	-2.25	0.0246
WT	Year*Month*Location	Canada	7	7	-0.3137	0.1670	14000	-1.88	0.0603
WT	Year*Month*Location	Canada	8	8	-0.2780	0.1670	14000	-1.66	0.0960
WT	Year*Month*Location	Canada	9	9	-0.1817	0.1697	14000	-1.07	0.2844
WT	Year*Month*Location	Canada	10	10	-0.2565	0.1670	14000	-1.54	0.1245
WT	Year*Month*Location	Canada	11	11	-0.1942	0.1697	14000	-1.14	0.2526
WT	Year*Month*Location	Canada	12	12	-0.1964	0.1697	14000	-1.16	0.2472
WT	Year*Month*Location	China	1	1	-0.1658	0.1697	14000	-0.98	0.3285
WT	Year*Month*Location	China	2	2	-0.3229	0.1741	14000	-1.85	0.0637
WT	Year*Month*Location	China	3	3	-0.3798	0.1670	14000	-2.28	0.0229
WT	Year*Month*Location	China	4	4	-0.4075	0.1697	14000	-2.40	0.0164
WT	Year*Month*Location	China	5	5	-0.2272	0.1670	14000	-1.36	0.1737
WT	Year*Month*Location	China	6	6	-0.0400	0.1697	14000	-0.24	0.8137
WT	Year*Month*Location	China	7	7	-0.1511	0.1670	14000	-0.90	0.3655
WT	Year*Month*Location	China	8	8	-0.0640	0.1670	14000	-0.38	0.7016
WT	Year*Month*Location	China	9	9	-0.0103	0.1697	14000	-0.06	0.9517
WT	Year*Month*Location	China	10	10	-0.0376	0.1670	14000	-0.23	0.8217
WT	Year*Month*Location	China	11	11	-0.0425	0.1697	14000	-0.25	0.8023
WT	Year*Month*Location	China	12	12	-0.0119	0.1697	14000	-0.07	0.9439
WT	Year*Month*Location	Finland	1	1	-0.2433	0.1697	14000	-1.43	0.1516
WT	Year*Month*Location	Finland	2	2	-0.2709	0.1741	14000	-1.56	0.1199
WT	Year*Month*Location	Finland	3	3	-0.3978	0.1670	14000	-2.38	0.0172
WT	Year*Month*Location	Finland	4	4	-0.5969	0.1697	14000	-3.52	0.0004
WT	Year*Month*Location	Finland	5	5	-0.5855	0.1670	14000	-3.51	0.0005
WT	Year*Month*Location	Finland	6	6	-0.3614	0.1697	14000	-2.13	0.0332
WT	Year*Month*Location	Finland	7	7	-0.3070	0.1670	14000	-1.84	0.0660
WT	Year*Month*Location	Finland	8	8	-0.4247	0.1670	14000	-2.54	0.0110
WT	Year*Month*Location	Finland	9	9	-0.2503	0.1697	14000	-1.47	0.1403
WT	Year*Month*Location	Finland	10	10	-0.2844	0.1670	14000	-1.70	0.0885
WT	Year*Month*Location	Finland	11	11	-0.2644	0.1697	14000	-1.56	0.1192
WT	Year*Month*Location	Finland	12	12	0.0106	0.1697	14000	0.06	0.9504
WT	Year*Month*Location	France	1	1	0.0456	0.1697	14000	0.27	0.7884
WT	Year*Month*Location	France	2	2	0.0002	0.1741	14000	0.00	0.9991
WT	Year*Month*Location	France	3	3	-0.3137	0.1670	14000	-1.88	0.0603
WT	Year*Month*Location	France	4	4	-0.7167	0.1697	14000	-4.22	<.0001

WT	Year*Month*Location	France	5	5	-0.5153	0.1670	14000	-3.09	0.0020
WT	Year*Month*Location	France	6	6	-0.1933	0.1697	14000	-1.14	0.2546
WT	Year*Month*Location	France	7	7	-0.1919	0.1670	14000	-1.15	0.2503
WT	Year*Month*Location	France	8	8	-0.0194	0.1670	14000	-0.12	0.9077
WT	Year*Month*Location	France	9	9	-0.0408	0.1697	14000	-0.24	0.8099
WT	Year*Month*Location	France	10	10	-0.2263	0.1670	14000	-1.36	0.1752
WT	Year*Month*Location	France	11	11	-0.2900	0.1697	14000	-1.71	0.0875
WT	Year*Month*Location	France	12	12	-0.2019	0.1697	14000	-1.19	0.2341
WT	Year*Month*Location	Germany	1	1	0.0214	0.1697	14000	0.13	0.8997
WT	Year*Month*Location	Germany	2	2	-0.0363	0.1741	14000	-0.21	0.8349
WT	Year*Month*Location	Germany	3	3	-0.1317	0.1670	14000	-0.79	0.4301
WT	Year*Month*Location	Germany	4	4	-0.4842	0.1697	14000	-2.85	0.0043
WT	Year*Month*Location	Germany	5	5	-0.4879	0.1670	14000	-2.92	0.0035
WT	Year*Month*Location	Germany	6	6	-0.1564	0.1697	14000	-0.92	0.3568
WT	Year*Month*Location	Germany	7	7	-0.2132	0.1670	14000	-1.28	0.2017
WT	Year*Month*Location	Germany	8	8	-0.1454	0.1670	14000	-0.87	0.3837
WT	Year*Month*Location	Germany	9	9	-0.0686	0.1697	14000	-0.40	0.6860
WT	Year*Month*Location	Germany	10	10	-0.1871	0.1670	14000	-1.12	0.2624
WT	Year*Month*Location	Germany	11	11	-0.2314	0.1697	14000	-1.36	0.1728
WT	Year*Month*Location	Germany	12	12	-0.2183	0.1697	14000	-1.29	0.1983
WT	Year*Month*Location	India	1	1	0.0214	0.1697	14000	0.13	0.8997
WT	Year*Month*Location	India	2	2	-0.0617	0.1741	14000	-0.35	0.7231
WT	Year*Month*Location	India	3	3	-0.3094	0.1670	14000	-1.85	0.0639
WT	Year*Month*Location	India	4	4	-0.8217	0.1697	14000	-4.84	<.0001
WT	Year*Month*Location	India	5	5	-0.7981	0.1670	14000	-4.78	<.0001
WT	Year*Month*Location	India	6	6	-0.6456	0.1697	14000	-3.80	0.0001
WT	Year*Month*Location	India	7	7	-0.8487	0.1670	14000	-5.08	<.0001
WT	Year*Month*Location	India	8	8	-0.7608	0.1670	14000	-4.56	<.0001
WT	Year*Month*Location	India	9	9	-0.6089	0.1697	14000	-3.59	0.0003
WT	Year*Month*Location	India	10	10	-0.4656	0.1670	14000	-2.79	0.0053
WT	Year*Month*Location	India	11	11	-0.4733	0.1697	14000	-2.79	0.0053
WT	Year*Month*Location	India	12	12	-0.1131	0.1697	14000	-0.67	0.5053
WT	Year*Month*Location	Italy	1	1	-0.0136	0.1697	14000	-0.08	0.9361
WT	Year*Month*Location	Italy	2	2	-0.0321	0.1741	14000	-0.18	0.8537
WT	Year*Month*Location	Italy	3	3	-0.4772	0.1670	14000	-2.86	0.0043
WT	Year*Month*Location	Italy	4	4	-0.6122	0.1697	14000	-3.61	0.0003
WT	Year*Month*Location	Italy	5	5	-0.5210	0.1670	14000	-3.12	0.0018
WT	Year*Month*Location	Italy	6	6	-0.2881	0.1697	14000	-1.70	0.0897
WT	Year*Month*Location	Italy	7	7	-0.1987	0.1670	14000	-1.19	0.2341
WT	Year*Month*Location	Italy	8	8	-0.0285	0.1670	14000	-0.17	0.8645
WT	Year*Month*Location	Italy	9	9	-0.1036	0.1697	14000	-0.61	0.5415
WT	Year*Month*Location	Italy	10	10	-0.3538	0.1670	14000	-2.12	0.0341
WT	Year*Month*Location	Italy	11	11	-0.2869	0.1697	14000	-1.69	0.0909
WT	Year*Month*Location	Italy	12	12	-0.1625	0.1697	14000	-0.96	0.3383
WT	Year*Month*Location	Japan	1	1	0.0644	0.1697	14000	0.38	0.7041
WT	Year*Month*Location	Japan	2	2	0.0423	0.1741	14000	0.24	0.8080
WT	Year*Month*Location	Japan	3	3	0.0476	0.1670	14000	0.28	0.7756
WT	Year*Month*Location	Japan	4	4	-0.0408	0.1697	14000	-0.24	0.8099
WT	Year*Month*Location	Japan	5	5	-0.1183	0.1670	14000	-0.71	0.4787
WT	Year*Month*Location	Japan	6	6	0.0347	0.1697	14000	0.20	0.8379
WT	Year*Month*Location	Japan	7	7	-0.0035	0.1670	14000	-0.02	0.9833
WT	Year*Month*Location	Japan	8	8	0.0296	0.1670	14000	0.18	0.8594
WT	Year*Month*Location	Japan	9	9	0.0828	0.1697	14000	0.49	0.6257
WT	Year*Month*Location	Japan	10	10	0.0605	0.1670	14000	0.36	0.7171
WT	Year*Month*Location	Japan	11	11	0.0356	0.1697	14000	0.21	0.8341
WT	Year*Month*Location	Japan	12	12	0.0369	0.1697	14000	0.22	0.8277
WT	Year*Month*Location	Mexico	1	1	0.2258	0.1697	14000	1.33	0.1833
WT	Year*Month*Location	Mexico	2	2	0.1512	0.1741	14000	0.87	0.3853
WT	Year*Month*Location	Mexico	3	3	0.0151	0.1670	14000	0.09	0.9282
WT	Year*Month*Location	Mexico	4	4	-0.4944	0.1697	14000	-2.91	0.0036
WT	Year*Month*Location	Mexico	5	5	-0.7223	0.1670	14000	-4.33	<.0001
WT	Year*Month*Location	Mexico	6	6	-0.5264	0.1697	14000	-3.10	0.0019
WT	Year*Month*Location	Mexico	7	7	-0.3683	0.1670	14000	-2.21	0.0274
WT	Year*Month*Location	Mexico	8	8	-0.6438	0.1670	14000	-3.86	0.0001
WT	Year*Month*Location	Mexico	9	9	-0.5322	0.1697	14000	-3.14	0.0017
WT	Year*Month*Location	Mexico	10	10	-0.6056	0.1670	14000	-3.63	0.0003
WT	Year*Month*Location	Mexico	11	11	-0.4036	0.1697	14000	-2.38	0.0174
WT	Year*Month*Location	Mexico	12	12	-0.3233	0.1697	14000	-1.91	0.0568
WT	Year*Month*Location	Netherlands	1	1	0.0347	0.1697	14000	0.20	0.8379
WT	Year*Month*Location	Netherlands	2	2	-0.0459	0.1741	14000	-0.26	0.7921
WT	Year*Month*Location	Netherlands	3	3	-0.1788	0.1670	14000	-1.07	0.2843
WT	Year*Month*Location	Netherlands	4	4	-0.4006	0.1697	14000	-2.36	0.0183

WT	Year*Month*Location	Netherlands	5	5	-0.4051	0.1670	14000	-2.43	0.0153
WT	Year*Month*Location	Netherlands	6	6	-0.0397	0.1697	14000	-0.23	0.8149
WT	Year*Month*Location	Netherlands	7	7	-0.1812	0.1670	14000	-1.09	0.2778
WT	Year*Month*Location	Netherlands	8	8	-0.1038	0.1670	14000	-0.62	0.5343
WT	Year*Month*Location	Netherlands	9	9	-0.0914	0.1697	14000	-0.54	0.5902
WT	Year*Month*Location	Netherlands	10	10	-0.2903	0.1670	14000	-1.74	0.0821
WT	Year*Month*Location	Netherlands	11	11	-0.1753	0.1697	14000	-1.03	0.3017
WT	Year*Month*Location	Netherlands	12	12	-0.1111	0.1697	14000	-0.65	0.5127
WT	Year*Month*Location	New Zealand	1	1	0.0511	0.1697	14000	0.30	0.7633
WT	Year*Month*Location	New Zealand	2	2	0.1030	0.1741	14000	0.59	0.5540
WT	Year*Month*Location	New Zealand	3	3	-0.0223	0.1670	14000	-0.13	0.8937
WT	Year*Month*Location	New Zealand	4	4	-0.4806	0.1697	14000	-2.83	0.0046
WT	Year*Month*Location	New Zealand	5	5	-0.4091	0.1670	14000	-2.45	0.0143
WT	Year*Month*Location	New Zealand	6	6	-0.1714	0.1697	14000	-1.01	0.3126
WT	Year*Month*Location	New Zealand	7	7	-0.0441	0.1670	14000	-0.26	0.7917
WT	Year*Month*Location	New Zealand	8	8	-0.0626	0.1670	14000	-0.38	0.7075
WT	Year*Month*Location	New Zealand	9	9	-0.1064	0.1697	14000	-0.63	0.5307
WT	Year*Month*Location	New Zealand	10	10	-0.0470	0.1670	14000	-0.28	0.7781
WT	Year*Month*Location	New Zealand	11	11	-0.1606	0.1697	14000	-0.95	0.3441
WT	Year*Month*Location	New Zealand	12	12	-0.1931	0.1697	14000	-1.14	0.2553
WT	Year*Month*Location	Poland	1	1	-0.1872	0.1697	14000	-1.10	0.2700
WT	Year*Month*Location	Poland	2	2	-0.0160	0.1741	14000	-0.09	0.9269
WT	Year*Month*Location	Poland	3	3	-0.2591	0.1670	14000	-1.55	0.1206
WT	Year*Month*Location	Poland	4	4	-0.5736	0.1697	14000	-3.38	0.0007
WT	Year*Month*Location	Poland	5	5	-0.4546	0.1670	14000	-2.72	0.0065
WT	Year*Month*Location	Poland	6	6	-0.1811	0.1697	14000	-1.07	0.2859
WT	Year*Month*Location	Poland	7	7	-0.2161	0.1670	14000	-1.29	0.1955
WT	Year*Month*Location	Poland	8	8	-0.1895	0.1670	14000	-1.14	0.2563
WT	Year*Month*Location	Poland	9	9	-0.1311	0.1697	14000	-0.77	0.4398
WT	Year*Month*Location	Poland	10	10	-0.3309	0.1670	14000	-1.98	0.0475
WT	Year*Month*Location	Poland	11	11	-0.3308	0.1697	14000	-1.95	0.0513
WT	Year*Month*Location	Poland	12	12	-0.1942	0.1697	14000	-1.14	0.2526
WT	Year*Month*Location	Russia	1	1	-0.0989	0.1697	14000	-0.58	0.5601
WT	Year*Month*Location	Russia	2	2	-0.1068	0.1741	14000	-0.61	0.5396
WT	Year*Month*Location	Russia	3	3	-0.1855	0.1670	14000	-1.11	0.2666
WT	Year*Month*Location	Russia	4	4	-0.9169	0.1697	14000	-5.40	<.0001
WT	Year*Month*Location	Russia	5	5	-0.7159	0.1670	14000	-4.29	<.0001
WT	Year*Month*Location	Russia	6	6	-0.3708	0.1697	14000	-2.19	0.0289
WT	Year*Month*Location	Russia	7	7	-0.2895	0.1670	14000	-1.73	0.0829
WT	Year*Month*Location	Russia	8	8	-0.3185	0.1670	14000	-1.91	0.0564
WT	Year*Month*Location	Russia	9	9	-0.2417	0.1697	14000	-1.42	0.1545
WT	Year*Month*Location	Russia	10	10	-0.3734	0.1670	14000	-2.24	0.0253
WT	Year*Month*Location	Russia	11	11	-0.4867	0.1697	14000	-2.87	0.0041
WT	Year*Month*Location	Russia	12	12	-0.4261	0.1697	14000	-2.51	0.0121
WT	Year*Month*Location	South Africa	1	1	-0.1389	0.1697	14000	-0.82	0.4131
WT	Year*Month*Location	South Africa	2	2	-0.0566	0.1741	14000	-0.32	0.7453
WT	Year*Month*Location	South Africa	3	3	-0.1530	0.1670	14000	-0.92	0.3596
WT	Year*Month*Location	South Africa	4	4	-0.7861	0.1697	14000	-4.63	<.0001
WT	Year*Month*Location	South Africa	5	5	-0.6758	0.1670	14000	-4.05	<.0001
WT	Year*Month*Location	South Africa	6	6	-0.6078	0.1697	14000	-3.58	0.0003
WT	Year*Month*Location	South Africa	7	7	-0.6530	0.1670	14000	-3.91	<.0001
WT	Year*Month*Location	South Africa	8	8	-0.5535	0.1670	14000	-3.32	0.0009
WT	Year*Month*Location	South Africa	9	9	-0.2100	0.1697	14000	-1.24	0.2160
WT	Year*Month*Location	South Africa	10	10	-0.6000	0.1670	14000	-3.59	0.0003
WT	Year*Month*Location	South Africa	11	11	-0.3878	0.1697	14000	-2.28	0.0223
WT	Year*Month*Location	South Africa	12	12	-0.1314	0.1697	14000	-0.77	0.4388
WT	Year*Month*Location	South Korea	1	1	0.0258	0.1697	14000	0.15	0.8790
WT	Year*Month*Location	South Korea	2	2	0.1342	0.1741	14000	0.77	0.4410
WT	Year*Month*Location	South Korea	3	3	-0.1508	0.1670	14000	-0.90	0.3664
WT	Year*Month*Location	South Korea	4	4	-0.2556	0.1697	14000	-1.51	0.1321
WT	Year*Month*Location	South Korea	5	5	-0.2441	0.1670	14000	-1.46	0.1438
WT	Year*Month*Location	South Korea	6	6	0.1014	0.1697	14000	0.60	0.5502
WT	Year*Month*Location	South Korea	7	7	-0.0272	0.1670	14000	-0.16	0.8708
WT	Year*Month*Location	South Korea	8	8	-0.1051	0.1670	14000	-0.63	0.5290
WT	Year*Month*Location	South Korea	9	9	0.0517	0.1697	14000	0.30	0.7608
WT	Year*Month*Location	South Korea	10	10	-0.1522	0.1670	14000	-0.91	0.3621
WT	Year*Month*Location	South Korea	11	11	0.0814	0.1697	14000	0.48	0.6315
WT	Year*Month*Location	South Korea	12	12	-0.0222	0.1697	14000	-0.13	0.8958
WT	Year*Month*Location	Sweden	1	1	-0.0892	0.1697	14000	-0.53	0.5993
WT	Year*Month*Location	Sweden	2	2	-0.0362	0.1741	14000	-0.21	0.8353
WT	Year*Month*Location	Sweden	3	3	-0.0823	0.1670	14000	-0.49	0.6222
WT	Year*Month*Location	Sweden	4	4	-0.2208	0.1697	14000	-1.30	0.1932

WT	Year*Month*Location	Sweden	5	5	-0.2495	0.1670	14000	-1.49	0.1351
WT	Year*Month*Location	Sweden	6	6	0.0247	0.1697	14000	0.15	0.8842
WT	Year*Month*Location	Sweden	7	7	-0.1866	0.1670	14000	-1.12	0.2638
WT	Year*Month*Location	Sweden	8	8	-0.0565	0.1670	14000	-0.34	0.7353
WT	Year*Month*Location	Sweden	9	9	-0.0564	0.1697	14000	-0.33	0.7397
WT	Year*Month*Location	Sweden	10	10	-0.2188	0.1670	14000	-1.31	0.1900
WT	Year*Month*Location	Sweden	11	11	-0.2586	0.1697	14000	-1.52	0.1276
WT	Year*Month*Location	Sweden	12	12	-0.1981	0.1697	14000	-1.17	0.2432
WT	Year*Month*Location	United Kingdom	1	1	0.0414	0.1697	14000	0.24	0.8073
WT	Year*Month*Location	United Kingdom	2	2	0.0288	0.1741	14000	0.17	0.8685
WT	Year*Month*Location	United Kingdom	3	3	-0.1263	0.1670	14000	-0.76	0.4492
WT	Year*Month*Location	United Kingdom	4	4	-0.5178	0.1697	14000	-3.05	0.0023
WT	Year*Month*Location	United Kingdom	5	5	-0.5876	0.1670	14000	-3.52	0.0004
WT	Year*Month*Location	United Kingdom	6	6	-0.4514	0.1697	14000	-2.66	0.0078
WT	Year*Month*Location	United Kingdom	7	7	-0.4868	0.1670	14000	-2.92	0.0036
WT	Year*Month*Location	United Kingdom	8	8	-0.2944	0.1670	14000	-1.76	0.0779
WT	Year*Month*Location	United Kingdom	9	9	-0.1403	0.1697	14000	-0.83	0.4085
WT	Year*Month*Location	United Kingdom	10	10	-0.3121	0.1670	14000	-1.87	0.0616
WT	Year*Month*Location	United Kingdom	11	11	-0.3586	0.1697	14000	-2.11	0.0346
WT	Year*Month*Location	United Kingdom	12	12	-0.2311	0.1697	14000	-1.36	0.1733

BT	Year*Month*Location	Australia	1	1	-0.0350	0.0865	14000	-0.40	0.6857
BT	Year*Month*Location	Australia	2	2	-0.1150	0.0887	14000	-1.30	0.1951
BT	Year*Month*Location	Australia	3	3	-0.2970	0.0851	14000	-3.49	0.0005
BT	Year*Month*Location	Australia	4	4	-0.4025	0.0865	14000	-4.65	<.0001
BT	Year*Month*Location	Australia	5	5	-0.3376	0.0851	14000	-3.97	<.0001
BT	Year*Month*Location	Australia	6	6	-0.1153	0.0865	14000	-1.33	0.1826
BT	Year*Month*Location	Australia	7	7	-0.2390	0.0851	14000	-2.81	0.0050
BT	Year*Month*Location	Australia	8	8	-0.2755	0.0851	14000	-3.24	0.0012
BT	Year*Month*Location	Australia	9	9	-0.2158	0.0865	14000	-2.50	0.0126
BT	Year*Month*Location	Australia	10	10	-0.2444	0.0851	14000	-2.87	0.0041
BT	Year*Month*Location	Australia	11	11	-0.1292	0.0865	14000	-1.49	0.1353
BT	Year*Month*Location	Australia	12	12	-0.0383	0.0865	14000	-0.44	0.6576
BT	Year*Month*Location	Brazil	1	1	0.1467	0.0865	14000	1.70	0.0899
BT	Year*Month*Location	Brazil	2	2	-0.0631	0.0887	14000	-0.71	0.4773
BT	Year*Month*Location	Brazil	3	3	-0.0793	0.0851	14000	-0.93	0.3513
BT	Year*Month*Location	Brazil	4	4	-0.5308	0.0865	14000	-6.14	<.0001
BT	Year*Month*Location	Brazil	5	5	-0.5239	0.0851	14000	-6.16	<.0001
BT	Year*Month*Location	Brazil	6	6	-0.2519	0.0865	14000	-2.91	0.0036
BT	Year*Month*Location	Brazil	7	7	-0.0352	0.0851	14000	-0.41	0.6789
BT	Year*Month*Location	Brazil	8	8	-0.1578	0.0851	14000	-1.85	0.0636
BT	Year*Month*Location	Brazil	9	9	-0.1511	0.0865	14000	-1.75	0.0806
BT	Year*Month*Location	Brazil	10	10	-0.2667	0.0851	14000	-3.13	0.0017
BT	Year*Month*Location	Brazil	11	11	-0.3183	0.0865	14000	-3.68	0.0002
BT	Year*Month*Location	Brazil	12	12	-0.1133	0.0865	14000	-1.31	0.1900
BT	Year*Month*Location	Canada	1	1	-0.0228	0.0865	14000	-0.26	0.7923
BT	Year*Month*Location	Canada	2	2	-0.0482	0.0887	14000	-0.54	0.5868
BT	Year*Month*Location	Canada	3	3	-0.2691	0.0851	14000	-3.16	0.0016
BT	Year*Month*Location	Canada	4	4	-0.4592	0.0865	14000	-5.31	<.0001
BT	Year*Month*Location	Canada	5	5	-0.3828	0.0851	14000	-4.50	<.0001
BT	Year*Month*Location	Canada	6	6	-0.2406	0.0865	14000	-2.78	0.0054
BT	Year*Month*Location	Canada	7	7	-0.1581	0.0851	14000	-1.86	0.0632
BT	Year*Month*Location	Canada	8	8	-0.1917	0.0851	14000	-2.25	0.0243
BT	Year*Month*Location	Canada	9	9	-0.2533	0.0865	14000	-2.93	0.0034
BT	Year*Month*Location	Canada	10	10	-0.0890	0.0851	14000	-1.05	0.2956
BT	Year*Month*Location	Canada	11	11	-0.0797	0.0865	14000	-0.92	0.3566
BT	Year*Month*Location	Canada	12	12	-0.1131	0.0865	14000	-1.31	0.1911
BT	Year*Month*Location	China	1	1	-0.0939	0.0865	14000	-1.09	0.2776
BT	Year*Month*Location	China	2	2	-0.2458	0.0887	14000	-2.77	0.0056
BT	Year*Month*Location	China	3	3	-0.3301	0.0851	14000	-3.88	0.0001
BT	Year*Month*Location	China	4	4	-0.3242	0.0865	14000	-3.75	0.0002

BT	Year*Month*Location	China	5	5	-0.2073	0.0851	14000	-2.44	0.0149
BT	Year*Month*Location	China	6	6	-0.0825	0.0865	14000	-0.95	0.3401
BT	Year*Month*Location	China	7	7	-0.0301	0.0851	14000	-0.35	0.7234
BT	Year*Month*Location	China	8	8	-0.2798	0.0851	14000	-3.29	0.0010
BT	Year*Month*Location	China	9	9	-0.1517	0.0865	14000	-1.75	0.0795
BT	Year*Month*Location	China	10	10	-0.1344	0.0851	14000	-1.58	0.1141
BT	Year*Month*Location	China	11	11	-0.0222	0.0865	14000	-0.26	0.7972
BT	Year*Month*Location	China	12	12	-0.0169	0.0865	14000	-0.20	0.8447
BT	Year*Month*Location	Finland	1	1	-0.2581	0.0865	14000	-2.98	0.0028
BT	Year*Month*Location	Finland	2	2	-0.4086	0.0887	14000	-4.60	<.0001
BT	Year*Month*Location	Finland	3	3	-0.4804	0.0851	14000	-5.65	<.0001
BT	Year*Month*Location	Finland	4	4	-0.4650	0.0865	14000	-5.38	<.0001
BT	Year*Month*Location	Finland	5	5	-0.4562	0.0851	14000	-5.36	<.0001
BT	Year*Month*Location	Finland	6	6	-0.4386	0.0865	14000	-5.07	<.0001
BT	Year*Month*Location	Finland	7	7	-0.3444	0.0851	14000	-4.05	<.0001
BT	Year*Month*Location	Finland	8	8	-0.4312	0.0851	14000	-5.07	<.0001
BT	Year*Month*Location	Finland	9	9	-0.3933	0.0865	14000	-4.55	<.0001
BT	Year*Month*Location	Finland	10	10	-0.3804	0.0851	14000	-4.47	<.0001
BT	Year*Month*Location	Finland	11	11	-0.4094	0.0865	14000	-4.73	<.0001
BT	Year*Month*Location	Finland	12	12	-0.2206	0.0865	14000	-2.55	0.0108
BT	Year*Month*Location	France	1	1	0.0961	0.0865	14000	1.11	0.2664
BT	Year*Month*Location	France	2	2	-0.0447	0.0887	14000	-0.50	0.6148
BT	Year*Month*Location	France	3	3	-0.2594	0.0851	14000	-3.05	0.0023
BT	Year*Month*Location	France	4	4	-0.5217	0.0865	14000	-6.03	<.0001
BT	Year*Month*Location	France	5	5	-0.3083	0.0851	14000	-3.62	0.0003
BT	Year*Month*Location	France	6	6	-0.1236	0.0865	14000	-1.43	0.1529
BT	Year*Month*Location	France	7	7	-0.1390	0.0851	14000	-1.63	0.1024
BT	Year*Month*Location	France	8	8	-0.1223	0.0851	14000	-1.44	0.1505
BT	Year*Month*Location	France	9	9	-0.0697	0.0865	14000	-0.81	0.4201
BT	Year*Month*Location	France	10	10	-0.0984	0.0851	14000	-1.16	0.2475
BT	Year*Month*Location	France	11	11	-0.0958	0.0865	14000	-1.11	0.2678
BT	Year*Month*Location	France	12	12	-0.1389	0.0865	14000	-1.61	0.1083
BT	Year*Month*Location	Germany	1	1	0.0553	0.0865	14000	0.64	0.5227
BT	Year*Month*Location	Germany	2	2	-0.0350	0.0887	14000	-0.39	0.6933
BT	Year*Month*Location	Germany	3	3	-0.1306	0.0851	14000	-1.54	0.1246
BT	Year*Month*Location	Germany	4	4	-0.3086	0.0865	14000	-3.57	0.0004
BT	Year*Month*Location	Germany	5	5	-0.2753	0.0851	14000	-3.24	0.0012
BT	Year*Month*Location	Germany	6	6	-0.0453	0.0865	14000	-0.52	0.6006
BT	Year*Month*Location	Germany	7	7	-0.0863	0.0851	14000	-1.01	0.3105
BT	Year*Month*Location	Germany	8	8	-0.1409	0.0851	14000	-1.66	0.0978
BT	Year*Month*Location	Germany	9	9	-0.0589	0.0865	14000	-0.68	0.4959
BT	Year*Month*Location	Germany	10	10	-0.1309	0.0851	14000	-1.54	0.1239
BT	Year*Month*Location	Germany	11	11	-0.1358	0.0865	14000	-1.57	0.1163
BT	Year*Month*Location	Germany	12	12	-0.1400	0.0865	14000	-1.62	0.1055
BT	Year*Month*Location	India	1	1	-0.0144	0.0865	14000	-0.17	0.8674
BT	Year*Month*Location	India	2	2	-0.0629	0.0887	14000	-0.71	0.4787
BT	Year*Month*Location	India	3	3	-0.3266	0.0851	14000	-3.84	0.0001
BT	Year*Month*Location	India	4	4	-0.6203	0.0865	14000	-7.17	<.0001
BT	Year*Month*Location	India	5	5	-0.5038	0.0851	14000	-5.92	<.0001
BT	Year*Month*Location	India	6	6	-0.4708	0.0865	14000	-5.44	<.0001
BT	Year*Month*Location	India	7	7	-0.4984	0.0851	14000	-5.86	<.0001
BT	Year*Month*Location	India	8	8	-0.3852	0.0851	14000	-4.53	<.0001
BT	Year*Month*Location	India	9	9	-0.2356	0.0865	14000	-2.72	0.0065
BT	Year*Month*Location	India	10	10	-0.1444	0.0851	14000	-1.70	0.0898
BT	Year*Month*Location	India	11	11	-0.1964	0.0865	14000	-2.27	0.0232
BT	Year*Month*Location	India	12	12	0.0103	0.0865	14000	0.12	0.9054
BT	Year*Month*Location	Italy	1	1	0.0836	0.0865	14000	0.97	0.3336
BT	Year*Month*Location	Italy	2	2	0.0859	0.0887	14000	0.97	0.3332
BT	Year*Month*Location	Italy	3	3	-0.2828	0.0851	14000	-3.32	0.0009
BT	Year*Month*Location	Italy	4	4	-0.3856	0.0865	14000	-4.46	<.0001
BT	Year*Month*Location	Italy	5	5	-0.1809	0.0851	14000	-2.13	0.0335
BT	Year*Month*Location	Italy	6	6	-0.0772	0.0865	14000	-0.89	0.3719
BT	Year*Month*Location	Italy	7	7	-0.0476	0.0851	14000	-0.56	0.5760
BT	Year*Month*Location	Italy	8	8	0.0215	0.0851	14000	0.25	0.8004
BT	Year*Month*Location	Italy	9	9	-0.1103	0.0865	14000	-1.28	0.2023
BT	Year*Month*Location	Italy	10	10	-0.1718	0.0851	14000	-2.02	0.0435
BT	Year*Month*Location	Italy	11	11	-0.0925	0.0865	14000	-1.07	0.2848
BT	Year*Month*Location	Italy	12	12	-0.0378	0.0865	14000	-0.44	0.6622
BT	Year*Month*Location	Japan	1	1	0.1078	0.0865	14000	1.25	0.2127
BT	Year*Month*Location	Japan	2	2	0.1043	0.0887	14000	1.18	0.2400
BT	Year*Month*Location	Japan	3	3	0.0605	0.0851	14000	0.71	0.4771
BT	Year*Month*Location	Japan	4	4	0.0300	0.0865	14000	0.35	0.7287

BT	Year*Month*Location	Japan	5	5	0.0089	0.0851	14000	0.10	0.9170
BT	Year*Month*Location	Japan	6	6	0.0817	0.0865	14000	0.94	0.3450
BT	Year*Month*Location	Japan	7	7	0.1366	0.0851	14000	1.61	0.1085
BT	Year*Month*Location	Japan	8	8	0.0511	0.0851	14000	0.60	0.5483
BT	Year*Month*Location	Japan	9	9	0.1006	0.0865	14000	1.16	0.2449
BT	Year*Month*Location	Japan	10	10	0.1911	0.0851	14000	2.25	0.0247
BT	Year*Month*Location	Japan	11	11	0.0683	0.0865	14000	0.79	0.4294
BT	Year*Month*Location	Japan	12	12	0.1369	0.0865	14000	1.58	0.1133
BT	Year*Month*Location	Mexico	1	1	0.2408	0.0865	14000	2.78	0.0054
BT	Year*Month*Location	Mexico	2	2	0.1250	0.0887	14000	1.41	0.1591
BT	Year*Month*Location	Mexico	3	3	0.0099	0.0851	14000	0.12	0.9069
BT	Year*Month*Location	Mexico	4	4	-0.4161	0.0865	14000	-4.81	<.0001
BT	Year*Month*Location	Mexico	5	5	-0.3575	0.0851	14000	-4.20	<.0001
BT	Year*Month*Location	Mexico	6	6	-0.3842	0.0865	14000	-4.44	<.0001
BT	Year*Month*Location	Mexico	7	7	-0.1973	0.0851	14000	-2.32	0.0204
BT	Year*Month*Location	Mexico	8	8	-0.2661	0.0851	14000	-3.13	0.0018
BT	Year*Month*Location	Mexico	9	9	-0.0208	0.0865	14000	-0.24	0.8096
BT	Year*Month*Location	Mexico	10	10	-0.1059	0.0851	14000	-1.24	0.2132
BT	Year*Month*Location	Mexico	11	11	-0.0183	0.0865	14000	-0.21	0.8321
BT	Year*Month*Location	Mexico	12	12	0.1253	0.0865	14000	1.45	0.1475
BT	Year*Month*Location	Netherlands	1	1	0.0669	0.0865	14000	0.77	0.4389
BT	Year*Month*Location	Netherlands	2	2	-0.0542	0.0887	14000	-0.61	0.5413
BT	Year*Month*Location	Netherlands	3	3	-0.1164	0.0851	14000	-1.37	0.1713
BT	Year*Month*Location	Netherlands	4	4	-0.2175	0.0865	14000	-2.52	0.0119
BT	Year*Month*Location	Netherlands	5	5	-0.1503	0.0851	14000	-1.77	0.0774
BT	Year*Month*Location	Netherlands	6	6	-0.0033	0.0865	14000	-0.04	0.9693
BT	Year*Month*Location	Netherlands	7	7	-0.0508	0.0851	14000	-0.60	0.5504
BT	Year*Month*Location	Netherlands	8	8	-0.0699	0.0851	14000	-0.82	0.4113
BT	Year*Month*Location	Netherlands	9	9	-0.1486	0.0865	14000	-1.72	0.0857
BT	Year*Month*Location	Netherlands	10	10	-0.1414	0.0851	14000	-1.66	0.0965
BT	Year*Month*Location	Netherlands	11	11	-0.1125	0.0865	14000	-1.30	0.1933
BT	Year*Month*Location	Netherlands	12	12	-0.1147	0.0865	14000	-1.33	0.1847
BT	Year*Month*Location	New Zealand	1	1	0.0875	0.0865	14000	1.01	0.3117
BT	Year*Month*Location	New Zealand	2	2	0.2534	0.0887	14000	2.86	0.0043
BT	Year*Month*Location	New Zealand	3	3	-0.0758	0.0851	14000	-0.89	0.3729
BT	Year*Month*Location	New Zealand	4	4	-0.4147	0.0865	14000	-4.80	<.0001
BT	Year*Month*Location	New Zealand	5	5	-0.1769	0.0851	14000	-2.08	0.0376
BT	Year*Month*Location	New Zealand	6	6	-0.1606	0.0865	14000	-1.86	0.0634
BT	Year*Month*Location	New Zealand	7	7	-0.0559	0.0851	14000	-0.66	0.5110
BT	Year*Month*Location	New Zealand	8	8	-0.4140	0.0851	14000	-4.87	<.0001
BT	Year*Month*Location	New Zealand	9	9	-0.1878	0.0865	14000	-2.17	0.0299
BT	Year*Month*Location	New Zealand	10	10	0.0040	0.0851	14000	0.05	0.9622
BT	Year*Month*Location	New Zealand	11	11	-0.2578	0.0865	14000	-2.98	0.0029
BT	Year*Month*Location	New Zealand	12	12	0.0906	0.0865	14000	1.05	0.2951
BT	Year*Month*Location	Poland	1	1	-0.0594	0.0865	14000	-0.69	0.4919
BT	Year*Month*Location	Poland	2	2	-0.0195	0.0887	14000	-0.22	0.8257
BT	Year*Month*Location	Poland	3	3	-0.2355	0.0851	14000	-2.77	0.0056
BT	Year*Month*Location	Poland	4	4	-0.3731	0.0865	14000	-4.31	<.0001
BT	Year*Month*Location	Poland	5	5	-0.2220	0.0851	14000	-2.61	0.0091
BT	Year*Month*Location	Poland	6	6	-0.1025	0.0865	14000	-1.19	0.2359
BT	Year*Month*Location	Poland	7	7	-0.1339	0.0851	14000	-1.57	0.1156
BT	Year*Month*Location	Poland	8	8	-0.1075	0.0851	14000	-1.26	0.2063
BT	Year*Month*Location	Poland	9	9	-0.0583	0.0865	14000	-0.67	0.5000
BT	Year*Month*Location	Poland	10	10	-0.1651	0.0851	14000	-1.94	0.0524
BT	Year*Month*Location	Poland	11	11	-0.1956	0.0865	14000	-2.26	0.0238
BT	Year*Month*Location	Poland	12	12	-0.0503	0.0865	14000	-0.58	0.5610
BT	Year*Month*Location	Russia	1	1	0.0786	0.0865	14000	0.91	0.3634
BT	Year*Month*Location	Russia	2	2	0.0361	0.0887	14000	0.41	0.6839
BT	Year*Month*Location	Russia	3	3	-0.1831	0.0851	14000	-2.15	0.0314
BT	Year*Month*Location	Russia	4	4	-0.5939	0.0865	14000	-6.87	<.0001
BT	Year*Month*Location	Russia	5	5	-0.3903	0.0851	14000	-4.59	<.0001
BT	Year*Month*Location	Russia	6	6	-0.3278	0.0865	14000	-3.79	0.0002
BT	Year*Month*Location	Russia	7	7	-0.2005	0.0851	14000	-2.36	0.0184
BT	Year*Month*Location	Russia	8	8	-0.2661	0.0851	14000	-3.13	0.0018
BT	Year*Month*Location	Russia	9	9	-0.1328	0.0865	14000	-1.54	0.1247
BT	Year*Month*Location	Russia	10	10	-0.1952	0.0851	14000	-2.29	0.0218
BT	Year*Month*Location	Russia	11	11	-0.3172	0.0865	14000	-3.67	0.0002
BT	Year*Month*Location	Russia	12	12	-0.2433	0.0865	14000	-2.81	0.0049
BT	Year*Month*Location	South Africa	1	1	0.0400	0.0865	14000	0.46	0.6437
BT	Year*Month*Location	South Africa	2	2	0.0340	0.0887	14000	0.38	0.7014
BT	Year*Month*Location	South Africa	3	3	-0.0860	0.0851	14000	-1.01	0.3120
BT	Year*Month*Location	South Africa	4	4	-0.6456	0.0865	14000	-7.46	<.0001

BT	Year*Month*Location	South Africa	5	5	-0.5664	0.0851	14000	-6.66	<.0001
BT	Year*Month*Location	South Africa	6	6	-0.5400	0.0865	14000	-6.24	<.0001
BT	Year*Month*Location	South Africa	7	7	-0.4978	0.0851	14000	-5.85	<.0001
BT	Year*Month*Location	South Africa	8	8	-0.4847	0.0851	14000	-5.70	<.0001
BT	Year*Month*Location	South Africa	9	9	-0.4361	0.0865	14000	-5.04	<.0001
BT	Year*Month*Location	South Africa	10	10	-0.3927	0.0851	14000	-4.62	<.0001
BT	Year*Month*Location	South Africa	11	11	-0.3953	0.0865	14000	-4.57	<.0001
BT	Year*Month*Location	South Africa	12	12	-0.3369	0.0865	14000	-3.90	<.0001
BT	Year*Month*Location	South Korea	1	1	-0.0469	0.0865	14000	-0.54	0.5872
BT	Year*Month*Location	South Korea	2	2	0.0730	0.0887	14000	0.82	0.4109
BT	Year*Month*Location	South Korea	3	3	-0.1290	0.0851	14000	-1.52	0.1294
BT	Year*Month*Location	South Korea	4	4	-0.0369	0.0865	14000	-0.43	0.6692
BT	Year*Month*Location	South Korea	5	5	-0.0997	0.0851	14000	-1.17	0.2411
BT	Year*Month*Location	South Korea	6	6	0.1317	0.0865	14000	1.52	0.1279
BT	Year*Month*Location	South Korea	7	7	0.0460	0.0851	14000	0.54	0.5890
BT	Year*Month*Location	South Korea	8	8	-0.0347	0.0851	14000	-0.41	0.6836
BT	Year*Month*Location	South Korea	9	9	0.0803	0.0865	14000	0.93	0.3533
BT	Year*Month*Location	South Korea	10	10	-0.0430	0.0851	14000	-0.51	0.6132
BT	Year*Month*Location	South Korea	11	11	0.1611	0.0865	14000	1.86	0.0625
BT	Year*Month*Location	South Korea	12	12	0.0400	0.0865	14000	0.46	0.6437
BT	Year*Month*Location	Sweden	1	1	-0.0617	0.0865	14000	-0.71	0.4758
BT	Year*Month*Location	Sweden	2	2	-0.0052	0.0887	14000	-0.06	0.9537
BT	Year*Month*Location	Sweden	3	3	-0.1086	0.0851	14000	-1.28	0.2018
BT	Year*Month*Location	Sweden	4	4	-0.1922	0.0865	14000	-2.22	0.0262
BT	Year*Month*Location	Sweden	5	5	-0.2788	0.0851	14000	-3.28	0.0011
BT	Year*Month*Location	Sweden	6	6	-0.0222	0.0865	14000	-0.26	0.7972
BT	Year*Month*Location	Sweden	7	7	-0.1987	0.0851	14000	-2.34	0.0196
BT	Year*Month*Location	Sweden	8	8	-0.1392	0.0851	14000	-1.64	0.1017
BT	Year*Month*Location	Sweden	9	9	-0.1686	0.0865	14000	-1.95	0.0512
BT	Year*Month*Location	Sweden	10	10	-0.2503	0.0851	14000	-2.94	0.0033
BT	Year*Month*Location	Sweden	11	11	-0.1789	0.0865	14000	-2.07	0.0386
BT	Year*Month*Location	Sweden	12	12	-0.1925	0.0865	14000	-2.23	0.0260
BT	Year*Month*Location	United Kingdom	1	1	0.0272	0.0865	14000	0.31	0.7529
BT	Year*Month*Location	United Kingdom	2	2	-0.0845	0.0887	14000	-0.95	0.3409
BT	Year*Month*Location	United Kingdom	3	3	-0.1742	0.0851	14000	-2.05	0.0406
BT	Year*Month*Location	United Kingdom	4	4	-0.3406	0.0865	14000	-3.94	<.0001
BT	Year*Month*Location	United Kingdom	5	5	-0.3677	0.0851	14000	-4.32	<.0001
BT	Year*Month*Location	United Kingdom	6	6	-0.2547	0.0865	14000	-2.95	0.0032
BT	Year*Month*Location	United Kingdom	7	7	-0.2919	0.0851	14000	-3.43	0.0006
BT	Year*Month*Location	United Kingdom	8	8	-0.2226	0.0851	14000	-2.62	0.0089
BT	Year*Month*Location	United Kingdom	9	9	-0.2792	0.0865	14000	-3.23	0.0012
BT	Year*Month*Location	United Kingdom	10	10	-0.2121	0.0851	14000	-2.49	0.0127
BT	Year*Month*Location	United Kingdom	11	11	-0.2475	0.0865	14000	-2.86	0.0042
BT	Year*Month*Location	United Kingdom	12	12	-0.1922	0.0865	14000	-2.22	0.0262
TIB	Year*Month*Location	Australia	1	1	0.0125	0.1008	14000	0.12	0.9013
TIB	Year*Month*Location	Australia	2	2	0.0161	0.1035	14000	0.16	0.8766
TIB	Year*Month*Location	Australia	3	3	0.1040	0.0992	14000	1.05	0.2943
TIB	Year*Month*Location	Australia	4	4	0.0067	0.1008	14000	0.07	0.9473
TIB	Year*Month*Location	Australia	5	5	-0.1801	0.0992	14000	-1.82	0.0694
TIB	Year*Month*Location	Australia	6	6	-0.0619	0.1008	14000	-0.61	0.5390
TIB	Year*Month*Location	Australia	7	7	-0.0444	0.0992	14000	-0.45	0.6548
TIB	Year*Month*Location	Australia	8	8	-0.0212	0.0992	14000	-0.21	0.8305
TIB	Year*Month*Location	Australia	9	9	0.0222	0.1008	14000	0.22	0.8256
TIB	Year*Month*Location	Australia	10	10	-0.0261	0.0992	14000	-0.26	0.7926
TIB	Year*Month*Location	Australia	11	11	0.0275	0.1008	14000	0.27	0.7851
TIB	Year*Month*Location	Australia	12	12	0.0097	0.1008	14000	0.1	0.9232
TIB	Year*Month*Location	Brazil	1	1	0.0078	0.1008	14000	0.08	0.9385
TIB	Year*Month*Location	Brazil	2	2	0.0082	0.1035	14000	0.08	0.9367
TIB	Year*Month*Location	Brazil	3	3	0.0460	0.0992	14000	0.46	0.6431
TIB	Year*Month*Location	Brazil	4	4	-0.1769	0.1008	14000	-1.75	0.0793

TIB	Year*Month*Location	Brazil	5	5	-0.1043	0.0992	14000	-1.05	0.2930
TIB	Year*Month*Location	Brazil	6	6	-0.0097	0.1008	14000	-0.1	0.9232
TIB	Year*Month*Location	Brazil	7	7	-0.1554	0.0992	14000	-1.57	0.1173
TIB	Year*Month*Location	Brazil	8	8	-0.2707	0.0992	14000	-2.73	0.0064
TIB	Year*Month*Location	Brazil	9	9	-0.1089	0.1008	14000	-1.08	0.2802
TIB	Year*Month*Location	Brazil	10	10	-0.0417	0.0992	14000	-0.42	0.6744
TIB	Year*Month*Location	Brazil	11	11	0.0181	0.1008	14000	0.18	0.8579
TIB	Year*Month*Location	Brazil	12	12	0.0697	0.1008	14000	0.69	0.4893
TIB	Year*Month*Location	Canada	1	1	-0.0289	0.1008	14000	-0.29	0.7745
TIB	Year*Month*Location	Canada	2	2	-0.0334	0.1035	14000	-0.32	0.7467
TIB	Year*Month*Location	Canada	3	3	0.0538	0.0992	14000	0.54	0.5878
TIB	Year*Month*Location	Canada	4	4	-0.0956	0.1008	14000	-0.95	0.3433
TIB	Year*Month*Location	Canada	5	5	-0.2331	0.0992	14000	-2.35	0.0188
TIB	Year*Month*Location	Canada	6	6	-0.1408	0.1008	14000	-1.4	0.1625
TIB	Year*Month*Location	Canada	7	7	-0.1556	0.0992	14000	-1.57	0.1166
TIB	Year*Month*Location	Canada	8	8	-0.0863	0.0992	14000	-0.87	0.3843
TIB	Year*Month*Location	Canada	9	9	0.0717	0.1008	14000	0.71	0.4772
TIB	Year*Month*Location	Canada	10	10	-0.1675	0.0992	14000	-1.69	0.0913
TIB	Year*Month*Location	Canada	11	11	-0.1144	0.1008	14000	-1.14	0.2564
TIB	Year*Month*Location	Canada	12	12	-0.0833	0.1008	14000	-0.83	0.4085
TIB	Year*Month*Location	China	1	1	-0.0719	0.1008	14000	-0.71	0.4755
TIB	Year*Month*Location	China	2	2	-0.0772	0.1035	14000	-0.75	0.4558
TIB	Year*Month*Location	China	3	3	-0.0497	0.0992	14000	-0.5	0.6161
TIB	Year*Month*Location	China	4	4	-0.0833	0.1008	14000	-0.83	0.4085
TIB	Year*Month*Location	China	5	5	-0.0199	0.0992	14000	-0.2	0.8411
TIB	Year*Month*Location	China	6	6	0.0425	0.1008	14000	0.42	0.6734
TIB	Year*Month*Location	China	7	7	-0.1210	0.0992	14000	-1.22	0.2226
TIB	Year*Month*Location	China	8	8	0.2159	0.0992	14000	2.18	0.0296
TIB	Year*Month*Location	China	9	9	0.1414	0.1008	14000	1.4	0.1609
TIB	Year*Month*Location	China	10	10	0.0968	0.0992	14000	0.98	0.3292
TIB	Year*Month*Location	China	11	11	-0.0203	0.1008	14000	-0.2	0.8406
TIB	Year*Month*Location	China	12	12	0.0050	0.1008	14000	0.05	0.9605
TIB	Year*Month*Location	Finland	1	1	0.0147	0.1008	14000	0.15	0.8839
TIB	Year*Month*Location	Finland	2	2	0.1378	0.1035	14000	1.33	0.1830
TIB	Year*Month*Location	Finland	3	3	0.0825	0.0992	14000	0.83	0.4054
TIB	Year*Month*Location	Finland	4	4	-0.1319	0.1008	14000	-1.31	0.1907
TIB	Year*Month*Location	Finland	5	5	-0.1293	0.0992	14000	-1.3	0.1924
TIB	Year*Month*Location	Finland	6	6	0.0772	0.1008	14000	0.77	0.4438
TIB	Year*Month*Location	Finland	7	7	0.0374	0.0992	14000	0.38	0.7064
TIB	Year*Month*Location	Finland	8	8	0.0065	0.0992	14000	0.07	0.9481
TIB	Year*Month*Location	Finland	9	9	0.1431	0.1008	14000	1.42	0.1560
TIB	Year*Month*Location	Finland	10	10	0.0960	0.0992	14000	0.97	0.3333
TIB	Year*Month*Location	Finland	11	11	0.1450	0.1008	14000	1.44	0.1504
TIB	Year*Month*Location	Finland	12	12	0.2311	0.1008	14000	2.29	0.0219
TIB	Year*Month*Location	France	1	1	-0.0506	0.1008	14000	-0.5	0.6161
TIB	Year*Month*Location	France	2	2	0.0449	0.1035	14000	0.43	0.6647
TIB	Year*Month*Location	France	3	3	-0.0543	0.0992	14000	-0.55	0.5841
TIB	Year*Month*Location	France	4	4	-0.1950	0.1008	14000	-1.93	0.0531
TIB	Year*Month*Location	France	5	5	-0.2070	0.0992	14000	-2.09	0.0369
TIB	Year*Month*Location	France	6	6	-0.0697	0.1008	14000	-0.69	0.4893
TIB	Year*Month*Location	France	7	7	-0.0530	0.0992	14000	-0.53	0.5934
TIB	Year*Month*Location	France	8	8	0.1030	0.0992	14000	1.04	0.2993
TIB	Year*Month*Location	France	9	9	0.0289	0.1008	14000	0.29	0.7745
TIB	Year*Month*Location	France	10	10	-0.1280	0.0992	14000	-1.29	0.1971
TIB	Year*Month*Location	France	11	11	-0.1942	0.1008	14000	-1.93	0.0542
TIB	Year*Month*Location	France	12	12	-0.0631	0.1008	14000	-0.63	0.5317
TIB	Year*Month*Location	Germany	1	1	-0.0339	0.1008	14000	-0.34	0.7368
TIB	Year*Month*Location	Germany	2	2	-0.0013	0.1035	14000	-0.01	0.9899
TIB	Year*Month*Location	Germany	3	3	-0.0011	0.0992	14000	-0.01	0.9914
TIB	Year*Month*Location	Germany	4	4	-0.1756	0.1008	14000	-1.74	0.0817
TIB	Year*Month*Location	Germany	5	5	-0.2126	0.0992	14000	-2.14	0.0321
TIB	Year*Month*Location	Germany	6	6	-0.1111	0.1008	14000	-1.1	0.2705
TIB	Year*Month*Location	Germany	7	7	-0.1269	0.0992	14000	-1.28	0.2008
TIB	Year*Month*Location	Germany	8	8	-0.0046	0.0992	14000	-0.05	0.9633
TIB	Year*Month*Location	Germany	9	9	-0.0097	0.1008	14000	-0.1	0.9232
TIB	Year*Month*Location	Germany	10	10	-0.0562	0.0992	14000	-0.57	0.5711
TIB	Year*Month*Location	Germany	11	11	-0.0956	0.1008	14000	-0.95	0.3433
TIB	Year*Month*Location	Germany	12	12	-0.0783	0.1008	14000	-0.78	0.4372
TIB	Year*Month*Location	India	1	1	0.0358	0.1008	14000	0.36	0.7223
TIB	Year*Month*Location	India	2	2	0.0012	0.1035	14000	0.01	0.9911
TIB	Year*Month*Location	India	3	3	0.0172	0.0992	14000	0.17	0.8623
TIB	Year*Month*Location	India	4	4	-0.2014	0.1008	14000	-2	0.0458

TIB	Year*Month*Location	India	5	5	-0.2944	0.0992	14000	-2.97	0.0030
TIB	Year*Month*Location	India	6	6	-0.1747	0.1008	14000	-1.73	0.0831
TIB	Year*Month*Location	India	7	7	-0.3503	0.0992	14000	-3.53	0.0004
TIB	Year*Month*Location	India	8	8	-0.3755	0.0992	14000	-3.79	0.0002
TIB	Year*Month*Location	India	9	9	-0.3733	0.1008	14000	-3.7	0.0002
TIB	Year*Month*Location	India	10	10	-0.3212	0.0992	14000	-3.24	0.0012
TIB	Year*Month*Location	India	11	11	-0.2769	0.1008	14000	-2.75	0.0060
TIB	Year*Month*Location	India	12	12	-0.1233	0.1008	14000	-1.22	0.2213
TIB	Year*Month*Location	Italy	1	1	-0.0972	0.1008	14000	-0.96	0.3349
TIB	Year*Month*Location	Italy	2	2	-0.1180	0.1035	14000	-1.14	0.2541
TIB	Year*Month*Location	Italy	3	3	-0.1944	0.0992	14000	-1.96	0.0501
TIB	Year*Month*Location	Italy	4	4	-0.2267	0.1008	14000	-2.25	0.0246
TIB	Year*Month*Location	Italy	5	5	-0.3401	0.0992	14000	-3.43	0.0006
TIB	Year*Month*Location	Italy	6	6	-0.2108	0.1008	14000	-2.09	0.0365
TIB	Year*Month*Location	Italy	7	7	-0.1511	0.0992	14000	-1.52	0.1278
TIB	Year*Month*Location	Italy	8	8	-0.0500	0.0992	14000	-0.5	0.6142
TIB	Year*Month*Location	Italy	9	9	0.0067	0.1008	14000	0.07	0.9473
TIB	Year*Month*Location	Italy	10	10	-0.1820	0.0992	14000	-1.83	0.0666
TIB	Year*Month*Location	Italy	11	11	-0.1944	0.1008	14000	-1.93	0.0538
TIB	Year*Month*Location	Italy	12	12	-0.1247	0.1008	14000	-1.24	0.2161
TIB	Year*Month*Location	Japan	1	1	-0.0433	0.1008	14000	-0.43	0.6674
TIB	Year*Month*Location	Japan	2	2	-0.0620	0.1035	14000	-0.6	0.5493
TIB	Year*Month*Location	Japan	3	3	-0.0129	0.0992	14000	-0.13	0.8965
TIB	Year*Month*Location	Japan	4	4	-0.0708	0.1008	14000	-0.7	0.4824
TIB	Year*Month*Location	Japan	5	5	-0.1272	0.0992	14000	-1.28	0.1999
TIB	Year*Month*Location	Japan	6	6	-0.0469	0.1008	14000	-0.47	0.6415
TIB	Year*Month*Location	Japan	7	7	-0.1401	0.0992	14000	-1.41	0.1580
TIB	Year*Month*Location	Japan	8	8	-0.0215	0.0992	14000	-0.22	0.8284
TIB	Year*Month*Location	Japan	9	9	-0.0178	0.1008	14000	-0.18	0.8600
TIB	Year*Month*Location	Japan	10	10	-0.1306	0.0992	14000	-1.32	0.1878
TIB	Year*Month*Location	Japan	11	11	-0.0328	0.1008	14000	-0.33	0.7451
TIB	Year*Month*Location	Japan	12	12	-0.1000	0.1008	14000	-0.99	0.3213
TIB	Year*Month*Location	Mexico	1	1	-0.0150	0.1008	14000	-0.15	0.8817
TIB	Year*Month*Location	Mexico	2	2	0.0262	0.1035	14000	0.25	0.8000
TIB	Year*Month*Location	Mexico	3	3	0.0051	0.0992	14000	0.05	0.9589
TIB	Year*Month*Location	Mexico	4	4	-0.0783	0.1008	14000	-0.78	0.4372
TIB	Year*Month*Location	Mexico	5	5	-0.3648	0.0992	14000	-3.68	0.0002
TIB	Year*Month*Location	Mexico	6	6	-0.1422	0.1008	14000	-1.41	0.1584
TIB	Year*Month*Location	Mexico	7	7	-0.1710	0.0992	14000	-1.72	0.0848
TIB	Year*Month*Location	Mexico	8	8	-0.3777	0.0992	14000	-3.81	0.0001
TIB	Year*Month*Location	Mexico	9	9	-0.5114	0.1008	14000	-5.07	<.0001
TIB	Year*Month*Location	Mexico	10	10	-0.4997	0.0992	14000	-5.04	<.0001
TIB	Year*Month*Location	Mexico	11	11	-0.3853	0.1008	14000	-3.82	0.0001
TIB	Year*Month*Location	Mexico	12	12	-0.4486	0.1008	14000	-4.45	<.0001
TIB	Year*Month*Location	Netherlands	1	1	-0.0322	0.1008	14000	-0.32	0.7493
TIB	Year*Month*Location	Netherlands	2	2	0.0083	0.1035	14000	0.08	0.9360
TIB	Year*Month*Location	Netherlands	3	3	-0.0624	0.0992	14000	-0.63	0.5295
TIB	Year*Month*Location	Netherlands	4	4	-0.1831	0.1008	14000	-1.82	0.0695
TIB	Year*Month*Location	Netherlands	5	5	-0.2548	0.0992	14000	-2.57	0.0102
TIB	Year*Month*Location	Netherlands	6	6	-0.0364	0.1008	14000	-0.36	0.7182
TIB	Year*Month*Location	Netherlands	7	7	-0.1304	0.0992	14000	-1.31	0.1887
TIB	Year*Month*Location	Netherlands	8	8	-0.0339	0.0992	14000	-0.34	0.7327
TIB	Year*Month*Location	Netherlands	9	9	0.0572	0.1008	14000	0.57	0.5704
TIB	Year*Month*Location	Netherlands	10	10	-0.1489	0.0992	14000	-1.5	0.1333
TIB	Year*Month*Location	Netherlands	11	11	-0.0628	0.1008	14000	-0.62	0.5335
TIB	Year*Month*Location	Netherlands	12	12	0.0036	0.1008	14000	0.04	0.9714
TIB	Year*Month*Location	New Zealand	1	1	-0.0364	0.1008	14000	-0.36	0.7182
TIB	Year*Month*Location	New Zealand	2	2	-0.1503	0.1035	14000	-1.45	0.1463
TIB	Year*Month*Location	New Zealand	3	3	0.0535	0.0992	14000	0.54	0.5897
TIB	Year*Month*Location	New Zealand	4	4	-0.0658	0.1008	14000	-0.65	0.5138
TIB	Year*Month*Location	New Zealand	5	5	-0.2323	0.0992	14000	-2.34	0.0192
TIB	Year*Month*Location	New Zealand	6	6	-0.0108	0.1008	14000	-0.11	0.9144
TIB	Year*Month*Location	New Zealand	7	7	0.0118	0.0992	14000	0.12	0.9051
TIB	Year*Month*Location	New Zealand	8	8	0.3513	0.0992	14000	3.54	0.0004
TIB	Year*Month*Location	New Zealand	9	9	0.0814	0.1008	14000	0.81	0.4196
TIB	Year*Month*Location	New Zealand	10	10	-0.0511	0.0992	14000	-0.51	0.6066
TIB	Year*Month*Location	New Zealand	11	11	0.0972	0.1008	14000	0.96	0.3349
TIB	Year*Month*Location	New Zealand	12	12	-0.2836	0.1008	14000	-2.81	0.0049
TIB	Year*Month*Location	Poland	1	1	-0.1278	0.1008	14000	-1.27	0.2051
TIB	Year*Month*Location	Poland	2	2	0.0036	0.1035	14000	0.03	0.9725
TIB	Year*Month*Location	Poland	3	3	-0.0237	0.0992	14000	-0.24	0.8115
TIB	Year*Month*Location	Poland	4	4	-0.2006	0.1008	14000	-1.99	0.0467

TIB	Year*Month*Location	Poland	5	5	-0.2325	0.0992	14000	-2.34	0.0191
TIB	Year*Month*Location	Poland	6	6	-0.0786	0.1008	14000	-0.78	0.4356
TIB	Year*Month*Location	Poland	7	7	-0.0823	0.0992	14000	-0.83	0.4069
TIB	Year*Month*Location	Poland	8	8	-0.0820	0.0992	14000	-0.83	0.4085
TIB	Year*Month*Location	Poland	9	9	-0.0728	0.1008	14000	-0.72	0.4704
TIB	Year*Month*Location	Poland	10	10	-0.1659	0.0992	14000	-1.67	0.0945
TIB	Year*Month*Location	Poland	11	11	-0.1353	0.1008	14000	-1.34	0.1797
TIB	Year*Month*Location	Poland	12	12	-0.1439	0.1008	14000	-1.43	0.1536
TIB	Year*Month*Location	Russia	1	1	-0.1775	0.1008	14000	-1.76	0.0784
TIB	Year*Month*Location	Russia	2	2	-0.1430	0.1035	14000	-1.38	0.1670
TIB	Year*Month*Location	Russia	3	3	-0.0024	0.0992	14000	-0.02	0.9805
TIB	Year*Month*Location	Russia	4	4	-0.3231	0.1008	14000	-3.2	0.0014
TIB	Year*Month*Location	Russia	5	5	-0.3255	0.0992	14000	-3.28	0.0010
TIB	Year*Month*Location	Russia	6	6	-0.0431	0.1008	14000	-0.43	0.6694
TIB	Year*Month*Location	Russia	7	7	-0.0890	0.0992	14000	-0.9	0.3697
TIB	Year*Month*Location	Russia	8	8	-0.0524	0.0992	14000	-0.53	0.5972
TIB	Year*Month*Location	Russia	9	9	-0.1089	0.1008	14000	-1.08	0.2802
TIB	Year*Month*Location	Russia	10	10	-0.1782	0.0992	14000	-1.8	0.0724
TIB	Year*Month*Location	Russia	11	11	-0.1694	0.1008	14000	-1.68	0.0929
TIB	Year*Month*Location	Russia	12	12	-0.1828	0.1008	14000	-1.81	0.0699
TIB	Year*Month*Location	South Africa	1	1	-0.1789	0.1008	14000	-1.77	0.0761
TIB	Year*Month*Location	South Africa	2	2	-0.0906	0.1035	14000	-0.88	0.3812
TIB	Year*Month*Location	South Africa	3	3	-0.0669	0.0992	14000	-0.67	0.4998
TIB	Year*Month*Location	South Africa	4	4	-0.1406	0.1008	14000	-1.39	0.1633
TIB	Year*Month*Location	South Africa	5	5	-0.1094	0.0992	14000	-1.1	0.2700
TIB	Year*Month*Location	South Africa	6	6	-0.0678	0.1008	14000	-0.67	0.5015
TIB	Year*Month*Location	South Africa	7	7	-0.1551	0.0992	14000	-1.56	0.1179
TIB	Year*Month*Location	South Africa	8	8	-0.0688	0.0992	14000	-0.69	0.4878
TIB	Year*Month*Location	South Africa	9	9	0.2261	0.1008	14000	2.24	0.0249
TIB	Year*Month*Location	South Africa	10	10	-0.2073	0.0992	14000	-2.09	0.0367
TIB	Year*Month*Location	South Africa	11	11	0.0075	0.1008	14000	0.07	0.9407
TIB	Year*Month*Location	South Africa	12	12	0.2056	0.1008	14000	2.04	0.0415
TIB	Year*Month*Location	South Korea	1	1	0.0728	0.1008	14000	0.72	0.4704
TIB	Year*Month*Location	South Korea	2	2	0.0612	0.1035	14000	0.59	0.5541
TIB	Year*Month*Location	South Korea	3	3	-0.0218	0.0992	14000	-0.22	0.8262
TIB	Year*Month*Location	South Korea	4	4	-0.2186	0.1008	14000	-2.17	0.0302
TIB	Year*Month*Location	South Korea	5	5	-0.1444	0.0992	14000	-1.46	0.1456
TIB	Year*Month*Location	South Korea	6	6	-0.0303	0.1008	14000	-0.3	0.7640
TIB	Year*Month*Location	South Korea	7	7	-0.0731	0.0992	14000	-0.74	0.4610
TIB	Year*Month*Location	South Korea	8	8	-0.0704	0.0992	14000	-0.71	0.4777
TIB	Year*Month*Location	South Korea	9	9	-0.0286	0.1008	14000	-0.28	0.7766
TIB	Year*Month*Location	South Korea	10	10	-0.1091	0.0992	14000	-1.1	0.2712
TIB	Year*Month*Location	South Korea	11	11	-0.0797	0.1008	14000	-0.79	0.4291
TIB	Year*Month*Location	South Korea	12	12	-0.0622	0.1008	14000	-0.62	0.5372
TIB	Year*Month*Location	Sweden	1	1	-0.0275	0.1008	14000	-0.27	0.7851
TIB	Year*Month*Location	Sweden	2	2	-0.0310	0.1035	14000	-0.3	0.7641
TIB	Year*Month*Location	Sweden	3	3	0.0263	0.0992	14000	0.27	0.7906
TIB	Year*Month*Location	Sweden	4	4	-0.0286	0.1008	14000	-0.28	0.7766
TIB	Year*Month*Location	Sweden	5	5	0.0293	0.0992	14000	0.3	0.7677
TIB	Year*Month*Location	Sweden	6	6	0.0469	0.1008	14000	0.47	0.6415
TIB	Year*Month*Location	Sweden	7	7	0.0121	0.0992	14000	0.12	0.9029
TIB	Year*Month*Location	Sweden	8	8	0.0828	0.0992	14000	0.83	0.4039
TIB	Year*Month*Location	Sweden	9	9	0.1122	0.1008	14000	1.11	0.2657
TIB	Year*Month*Location	Sweden	10	10	0.0315	0.0992	14000	0.32	0.7512
TIB	Year*Month*Location	Sweden	11	11	-0.0797	0.1008	14000	-0.79	0.4291
TIB	Year*Month*Location	Sweden	12	12	-0.0056	0.1008	14000	-0.06	0.9561
TIB	Year*Month*Location	United Kingdom	1	1	0.0142	0.1008	14000	0.14	0.8883
TIB	Year*Month*Location	United Kingdom	2	2	0.1133	0.1035	14000	1.1	0.2733
TIB	Year*Month*Location	United Kingdom	3	3	0.0479	0.0992	14000	0.48	0.6295
TIB	Year*Month*Location	United Kingdom	4	4	-0.1772	0.1008	14000	-1.76	0.0788
TIB	Year*Month*Location	United Kingdom	5	5	-0.2199	0.0992	14000	-2.22	0.0266
TIB	Year*Month*Location	United Kingdom	6	6	-0.1967	0.1008	14000	-1.95	0.0511
TIB	Year*Month*Location	United Kingdom	7	7	-0.1949	0.0992	14000	-1.96	0.0494
TIB	Year*Month*Location	United Kingdom	8	8	-0.0718	0.0992	14000	-0.72	0.4693
TIB	Year*Month*Location	United Kingdom	9	9	0.1389	0.1008	14000	1.38	0.1684
TIB	Year*Month*Location	United Kingdom	10	10	-0.1000	0.0992	14000	-1.01	0.3134
TIB	Year*Month*Location	United Kingdom	11	11	-0.1111	0.1008	14000	-1.1	0.2705
TIB	Year*Month*Location	United Kingdom	12	12	-0.0389	0.1008	14000	-0.39	0.6997

MOS	Year*Month*Location	Australia	1	1	-0.0288	0.1249	14000	-0.23	0.8179
MOS	Year*Month*Location	Australia	2	2	-0.1069	0.1282	14000	-0.83	0.4041
MOS	Year*Month*Location	Australia	3	3	-0.2450	0.1229	14000	-1.99	0.0461
MOS	Year*Month*Location	Australia	4	4	-0.3992	0.1249	14000	-3.2	0.0014
MOS	Year*Month*Location	Australia	5	5	-0.4277	0.1229	14000	-3.48	0.0005
MOS	Year*Month*Location	Australia	6	6	-0.1462	0.1249	14000	-1.17	0.2416
MOS	Year*Month*Location	Australia	7	7	-0.2612	0.1229	14000	-2.13	0.0336
MOS	Year*Month*Location	Australia	8	8	-0.2862	0.1229	14000	-2.33	0.0199
MOS	Year*Month*Location	Australia	9	9	-0.2047	0.1249	14000	-1.64	0.1012
MOS	Year*Month*Location	Australia	10	10	-0.2574	0.1229	14000	-2.09	0.0362
MOS	Year*Month*Location	Australia	11	11	-0.1154	0.1249	14000	-0.92	0.3554
MOS	Year*Month*Location	Australia	12	12	-0.0335	0.1249	14000	-0.27	0.7887
MOS	Year*Month*Location	Brazil	1	1	0.1506	0.1249	14000	1.21	0.2280
MOS	Year*Month*Location	Brazil	2	2	-0.0590	0.1282	14000	-0.46	0.6455
MOS	Year*Month*Location	Brazil	3	3	-0.0563	0.1229	14000	-0.46	0.6467
MOS	Year*Month*Location	Brazil	4	4	-0.6193	0.1249	14000	-4.96	<.0001
MOS	Year*Month*Location	Brazil	5	5	-0.5761	0.1229	14000	-4.69	<.0001
MOS	Year*Month*Location	Brazil	6	6	-0.2568	0.1249	14000	-2.06	0.0398
MOS	Year*Month*Location	Brazil	7	7	-0.1129	0.1229	14000	-0.92	0.3581
MOS	Year*Month*Location	Brazil	8	8	-0.2931	0.1229	14000	-2.39	0.0170
MOS	Year*Month*Location	Brazil	9	9	-0.2056	0.1249	14000	-1.65	0.0998
MOS	Year*Month*Location	Brazil	10	10	-0.2875	0.1229	14000	-2.34	0.0193
MOS	Year*Month*Location	Brazil	11	11	-0.3093	0.1249	14000	-2.48	0.0133
MOS	Year*Month*Location	Brazil	12	12	-0.0785	0.1249	14000	-0.63	0.5298
MOS	Year*Month*Location	Canada	1	1	-0.0372	0.1249	14000	-0.3	0.7657
MOS	Year*Month*Location	Canada	2	2	-0.0649	0.1282	14000	-0.51	0.6123
MOS	Year*Month*Location	Canada	3	3	-0.2422	0.1229	14000	-1.97	0.0487
MOS	Year*Month*Location	Canada	4	4	-0.5069	0.1249	14000	-4.06	<.0001
MOS	Year*Month*Location	Canada	5	5	-0.4993	0.1229	14000	-4.06	<.0001
MOS	Year*Month*Location	Canada	6	6	-0.3110	0.1249	14000	-2.49	0.0128
MOS	Year*Month*Location	Canada	7	7	-0.2359	0.1229	14000	-1.92	0.0549
MOS	Year*Month*Location	Canada	8	8	-0.2348	0.1229	14000	-1.91	0.0560
MOS	Year*Month*Location	Canada	9	9	-0.2175	0.1249	14000	-1.74	0.0816
MOS	Year*Month*Location	Canada	10	10	-0.1727	0.1229	14000	-1.41	0.1598
MOS	Year*Month*Location	Canada	11	11	-0.1369	0.1249	14000	-1.1	0.2729
MOS	Year*Month*Location	Canada	12	12	-0.1547	0.1249	14000	-1.24	0.2154
MOS	Year*Month*Location	China	1	1	-0.1299	0.1249	14000	-1.04	0.2985
MOS	Year*Month*Location	China	2	2	-0.2844	0.1282	14000	-2.22	0.0265
MOS	Year*Month*Location	China	3	3	-0.3550	0.1229	14000	-2.89	0.0039
MOS	Year*Month*Location	China	4	4	-0.3658	0.1249	14000	-2.93	0.0034
MOS	Year*Month*Location	China	5	5	-0.2172	0.1229	14000	-1.77	0.0771
MOS	Year*Month*Location	China	6	6	-0.0613	0.1249	14000	-0.49	0.6238
MOS	Year*Month*Location	China	7	7	-0.0906	0.1229	14000	-0.74	0.4609
MOS	Year*Month*Location	China	8	8	-0.1719	0.1229	14000	-1.4	0.1618
MOS	Year*Month*Location	China	9	9	-0.0810	0.1249	14000	-0.65	0.5168
MOS	Year*Month*Location	China	10	10	-0.0860	0.1229	14000	-0.7	0.4839
MOS	Year*Month*Location	China	11	11	-0.0324	0.1249	14000	-0.26	0.7956
MOS	Year*Month*Location	China	12	12	-0.0144	0.1249	14000	-0.12	0.9079
MOS	Year*Month*Location	Finland	1	1	-0.2507	0.1249	14000	-2.01	0.0447
MOS	Year*Month*Location	Finland	2	2	-0.3398	0.1282	14000	-2.65	0.0080
MOS	Year*Month*Location	Finland	3	3	-0.4391	0.1229	14000	-3.57	0.0004
MOS	Year*Month*Location	Finland	4	4	-0.5310	0.1249	14000	-4.25	<.0001
MOS	Year*Month*Location	Finland	5	5	-0.5208	0.1229	14000	-4.24	<.0001
MOS	Year*Month*Location	Finland	6	6	-0.4000	0.1249	14000	-3.2	0.0014
MOS	Year*Month*Location	Finland	7	7	-0.3257	0.1229	14000	-2.65	0.0080
MOS	Year*Month*Location	Finland	8	8	-0.4280	0.1229	14000	-3.48	0.0005
MOS	Year*Month*Location	Finland	9	9	-0.3218	0.1249	14000	-2.58	0.0100
MOS	Year*Month*Location	Finland	10	10	-0.3324	0.1229	14000	-2.71	0.0068
MOS	Year*Month*Location	Finland	11	11	-0.3369	0.1249	14000	-2.7	0.0070
MOS	Year*Month*Location	Finland	12	12	-0.1050	0.1249	14000	-0.84	0.4005
MOS	Year*Month*Location	France	1	1	0.0708	0.1249	14000	0.57	0.5706
MOS	Year*Month*Location	France	2	2	-0.0222	0.1282	14000	-0.17	0.8623
MOS	Year*Month*Location	France	3	3	-0.2866	0.1229	14000	-2.33	0.0197
MOS	Year*Month*Location	France	4	4	-0.6192	0.1249	14000	-4.96	<.0001

MOS	Year*Month*Location	France	5	5	-0.4118	0.1229	14000	-3.35	0.0008
MOS	Year*Month*Location	France	6	6	-0.1585	0.1249	14000	-1.27	0.2045
MOS	Year*Month*Location	France	7	7	-0.1655	0.1229	14000	-1.35	0.1781
MOS	Year*Month*Location	France	8	8	-0.0708	0.1229	14000	-0.58	0.5643
MOS	Year*Month*Location	France	9	9	-0.0553	0.1249	14000	-0.44	0.6581
MOS	Year*Month*Location	France	10	10	-0.1624	0.1229	14000	-1.32	0.1864
MOS	Year*Month*Location	France	11	11	-0.1929	0.1249	14000	-1.54	0.1225
MOS	Year*Month*Location	France	12	12	-0.1704	0.1249	14000	-1.36	0.1724
MOS	Year*Month*Location	Germany	1	1	0.0383	0.1249	14000	0.31	0.7589
MOS	Year*Month*Location	Germany	2	2	-0.0357	0.1282	14000	-0.28	0.7809
MOS	Year*Month*Location	Germany	3	3	-0.1312	0.1229	14000	-1.07	0.2857
MOS	Year*Month*Location	Germany	4	4	-0.3964	0.1249	14000	-3.17	0.0015
MOS	Year*Month*Location	Germany	5	5	-0.3816	0.1229	14000	-3.11	0.0019
MOS	Year*Month*Location	Germany	6	6	-0.1008	0.1249	14000	-0.81	0.4195
MOS	Year*Month*Location	Germany	7	7	-0.1497	0.1229	14000	-1.22	0.2230
MOS	Year*Month*Location	Germany	8	8	-0.1431	0.1229	14000	-1.17	0.2440
MOS	Year*Month*Location	Germany	9	9	-0.0638	0.1249	14000	-0.51	0.6098
MOS	Year*Month*Location	Germany	10	10	-0.1590	0.1229	14000	-1.29	0.1956
MOS	Year*Month*Location	Germany	11	11	-0.1836	0.1249	14000	-1.47	0.1415
MOS	Year*Month*Location	Germany	12	12	-0.1792	0.1249	14000	-1.43	0.1514
MOS	Year*Month*Location	India	1	1	0.0035	0.1249	14000	0.03	0.9778
MOS	Year*Month*Location	India	2	2	-0.0623	0.1282	14000	-0.49	0.6270
MOS	Year*Month*Location	India	3	3	-0.3180	0.1229	14000	-2.59	0.0097
MOS	Year*Month*Location	India	4	4	-0.7210	0.1249	14000	-5.77	<.0001
MOS	Year*Month*Location	India	5	5	-0.6509	0.1229	14000	-5.3	<.0001
MOS	Year*Month*Location	India	6	6	-0.5582	0.1249	14000	-4.47	<.0001
MOS	Year*Month*Location	India	7	7	-0.6735	0.1229	14000	-5.48	<.0001
MOS	Year*Month*Location	India	8	8	-0.5730	0.1229	14000	-4.66	<.0001
MOS	Year*Month*Location	India	9	9	-0.4222	0.1249	14000	-3.38	0.0007
MOS	Year*Month*Location	India	10	10	-0.3050	0.1229	14000	-2.48	0.0131
MOS	Year*Month*Location	India	11	11	-0.3349	0.1249	14000	-2.68	0.0073
MOS	Year*Month*Location	India	12	12	-0.0514	0.1249	14000	-0.41	0.6807
MOS	Year*Month*Location	Italy	1	1	0.0350	0.1249	14000	0.28	0.7793
MOS	Year*Month*Location	Italy	2	2	0.0269	0.1282	14000	0.21	0.8339
MOS	Year*Month*Location	Italy	3	3	-0.3800	0.1229	14000	-3.09	0.0020
MOS	Year*Month*Location	Italy	4	4	-0.4989	0.1249	14000	-3.99	<.0001
MOS	Year*Month*Location	Italy	5	5	-0.3509	0.1229	14000	-2.86	0.0043
MOS	Year*Month*Location	Italy	6	6	-0.1826	0.1249	14000	-1.46	0.1437
MOS	Year*Month*Location	Italy	7	7	-0.1231	0.1229	14000	-1	0.3163
MOS	Year*Month*Location	Italy	8	8	-0.0035	0.1229	14000	-0.03	0.9773
MOS	Year*Month*Location	Italy	9	9	-0.1069	0.1249	14000	-0.86	0.3919
MOS	Year*Month*Location	Italy	10	10	-0.2628	0.1229	14000	-2.14	0.0325
MOS	Year*Month*Location	Italy	11	11	-0.1897	0.1249	14000	-1.52	0.1288
MOS	Year*Month*Location	Italy	12	12	-0.1001	0.1249	14000	-0.8	0.4227
MOS	Year*Month*Location	Japan	1	1	0.0861	0.1249	14000	0.69	0.4905
MOS	Year*Month*Location	Japan	2	2	0.0733	0.1282	14000	0.57	0.5674
MOS	Year*Month*Location	Japan	3	3	0.0540	0.1229	14000	0.44	0.6601
MOS	Year*Month*Location	Japan	4	4	-0.0054	0.1249	14000	-0.04	0.9654
MOS	Year*Month*Location	Japan	5	5	-0.0547	0.1229	14000	-0.45	0.6561
MOS	Year*Month*Location	Japan	6	6	0.0582	0.1249	14000	0.47	0.6413
MOS	Year*Month*Location	Japan	7	7	0.0665	0.1229	14000	0.54	0.5882
MOS	Year*Month*Location	Japan	8	8	0.0403	0.1229	14000	0.33	0.7428
MOS	Year*Month*Location	Japan	9	9	0.0917	0.1249	14000	0.73	0.4630
MOS	Year*Month*Location	Japan	10	10	0.1258	0.1229	14000	1.02	0.3059
MOS	Year*Month*Location	Japan	11	11	0.0519	0.1249	14000	0.42	0.6775
MOS	Year*Month*Location	Japan	12	12	0.0869	0.1249	14000	0.7	0.4863
MOS	Year*Month*Location	Mexico	1	1	0.2333	0.1249	14000	1.87	0.0617
MOS	Year*Month*Location	Mexico	2	2	0.1381	0.1282	14000	1.08	0.2813
MOS	Year*Month*Location	Mexico	3	3	0.0125	0.1229	14000	0.1	0.9190
MOS	Year*Month*Location	Mexico	4	4	-0.4553	0.1249	14000	-3.65	0.0003
MOS	Year*Month*Location	Mexico	5	5	-0.5399	0.1229	14000	-4.39	<.0001
MOS	Year*Month*Location	Mexico	6	6	-0.4553	0.1249	14000	-3.65	0.0003
MOS	Year*Month*Location	Mexico	7	7	-0.2828	0.1229	14000	-2.3	0.0214
MOS	Year*Month*Location	Mexico	8	8	-0.4550	0.1229	14000	-3.7	0.0002
MOS	Year*Month*Location	Mexico	9	9	-0.2765	0.1249	14000	-2.21	0.0268
MOS	Year*Month*Location	Mexico	10	10	-0.3558	0.1229	14000	-2.9	0.0038
MOS	Year*Month*Location	Mexico	11	11	-0.2110	0.1249	14000	-1.69	0.0912
MOS	Year*Month*Location	Mexico	12	12	-0.0990	0.1249	14000	-0.79	0.4279
MOS	Year*Month*Location	Netherlands	1	1	0.0508	0.1249	14000	0.41	0.6840
MOS	Year*Month*Location	Netherlands	2	2	-0.0501	0.1282	14000	-0.39	0.6961
MOS	Year*Month*Location	Netherlands	3	3	-0.1476	0.1229	14000	-1.2	0.2297
MOS	Year*Month*Location	Netherlands	4	4	-0.3090	0.1249	14000	-2.47	0.0134

MOS	Year*Month*Location	Netherlands	5	5	-0.2777	0.1229	14000	-2.26	0.0238
MOS	Year*Month*Location	Netherlands	6	6	-0.0215	0.1249	14000	-0.17	0.8632
MOS	Year*Month*Location	Netherlands	7	7	-0.1160	0.1229	14000	-0.94	0.3451
MOS	Year*Month*Location	Netherlands	8	8	-0.0868	0.1229	14000	-0.71	0.4798
MOS	Year*Month*Location	Netherlands	9	9	-0.1200	0.1249	14000	-0.96	0.3367
MOS	Year*Month*Location	Netherlands	10	10	-0.2159	0.1229	14000	-1.76	0.0790
MOS	Year*Month*Location	Netherlands	11	11	-0.1439	0.1249	14000	-1.15	0.2493
MOS	Year*Month*Location	Netherlands	12	12	-0.1129	0.1249	14000	-0.9	0.3660
MOS	Year*Month*Location	New Zealand	1	1	0.0693	0.1249	14000	0.55	0.5790
MOS	Year*Month*Location	New Zealand	2	2	0.1782	0.1282	14000	1.39	0.1644
MOS	Year*Month*Location	New Zealand	3	3	-0.0491	0.1229	14000	-0.4	0.6897
MOS	Year*Month*Location	New Zealand	4	4	-0.4476	0.1249	14000	-3.58	0.0003
MOS	Year*Month*Location	New Zealand	5	5	-0.2930	0.1229	14000	-2.38	0.0171
MOS	Year*Month*Location	New Zealand	6	6	-0.1660	0.1249	14000	-1.33	0.1839
MOS	Year*Month*Location	New Zealand	7	7	-0.0500	0.1229	14000	-0.41	0.6840
MOS	Year*Month*Location	New Zealand	8	8	-0.2383	0.1229	14000	-1.94	0.0524
MOS	Year*Month*Location	New Zealand	9	9	-0.1471	0.1249	14000	-1.18	0.2390
MOS	Year*Month*Location	New Zealand	10	10	-0.0215	0.1229	14000	-0.18	0.8611
MOS	Year*Month*Location	New Zealand	11	11	-0.2092	0.1249	14000	-1.67	0.0940
MOS	Year*Month*Location	New Zealand	12	12	-0.0513	0.1249	14000	-0.41	0.6816
MOS	Year*Month*Location	Poland	1	1	-0.1233	0.1249	14000	-0.99	0.3234
MOS	Year*Month*Location	Poland	2	2	-0.0178	0.1282	14000	-0.14	0.8898
MOS	Year*Month*Location	Poland	3	3	-0.2473	0.1229	14000	-2.01	0.0441
MOS	Year*Month*Location	Poland	4	4	-0.4733	0.1249	14000	-3.79	0.0002
MOS	Year*Month*Location	Poland	5	5	-0.3383	0.1229	14000	-2.75	0.0059
MOS	Year*Month*Location	Poland	6	6	-0.1418	0.1249	14000	-1.14	0.2562
MOS	Year*Month*Location	Poland	7	7	-0.1750	0.1229	14000	-1.42	0.1544
MOS	Year*Month*Location	Poland	8	8	-0.1485	0.1229	14000	-1.21	0.2267
MOS	Year*Month*Location	Poland	9	9	-0.0947	0.1249	14000	-0.76	0.4482
MOS	Year*Month*Location	Poland	10	10	-0.2480	0.1229	14000	-2.02	0.0436
MOS	Year*Month*Location	Poland	11	11	-0.2632	0.1249	14000	-2.11	0.0351
MOS	Year*Month*Location	Poland	12	12	-0.1222	0.1249	14000	-0.98	0.3278
MOS	Year*Month*Location	Russia	1	1	-0.0101	0.1249	14000	-0.08	0.9353
MOS	Year*Month*Location	Russia	2	2	-0.0354	0.1282	14000	-0.28	0.7827
MOS	Year*Month*Location	Russia	3	3	-0.1843	0.1229	14000	-1.5	0.1337
MOS	Year*Month*Location	Russia	4	4	-0.7554	0.1249	14000	-6.05	<.0001
MOS	Year*Month*Location	Russia	5	5	-0.5531	0.1229	14000	-4.5	<.0001
MOS	Year*Month*Location	Russia	6	6	-0.3493	0.1249	14000	-2.8	0.0052
MOS	Year*Month*Location	Russia	7	7	-0.2450	0.1229	14000	-1.99	0.0461
MOS	Year*Month*Location	Russia	8	8	-0.2923	0.1229	14000	-2.38	0.0174
MOS	Year*Month*Location	Russia	9	9	-0.1872	0.1249	14000	-1.5	0.1339
MOS	Year*Month*Location	Russia	10	10	-0.2843	0.1229	14000	-2.31	0.0207
MOS	Year*Month*Location	Russia	11	11	-0.4019	0.1249	14000	-3.22	0.0013
MOS	Year*Month*Location	Russia	12	12	-0.3347	0.1249	14000	-2.68	0.0074
MOS	Year*Month*Location	South Africa	1	1	-0.0494	0.1249	14000	-0.4	0.6922
MOS	Year*Month*Location	South Africa	2	2	-0.0113	0.1282	14000	-0.09	0.9299
MOS	Year*Month*Location	South Africa	3	3	-0.1195	0.1229	14000	-0.97	0.3308
MOS	Year*Month*Location	South Africa	4	4	-0.7158	0.1249	14000	-5.73	<.0001
MOS	Year*Month*Location	South Africa	5	5	-0.6211	0.1229	14000	-5.06	<.0001
MOS	Year*Month*Location	South Africa	6	6	-0.5739	0.1249	14000	-4.6	<.0001
MOS	Year*Month*Location	South Africa	7	7	-0.5754	0.1229	14000	-4.68	<.0001
MOS	Year*Month*Location	South Africa	8	8	-0.5191	0.1229	14000	-4.22	<.0001
MOS	Year*Month*Location	South Africa	9	9	-0.3231	0.1249	14000	-2.59	0.0097
MOS	Year*Month*Location	South Africa	10	10	-0.4964	0.1229	14000	-4.04	<.0001
MOS	Year*Month*Location	South Africa	11	11	-0.3915	0.1249	14000	-3.13	0.0017
MOS	Year*Month*Location	South Africa	12	12	-0.2342	0.1249	14000	-1.87	0.0608
MOS	Year*Month*Location	South Korea	1	1	-0.0106	0.1249	14000	-0.08	0.9326
MOS	Year*Month*Location	South Korea	2	2	0.1036	0.1282	14000	0.81	0.4190
MOS	Year*Month*Location	South Korea	3	3	-0.1399	0.1229	14000	-1.14	0.2548
MOS	Year*Month*Location	South Korea	4	4	-0.1462	0.1249	14000	-1.17	0.2416
MOS	Year*Month*Location	South Korea	5	5	-0.1719	0.1229	14000	-1.4	0.1618
MOS	Year*Month*Location	South Korea	6	6	0.1165	0.1249	14000	0.93	0.3508
MOS	Year*Month*Location	South Korea	7	7	0.0094	0.1229	14000	0.08	0.9390
MOS	Year*Month*Location	South Korea	8	8	-0.0699	0.1229	14000	-0.57	0.5695
MOS	Year*Month*Location	South Korea	9	9	0.0660	0.1249	14000	0.53	0.5974
MOS	Year*Month*Location	South Korea	10	10	-0.0976	0.1229	14000	-0.79	0.4271
MOS	Year*Month*Location	South Korea	11	11	0.1212	0.1249	14000	0.97	0.3317
MOS	Year*Month*Location	South Korea	12	12	0.0089	0.1249	14000	0.07	0.9433
MOS	Year*Month*Location	Sweden	1	1	-0.0754	0.1249	14000	-0.6	0.5460
MOS	Year*Month*Location	Sweden	2	2	-0.0207	0.1282	14000	-0.16	0.8718
MOS	Year*Month*Location	Sweden	3	3	-0.0954	0.1229	14000	-0.78	0.4373
MOS	Year*Month*Location	Sweden	4	4	-0.2065	0.1249	14000	-1.65	0.0982

MOS	Year*Month*Location	Sweden	5	5	-0.2641	0.1229	14000	-2.15	0.0316
MOS	Year*Month*Location	Sweden	6	6	0.0013	0.1249	14000	0.01	0.9920
MOS	Year*Month*Location	Sweden	7	7	-0.1926	0.1229	14000	-1.57	0.1170
MOS	Year*Month*Location	Sweden	8	8	-0.0979	0.1229	14000	-0.8	0.4258
MOS	Year*Month*Location	Sweden	9	9	-0.1125	0.1249	14000	-0.9	0.3677
MOS	Year*Month*Location	Sweden	10	10	-0.2345	0.1229	14000	-1.91	0.0563
MOS	Year*Month*Location	Sweden	11	11	-0.2188	0.1249	14000	-1.75	0.0799
MOS	Year*Month*Location	Sweden	12	12	-0.1953	0.1249	14000	-1.56	0.1179
MOS	Year*Month*Location	United Kingdom	1	1	0.0343	0.1249	14000	0.27	0.7836
MOS	Year*Month*Location	United Kingdom	2	2	-0.0278	0.1282	14000	-0.22	0.8280
MOS	Year*Month*Location	United Kingdom	3	3	-0.1503	0.1229	14000	-1.22	0.2213
MOS	Year*Month*Location	United Kingdom	4	4	-0.4292	0.1249	14000	-3.44	0.0006
MOS	Year*Month*Location	United Kingdom	5	5	-0.4777	0.1229	14000	-3.89	0.0001
MOS	Year*Month*Location	United Kingdom	6	6	-0.3531	0.1249	14000	-2.83	0.0047
MOS	Year*Month*Location	United Kingdom	7	7	-0.3894	0.1229	14000	-3.17	0.0015
MOS	Year*Month*Location	United Kingdom	8	8	-0.2585	0.1229	14000	-2.1	0.0354
MOS	Year*Month*Location	United Kingdom	9	9	-0.2097	0.1249	14000	-1.68	0.0931
MOS	Year*Month*Location	United Kingdom	10	10	-0.2621	0.1229	14000	-2.13	0.0329
MOS	Year*Month*Location	United Kingdom	11	11	-0.3031	0.1249	14000	-2.43	0.0153
MOS	Year*Month*Location	United Kingdom	12	12	-0.2117	0.1249	14000	-1.69	0.0901
